# Cyclometalated Platinum Compounds from Competing C–H/C–X Bond Activation Pathways

**DOI:** 10.1021/acsomega.5c12884

**Published:** 2026-03-03

**Authors:** Craig M. Anderson, Matthew W. Greenberg, Christopher N. LaFratta, Monika Dziubelski, Zainab Aleem, Benett B. Hathaway, Joseph M. Tanski

**Affiliations:** † Department of Chemistry & Biochemistry, 1263 Bard College, 30 Campus Road, Annandale-on-Hudson, New York 12504, United States; ‡ Department of Chemistry, 6711 Vassar College, Poughkeepsie, New York 12604, United States

## Abstract

X–C^N^N (X = Br, Cl) ligands were reacted with [Pt_2_Me_4_(μ-SMe_2_)_2_], **1**, resulting in a six-coordinate cyclometalated platinum­(IV) compound containing an anionic C^N^N ligand when X = Br and both a platinum­(IV) and a platinum­(II) product when X = Cl. The platinum­(II) species was formed by C–H activation, followed by reductive elimination of methane. The platinum compounds were characterized by multinuclear NMR spectroscopy and single-crystal X-ray diffraction (SCXRD). Photophysical properties were explored by using UV/vis, emission, and transient absorption (TA) spectroscopies. DFT and TDDFT calculations were performed to examine the competition between C–H activation and C–X oxidative addition and compared to experimental results.

## Graphical abstract

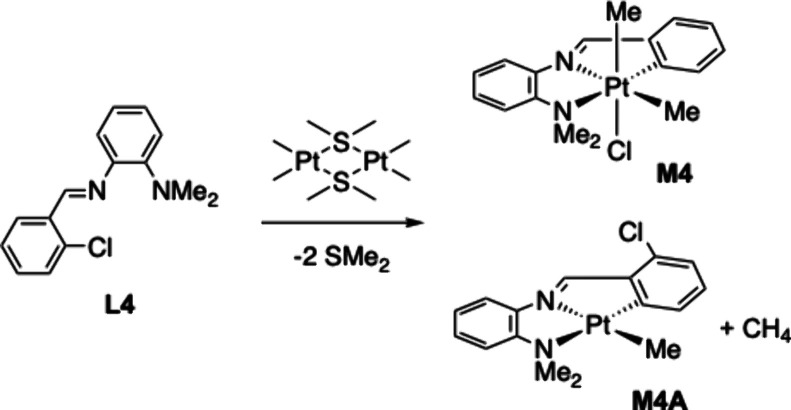

## Introduction

Oxidative addition and reductive elimination are fundamental organometallic reactions that are observed in many catalytic cycles and stoichiometric reactions.^1–7^ Additionally, selective C–H bond activation to form orthometalated species is a subject in organometallic chemistry that has been studied in depth.^8–11^ Ligands containing ortho *sp* ^2^ C–X and C–H bonds have been shown to react with the platinum tetramethyl dimer [Pt_2_Me_4_(μ-SMe_2_)_2_], **1**,^12^ and follow a reactivity trend inverse to their bond strength: C–Br > C–Cl > C–H > C–F.^13,14^ Ortho C–Br and C–Cl bonds almost exclusively react faster and are selectively broken before any competing ortho C–H bonds.^13,14^ The *N*-benzylidenebenzylamine ligand 2,4-Cl_2_C_6_H_3_CH=NCH_2_C_6_H_5_, **2**, where a competition between ortho C–H and C–Cl bonds exists, produces a product mixture of both platinum­(II) and platinum­(IV) species when reacted with **1**.^15^ The close reactivity between C–H and C–Cl bonds was attributed to the existence of a second electron-withdrawing chloride ligand on its metalated benzene ring. The situation where both C–H and C–Cl activation are observed from the same ligand appears to be somewhat rare and not well explored.^16,17^ The reactivity of C–X (X = Cl, Br, H) to platinum­(II) was reviewed approximately a decade ago.^18^ Other related work that has been reported recently includes regioselective competition in rollover compounds and regioselective C–H/C–Cl bond activation competition for intermolecular reactions.^19,20^ In this paper, we examine the reactivity of ortho C–X and C–H bonds in direct competition with each other with respect to the oxidative addition reaction to dimethyl platinum­(II).

Cyclometalated platinum species are very well known for their photophysical properties.^21,22^ Platinum­(II) is the most explored of the oxidation states of such compounds.^23–32^ Platinum­(IV) species are much less studied, but a few interesting examples do exist.^33,34^ Moreover, dual emitting compounds, defined as compounds that emit at two distinct frequencies, are sought after for many reasons, including their use as ratiometric sensors.^35–37^ Herein, we report the synthesis and characterization of platinum species made with specifically designed heteroatom ligands, which facilitate chelate-assisted C–X or C–H oxidative addition, and examine the competition between carbon halide and carbon hydrogen bond activation. These ligands were reacted with the platinum tetramethyl dimer, **1**, to give platinum­(IV) species that include a planar, pincer, anionic N^N^C ligand. The bromo ligand analogue exclusively formed the C–Br oxidative addition platinum­(IV) product, while the chloro ligand derivative formed both a platinum­(IV) and a platinum­(II) product, formed by C–Cl oxidative addition and C–H activation/reductive elimination, respectively. Moreover, the bromo platinum­(IV) species was emissive in solution at room temperature, and multiple bands were observed in its spectrum.

## Results and Discussion

### Synthesis and Characterization

Appropriately designed ligands **L3** and **L4** were synthesized by a simple condensation reaction between 2-bromobenzaldehyde or 2-chlorobenzaldehyde and the primary amine, dimethylamino aniline, to result in pincer imine ligands that can coordinate through the nitrogen imine atom and thus facilitate chelate-assisted oxidative addition. The two ligands were characterized by their proton NMR spectra. The ligands, when reacted with the platinum dimer, **1**, afforded exclusively a platinum­(IV) product, **M3**, with the bromo derivative ligand, **L3**, (Scheme 1) and a mixture of platinum­(IV) and platinum­(II) products, **M4** and **M4A**, with the chloro derivative ligand, **L4** (Scheme 2). The platinum compounds were characterized by multinuclear and multidimensional NMR spectroscopy, emission spectroscopy, single-crystal X-ray diffraction (SCXRD), and high-resolution mass spectrometry.

**Scheme 1:**
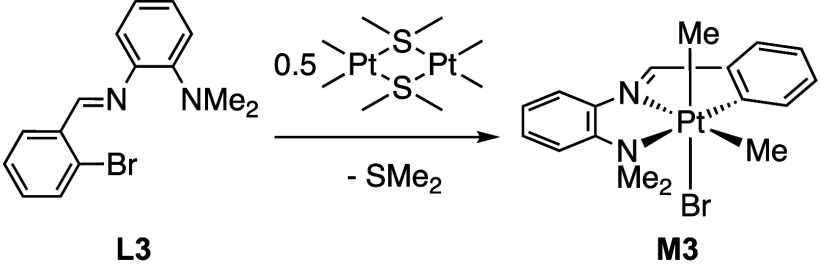
Reaction of **L3** to Form **M3** ^a^

a The reaction was run for 20 h in DCM solution at room temperature. See the Experimental Section for additional details.

**Scheme 2:**
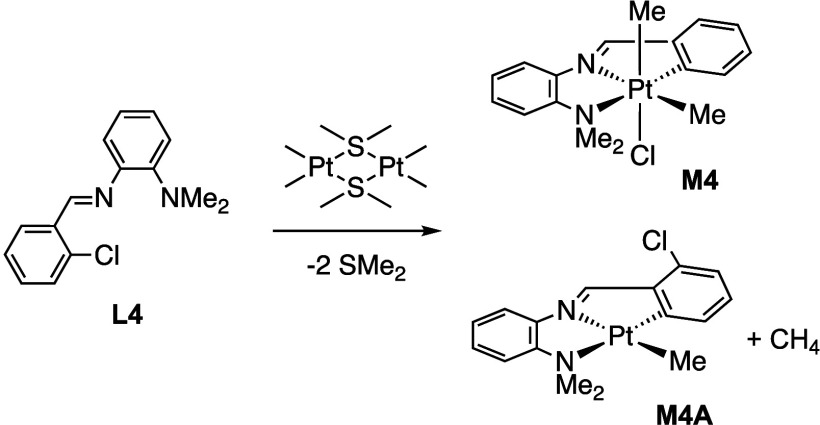
Reaction of **L4** to Form **M4** and **M4A** ^a^

a The reaction was run for 25 h in DCM solution at room temperature. See the Experimental Section for additional details.

The bromo compound formed by oxidative addition of the carbon–bromine bond resulted in an OC-6, six-coordinate octahedral species with the anionic N^N^C pincer ligand having all three of its donor atoms in a planar *mer* configuration. The bromide ligand and two methyl ligands fill out the coordination sphere with no anionic carbon donor atom being *trans* to another, thus in a *fac* configuration, which is expected of these strong-field carbon ligands.^38^ Figure 1 shows the ORTEP for the platinum­(IV) compound, **M3**. When the chloro analogue was utilized, a very different result was observed; both platinum­(II), **M4A**, and platinum­(IV), **M4**, species were observed in the reaction mixture (Figures 2 and 3, respectively). Two unique platinum compounds could be clearly identified from the NMR spectra of the mixture of the chloro species. The bond lengths and bond angles determined by SCXRD for **M3**, **M4**, and **M4A** are similar to those of previously reported cyclometalated Pt­(II) and Pt­(IV) compounds.^13,34,38–40^ By utilizing the symmetry of the molecules and their ^195^Pt coupling constants, given that platinum­(IV) compounds have smaller coupling constants than their platinum­(II) analogs, the compounds were identified.^41^

**Figure 1:**
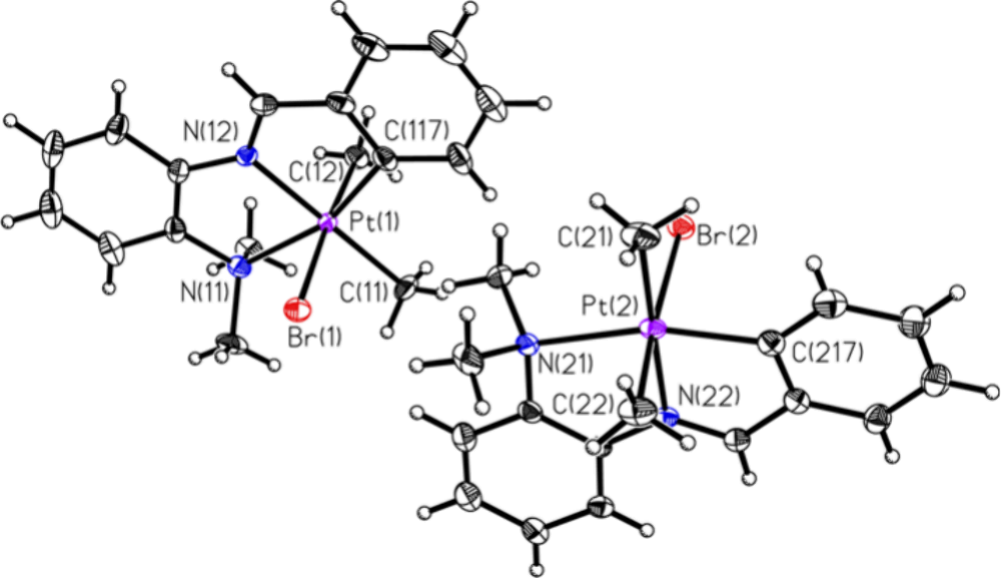
ORTEP of compound **M3** (50% probability of thermal ellipsoids). Selected bond lengths (Å) and angles (deg): Pt1–C11: 2.055 (3); Pt(1)–Br (1): 2.5927 (4); Pt(2)–Br (2): 2.6037 (4); Pt1–N12: 2.055 (3); Pt1–C12: 2.063 (3); Pt1–C117: 2.002 (3); Pt1–N11: 2.253 (2); Pt2–C217: 1.999 (3); Pt2–C21: 2.050 (3); Pt2–N22: 2.052 (2); Pt2–C22: 2.068; Pt2–N21: 2.262 (2); C117–Pt1–C11: 99.0 (1); C117–Pt1–N12: 81.2 (1); C11–Pt1–N12: 179.7 (2); C11–Pt1–C12: 86.9 (1); C117–Pt1–C12: 86.3 (1); N12–Pt1–C12: 92.9 (1); C117–Pt1–N11: 161.6 (1); C11–Pt1–N11: 99.4 (1); N12–Pt1–N11: 80.4 (9); C12–Pt1–Br1: 175.81 (9); C11–Pt1–Br1: 91.2 (1); C217–Pt2–C21: 97.2 (1); C217–Pt2–N22: 81.5 (1); C21–Pt2–N22: 177.8 (1); C21–Pt2–C22: 87.3 (1); C217–Pt2–C22: 88.5 (1); N22–Pt2–C22: 90.8 (1); C217–Pt2–N21: 161.7 (1); C21–Pt2–N21: 101.1 (1); N22–Pt2–N21: 80.2 (1); C22–Pt2–N21: 91.3 (1); C217–Pt2–Br2: 87.48 (8); C21–Pt2–Br2: 89.54 (9); C22–Pt2–Br2: 174.5 (1); N21–Pt2–Br2: 93.76 (6); N22–Pt2–Br2: 92.25 (6).

**Figure 2:**
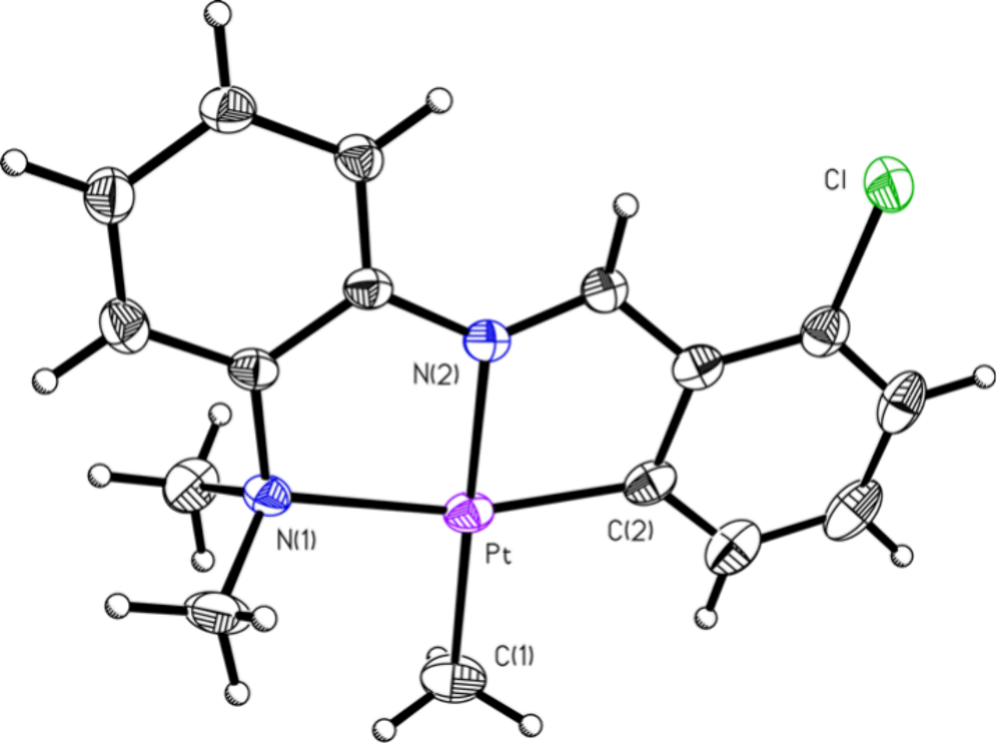
ORTEP of compound **M4A** (50% probability of thermal ellipsoids). Selected bond lengths (Å) and angles (deg): Pt–C2: 1.9843 (4); Pt–N2: 2.014 (3); Pt–C1: 2.075 (4); Pt–N1: 2.169; C2–Pt–N2: 81.9 (1); C2–Pt–C1: 97.6 (2); N2–Pt–C1: 179.1 (2); C2–Pt–N1: 164.0 (1); N2–Pt–N1: 82.1 (1); C1–Pt–N1: 98.4 (2).

**Figure 3:**
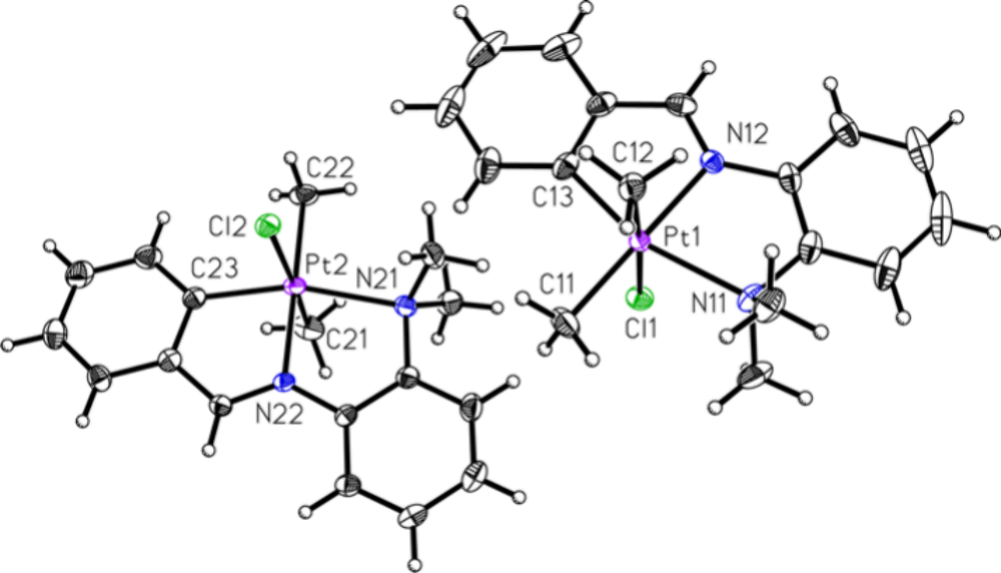
ORTEP of compound **M4**. Selected bond lengths (Å) and angles (deg): Pt1–C11: 2.056 (3); Pt1–C12: 2.061 (2); Pt1–N12: 2.052 (2); Pt1–C13: 1.998 (3); Pt1–N11: 2.249 (2); Pt1–Cl1: 2.4751 (6); Pt2–C21: 2.062 (3); Pt2–C22: 2.050 (3); Pt2–N22: 2.056 (2); Pt2–C23: 2.000 (3); Pt2–N21: 2.265 (2); Pt2–Cl2: 2.4754 (6); C13–Pt1–N12: 81.2 (1); C13–Pt1–C11: 99.0 (1); N12–Pt1–C11: 179.6 (1); C13–Pt1–C12: 86.7 (1); N12–Pt1–C12: 92.18 (9); C11–Pt1–C12: 87.5 (1); C13–Pt1–N11: 161.7 (1); N12–Pt1–N11: 80.58 (8); C11–Pt1–N11: 99.3 (1); C12–Pt1–N11: 92.42 (9); C13–Pt1–Cl1: 90.33 (7); N12–Pt1–Cl1: 89.39 (6); C11–Pt1–Cl1: 90.97 (8); C12–Pt1–Cl1: 176.41 (8); N11–Pt1–Cl1: 91.02 (6); C23–Pt2–C22: 98.0 (1); C23–Pt–N22: 81.28 (9); C22–Pt2–N22: 178.4 (1); C23–Pt2–C21: 88.6 (1); C22–Pt2–C21; 87.1 (1); N22–Pt2–C21: 91.5 (1); C23–Pt2–N21: 161.57 (9); C22–Pt2–N21: 100.4 (1); N22–Pt2–N21: 80.29 (8); C21–Pt2–N21: 91.5 (1); C23–Pt2–Cl2: 87.88 (7); C22–Pt2–Cl2: 89.96 (8); N22–Pt2–Cl2: 91.39 (6); C21–Pt2–Cl2: 175.03 (9); N21–Pt2–Cl2: 92.99 (6).

As mentioned above, the NMR spectra were extremely useful in the characterization of the compounds. For example, in the case of the platinum­(IV) bromo derivative (Figure 4), **M3**, two methyl platinum resonances were observed, two *N*-methyl resonances for the coordinated amine were observed, and a single imine resonance was observed, giving five diagnostic proton NMR peaks. All five resonances had ^195^Pt satellites with coupling constants appropriate for cyclometalated platinum­(IV)/methyl species.^42,43^ The ^13^C spectrum gave similar results corroborating the structure of **M3** as determined by SCXRD. Additionally, given the low symmetry at the platinum center, the *N*-methyl resonances are rendered inequivalent in the six-coordinate species. In the case of the chloro derivative, forming both **M4** and **M4A**, the mixture of these two species and their associated resonances were clearly observed in their NMR spectra. **M4** is formed similarly to **M3**, while **M4A** is suspected to be formed by orthometalation/C–H oxidative addition, followed by reductive elimination of methane to form the platinum­(II) species. Similar to bromo species **M3**, the NMR characterization of **M4** were centered on the analogous five diagnostic peaks of **M3**, whereas the proton NMR spectrum of **M4A** has only one platinum methyl resonance, one *N*-methyl resonance (the *N*-methyls are equivalent in the planar platinum­(II) species), and one imine resonance for a total of three diagnostic resonances. As mentioned, the platinum­(II) species has larger coupling ^195^Pt constants than the platinum­(IV) species and were easily identified in the mixture.^42,43^ Similarly consistent results were observed for the ^13^C NMR spectrum of the mixture of **M4** and **M4A**. When a mixture of **M4** and **M4A** was set for crystallization, a mixture of crystals of the two compounds was obtained, and the mixture could be observed under a microscope. Two different crystals were plucked from the mixture, one red crystal for the platinum­(II) species and one yellowish-orange crystal for the platinum­(IV) species, in order to obtain SCXRD results for both compounds, **M4A** and **M4** (Figures 2 and 3, respectively). A number of attempts were made to separate the mixture but were unsuccessful. Only after scouring mixtures of powders were we fortunate enough to obtain small crystals that were suitable for structural determination, however; we were not able to succeed at bulk purification in an appreciable yield of either product.

**Figure 4:**
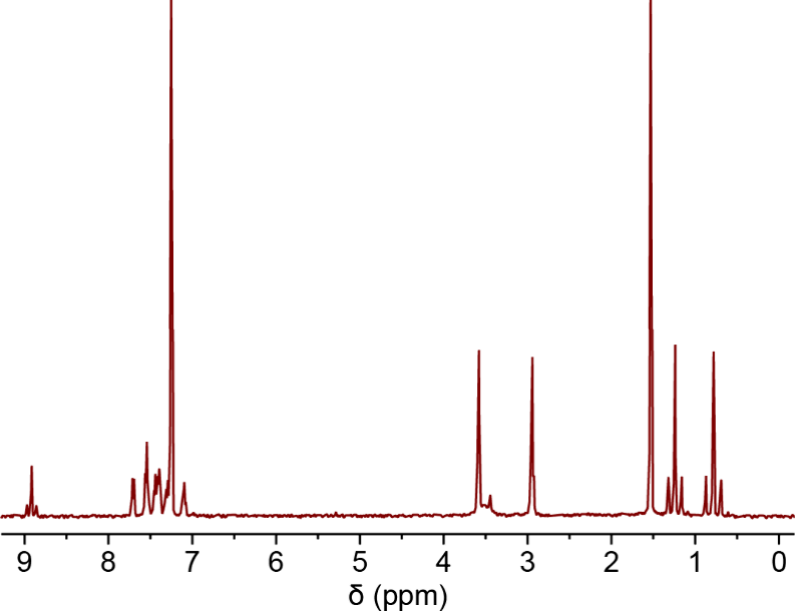
^1^H (400 MHz) NMR spectrum of **M3** (HOD at 1.56 ppm; CHCl_3_ at 7.26 ppm). See the Experimental Section for a detailed listing of resonances.

The reaction progress for both ligands **L3** and **L4** with platinum compound **1** was explored and monitored by proton NMR spectroscopy. The rate of appearance of the products’ proton NMR resonances was examined for the diagnostic peaks mentioned above. The resonances were integrated systematically, as the spectra were measured at set time intervals and plotted as integration/concentration vs time graphs. The resonances associated with compound **1** and ligands were observed to decrease as the product peaks increased as the reaction progressed over time. It is well accepted that the first steps toward cyclometalated product formation, via C–X/C–H bond activation, for reactions with **1** and multidentate N^N ligands involve first the dissociation of dms from the platinum starting material and subsequent coordination of the N^N ligand in question. Once a PtMe_2_(N^N) intermediate has formed, studies have shown that the intramolecular bond activation step follows first-order kinetics.^13,18,44^ In the case of the chloro derivative, where two products were observed and the reaction was slower than for the bromo analog (see below), the products’ resonances were integrated and fit to the first-order expression 1 – e^–*kt*^. This experimental value *k* represents the sum of the rate constants for the formation of platinum­(II) and platinum­(IV) products from a common reactant. Additionally, in this case, the rate constants for both platinum­(IV) and platinum­(II) products are quite similar (Tables S1 and S3) and within 10% at 298 K given the product ratio as measured by NMR (see below). Figure 5 shows an excerpt from the set of spectra for the N-Me_2_ region of a mixture of **L4** and **1**. The peak at 3.25 ppm is associated with the dimethyl amino protons of the platinum­(II) species, **M4A**, while the peak at 3.45 ppm is for one of the inequivalent methyl groups of **M4** and integrates to approximately one-half. Data were collected at several temperatures in the range of 298–332 K, an Eyring plot was generated, and the activation parameters were determined. The activation parameters, summarized in Table S4, are reasonably comparable to the literature for intramolecular bond activation of C–X/C–H bonds to platinum­(II).^13,44–46^ These parameters are also reasonably comparable to those of our DFT calculations (vide infra). The bromo analogue, having formed only one product, was straightforward to measure and analyze. The appearance of the product of oxidative addition of C–Br was found to fit the first-order expression 1 – e^–*kt*^ at 298 K. At temperatures above 298 K, the appearance of products seems to follow more complicated kinetics.

**Figure 5:**
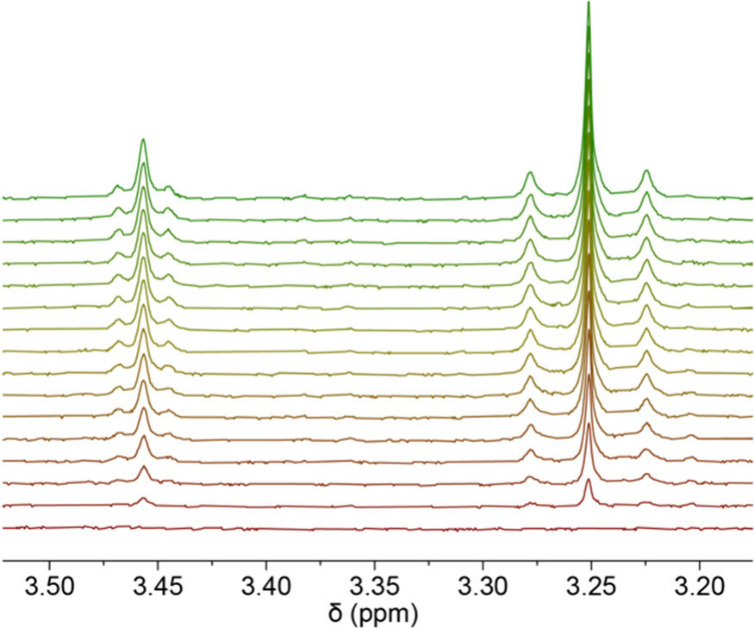
Spectra were taken at 900 s intervals for the reaction of **L4** with compound **1**. The peak growing at 3.45 ppm is one inequivalent N-Me on **M4**, while that at 3.24 ppm is the N-Me_2_ on **M4A**. The ratio of compound **M4**:**M4A** is 1:1.1 at 298 K in the NMR tube experiment.

### DFT Calculations

Density functional theory (DFT) and time-dependent density functional theory (TDDFT) calculations were run on all three platinum compounds, **M3**, **M4**, and **M4A**. The DFT results for bond lengths and bond angles show very good agreement with the SCXRD results for the three compounds characterized crystallographically (Table S9). To rationalize the different reactivities of **L3** and **L4** with compound **1**, we performed nudged elastic band (NEB) calculations to identify the C–X/C–H oxidative addition barriers. As is generally accepted, oxidative addition to platinum­(II) occurs from a three-coordinate species to give a five-coordinate species upon addition.^13,47^ We considered the oxidative addition reaction starting from a putative SP-4 platinum­(II) (PtMe_2_(N^N)), which, as mentioned above, is accepted to be the germane intermediate, which must preliminarily dissociate a labile ligand, which in our study is the dimethyl amino moiety.^13,18,44^ The five-coordinate species formed upon oxidative addition then has the dimethyl amino ligand reassociate to give the final OC-6 coordination mode. When we attempted to use four-coordinate species as starting points, the initial step was found to be the dissociation of the dimethylamino group. While many potential pathways are possible, we examined the pathway mentioned above to evaluate the relative barriers for bond activation. NEB calculations were performed to identify the saddle point between a three-coordinate T-shaped intermediate and a five-coordinate intermediate (Figure 6) for the three oxidative addition pathways discussed above (C–H, C–Cl, and C–Br). Additional molecular structures for all species along the reaction pathway for C–Cl and C–H are shown in Figure S18. Each optimized transition state shows exactly one negative frequency that corresponds to the reaction coordinate. The C–Br/C–Cl oxidative addition transition state is consistent with a concerted three-membered transition state,^48^ whereas for C–H activation, the saddle point resembles a platinum hydride between a T-shaped intermediate with an agnostic ortho *sp* ^*2*^ C–H (poised for activation) and a different T-shaped intermediate with an agnostic *sp* ^*3*^ C–H of a coordinated methane. A previous study has identified a similar reaction pathway for C–H oxidative addition.^49^ The activation energy for C–Br oxidative addition was lower than that for the C–Cl in the chloro derivative; however, the activation energy for C–Cl oxidative addition was almost identical to that for C–H bond activation for the formation of **M4** and **M4A**, respectively. These computational results are consistent with our experimental results giving competitive C–Cl and C–H activation and a lower barrier for C–Br activation. As can be read from Figure 6, the ΔG­(298 K)^⧧^ barriers for C–H, C–Cl, and C–Br activation, from intermediate **I**, are 86.4, 85.1, and 73.6 kJ/mol, respectively. The experimental values for C–H and C–Cl were determined to be 98.0 ± 8.4 and 98.1 ± 8.4%, respectively (Table S4).

**Figure 6:**
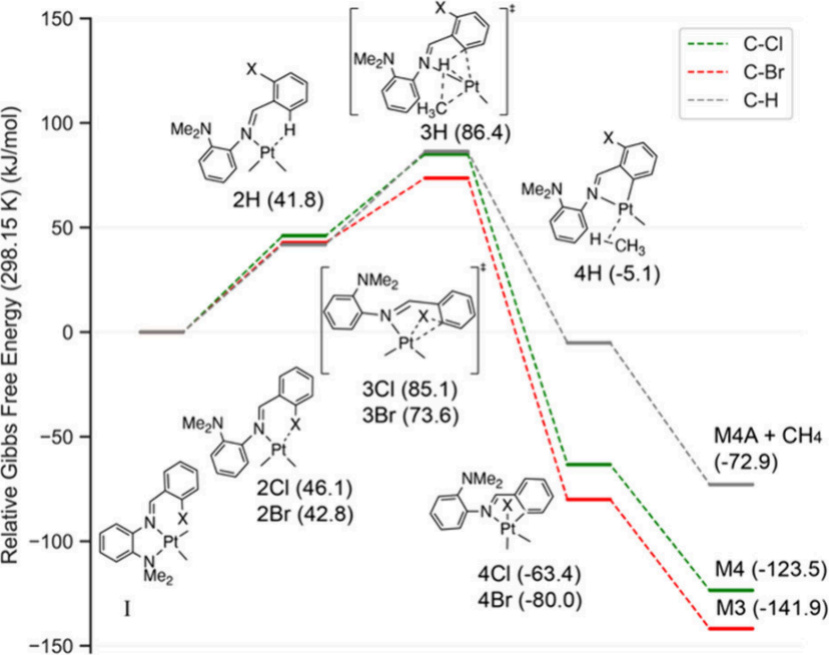
Gibbs free energy profile for the reaction pathways: C–Cl, C–H, and C–Br activation.

#### Photophysical Properties and TDDFT

The absorption (UV/vis) spectrum of the platinum bromo derivative, **M3**, was measured in dichloromethane (DCM) solution (Figure 7). The lowest-energy band, centered at around 400 nm, has an extinction coefficient of 5700 cm^–1^ M^–1^ and is tentatively assigned to possess some MLCT character. The higher-energy band (∼330 nm) in the UV region was slightly more intense. Both of these features were reproduced in our TDDFT simulated absorbance spectrum (Figure 8). Two high oscillator strength transitions are observed, matching the two experimental bands. NTO analysis indicates that these transitions have MLCT/ILCT character (Figures S14 and S15). The HOMO and near HOMO (Figure 9) show electronic density on the π system of the ligand, metal *d* orbitals, and bromo *p* orbitals. The LUMO is primarily of π* character (Table S9). For each band, there exists splitting into two peaks, which we tentatively assign as vibronic features. These vibronic features can be reproduced using excited-state dynamics calculations (Figure S16).

**Figure 7:**
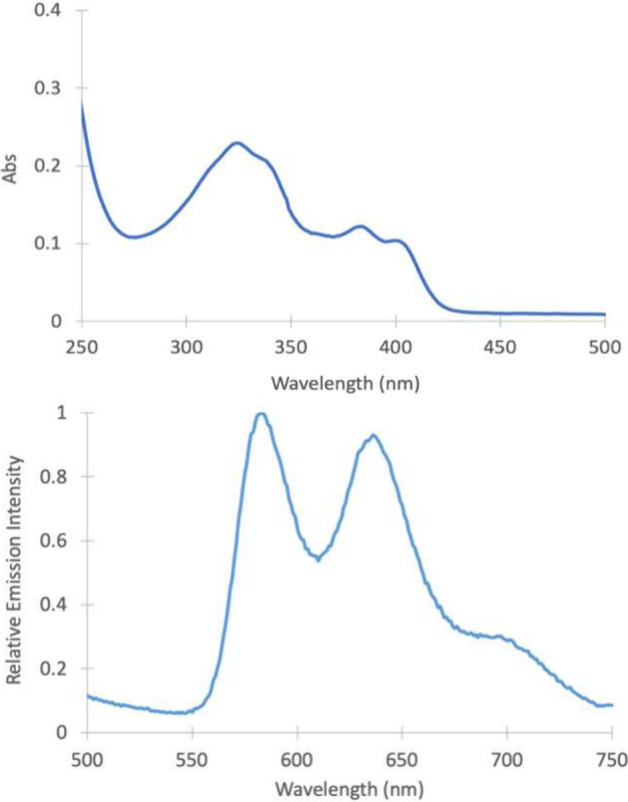
UV/vis absorbance spectrum (top) and steady-state emission spectrum (in degassed DCM) of **M3** excited at 400 nm (bottom).

**Figure 8:**
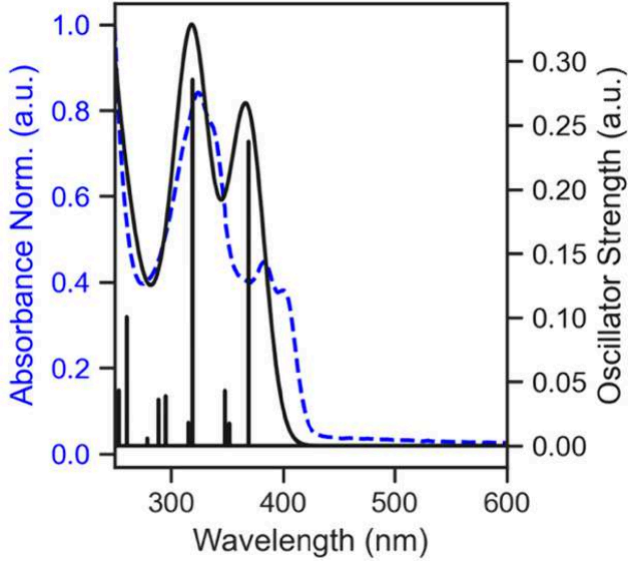
Normalized absorbance (dashed blue) and TDDFT calculated (solid black) for **M3**.

**Figure 9:**
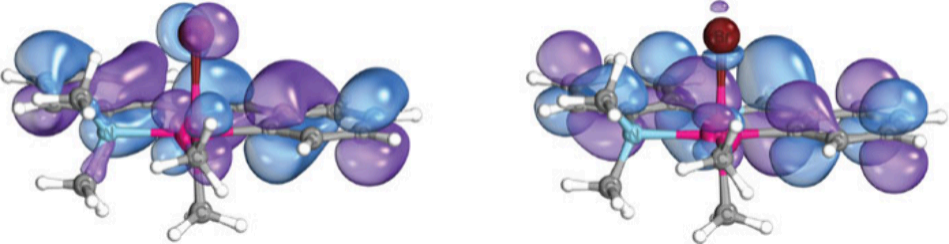
DFT calculated HOMO (left) and LUMO (right) of **M3** plotted at the 80% isosurface threshold using IboView.

The emission spectrum was also measured for compound **M3** (in degassed DCM), and multiple emission bands were observed with the two largest bands appearing at 585 and 635 nm. The excited-state lifetime was measured using time-correlated single photon counting (TCSPC) at 585 and 635 nm and was found to be 871 and 876 ns, respectively, characteristic of phosphorescence from a triplet state following intersystem crossing (ISC), which is well known for platinum compounds.^50–52^ Additionally, excited-state dynamics simulations of the phosphorescence spectrum for **M3** exhibits multiples bands similar to the experimental spectrum, corroborating the phosphorescence assignment (Figure S17). The quantum yield was found to be quite modest, at around 0.1% (Figure S13), for **M3**.

Femtosecond transient absorption spectroscopy (fs-TA) measurements were taken of **M3** in degassed DCM to provide insight into the excited-state formation and decay dynamics (Figure 10). A three-component global fit of the transient absorption surface was performed and was fit well using two time constants. A short-lived excited-state absorption was observed at 450 nm, having a lifetime of 24.4 ps. Given the short-lived lifetime, we speculate a singlet state. This peak overlapped with a long-lived excited-state absorption at 430 nm, which was stable for the duration of the 7 ns time window of the experiment and was fit to an infinite lifetime. Given the long lifetime, we speculate this to be a triplet state. No ground-state bleaching was observed, perhaps because it was masked by excited-state absorption. Similar broad positive absorption across the visible spectrum has been reported previously for cyclometalated platinum compounds.^53^

**Figure 10:**
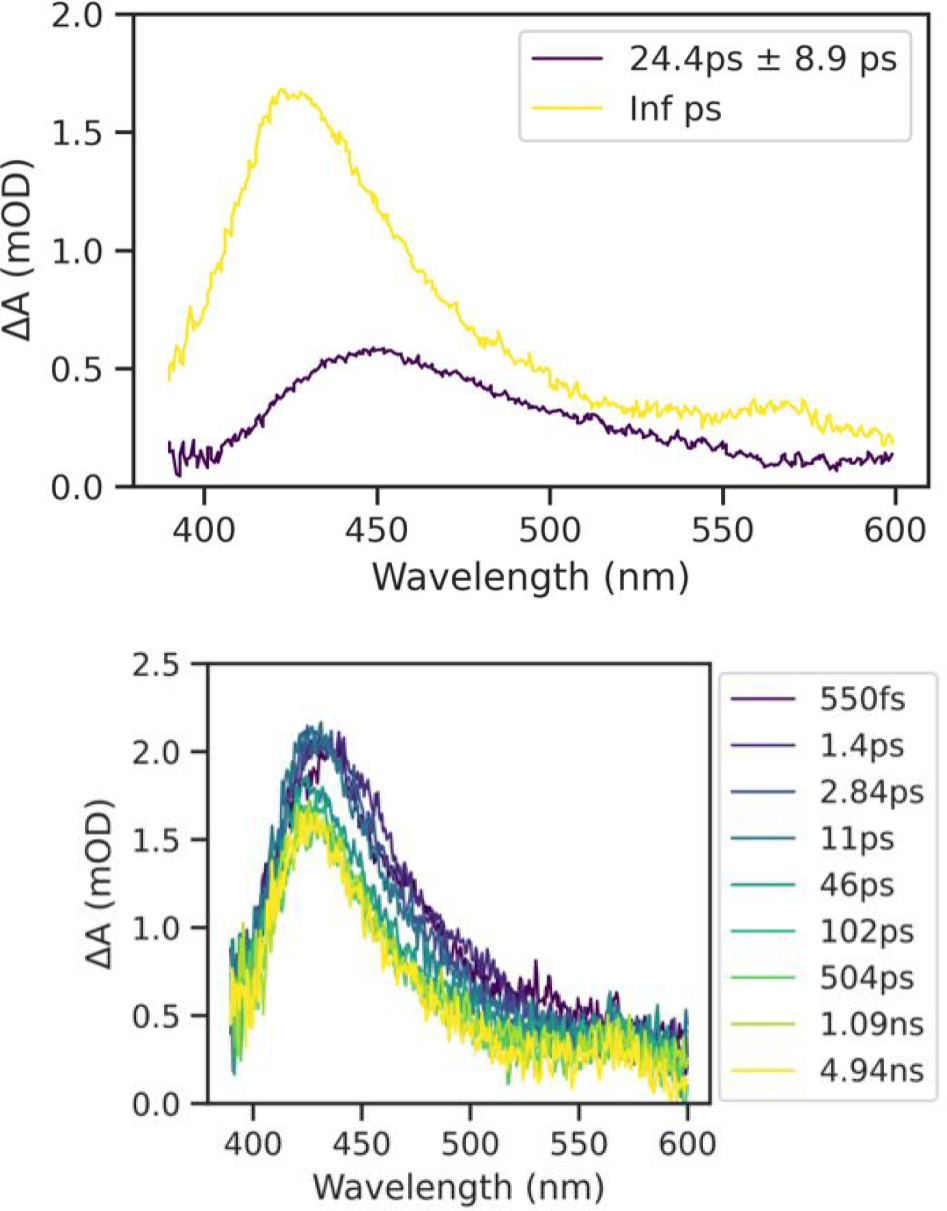
Top: Decay-associated difference spectra (DADS) for **M3** (in degassed DCM). Bottom: Representative set of spectra for the fs-TA of compound **3M** obtained for time delays of 100 fs–7 ns.

#### Summary

The platinum­(IV) cyclometalated compound **M3** was synthesized and characterized and exhibited room-temperature emission in DCM solution with multiple bands, which may be of interest as emission spectroscopy for platinum­(IV) species is much less reported than that for platinum­(II) species. Additionally, given the multiple emission bands, this compound may contribute to the understanding of dual-emissive compounds and aid in the development of devices that utilize ratiometric analysis. For the chloro ligand analog, C–H activation was found to compete with C–Cl oxidative addition, but C–H activation did not compete effectively with C–Br oxidative addition for the N^N^C anionic pincer ligands studied. C–Cl is known to be much more reactive than C–H activation, and this is usually attributed to the lower bond energy of the C–Cl bond compared to that of the C–H bond. As mentioned earlier, a couple of examples of anionic N^C chelate ligands that contain two chlorides on the benzene ring have been reported that show this anomalous behavior. In those cases, the reactivity was rationalized by suggesting that the extra chloride’s electron-withdrawing nature enhanced the C–H reactivity relative to the C–Cl reactivity. In the case of **L4**, reported here, we achieved this result without an additional chloride using a flat tridentate anionic (N^N^C) ligand.

## Experimental Section

### General

Solvents and reagents were purchased from Sigma-Aldrich and used as received, unless otherwise noted. K_2_PtCl_4_ was purchased from J. and J. Materials (NJ). NMR spectra were recorded at Bard College using a Varian NMR-400 MHz spectrometer (^1^H, 400 MHz; ^13^C, 100.6 MHz) and a Magritek 80 MHz spectrometer. Shifts are given in ppm, and coupling constant *J* values are given in Hz. Abbreviations used: s = singlet; d = doublet; t = triplet; and m = multiplet. Mass spectrometry data was collected at Boston College. IR spectra were recorded with a Thermo instrument with an ATR attachment.

### Computational Details

ORCA *ab initio* quantum chemistry program version 5.0.4 ^54,55^ was used for TDDFT. All calculations were performed using the PBE0 ^56,57^ functional and D3­(BJ) dispersion correction.^58^ The ZORA^59^ scalar relativistic approximation was used with ZORA-def2-TZVP^60^ basis sets for Cl, Br, N, C, and H atoms, and the SARC-ZORA-TZVP^61^ basis set was used for Pt, with SARC/J decontracted and def2/J^62^ and SARC^63^ auxiliary basis sets. Optimized structures were confirmed to be at an energetic minimum by the absence of imaginary frequencies or the presence of a single negative frequency for optimized transition states. Nudged elastic band calculations^64,65^ were used to identify transition states using 14 images along the reaction path with a conductor-like polarizable continuum model (CPCM) for solvation effects.^66^ Thermochemistry is calculated at 298.15 K and 1 atm pressure with multiplicity S = 1. UV/vis simulation TDDFT calculations of the singlet excitations were performed without the use of the Tamm-Dancoff approximation using 30 singlet excitations with CPCM for solvation effects.^66^ Excited-state dynamics module calculations for phosphorescence and vibronic absorption spectra^67,68^ simulations were performed using the vertical gradient approximation for excited-state Hessians and CPCM solvation at the TDDFT level. In phosphorescence calculations, RI-SOMF­(1X) was used to accelerate the spin–orbit coupling integrals.^69^

### Photophysical Measurements

Steady-state emission spectra were recorded using a PTI QM-40 instrument with a PMT detector, which is sensitive up to 850 nm. In these experiments, the concentration of the platinum complexes ranged from 10 to 20 μM in degassed solvents. The fluorimeter emission spectrum was corrected using a method described in the literature which uses four standard fluorophores to calibrate the response of the instrument.^70^ The slits were set at 2.5 nm bandpass for all solution measurements. The luminescence lifetimes of the complexes were measured by time-correlated single-photon counting (TCSPC) following excitation with a 405 nm LED. For TCSPC measurements, the slits were adjusted such that <3% of the LED flashes resulted in a detection event ensuring such events are single photons. Solution samples were degassed for 5 min prior to measurement. Relative quantum yield (QY) measurements were taken by first measuring the absorbance of **M3** solutions with a Cary 100 UV/vis spectrometer and then recording their emission spectrum with an excitation of 400 nm using a PTI QM400 (Horiba) equipped with a 920C cooled PMT detector. The same was done for solutions of [Ru­(bpy)_3_]­Cl_2_ in water as a standard reference.^71–73^ Femtosecond transient absorption (fs-TA) spectroscopy was performed at Brookhaven National Laboratory using a Helios Fire (Ultrafast Systems) spectrometer that used a Spitfire SpectraPhysics regenerative amplifier and two TOPAS OPAs operating at a 1 kHz repetition rate. fs-TA data reduction and analysis was performed using Surface Xplorer 4.3.0 software (Ultrafast Systems). Raw data were background-subtracted and chirp-corrected. Singular value decomposition was used to determine the number of principal components. Multiexponential global analysis fitting was then performed to extract decay-associated difference spectra and their corresponding lifetimes.

### X-ray Diffraction

**M3**, **M4**, and **M4A** were crystallized by the slow diffusion of pentane into an acetone solution of each compound. X-ray diffraction data were collected on a Bruker APEX 2 CCD platform diffractometer (Mo Kα (l = 0.71073 Å)) at 125 K with crystals mounted in a nylon loop with Paratone-N cryo-protectant oil. The structures were solved using direct methods (SHELXT 2018/2)^74^ and standard difference map techniques and were refined by full-matrix least-squares procedures on F2 (SHELXL 2017/1).^75^ All non-hydrogen atoms were refined anisotropically.

### Kinetic NMR Experiments

Samples of dimer **1** and ligand were weighed into separate vials and dissolved in CDCl_3_. The two solutions were mixed together and stirred. This solution was then transferred to a J Young NMR tube and placed in the spectrometer. The sample was run every 10 minutes for approximately nine hours. For example, 7.2 mg of **1** was dissolved in 0.7 mL of CDCl_3_ and 6.5 mg (2 equiv) of **L4** was dissolved in 0.7 mL of CDCl_3_, and then 0.3 mL from each was transferred to a third vial and mixed. This final mixture was transferred to the J Young NMR tube, the tube was placed in a spectrometer, and NMR data collection was started. The temperature was set to the desired temperature for each run at 298, 306, 314, or 322 K.

### Preparation of Compounds

See the SI for additional experimental details, including copies of NMR spectra, UV/vis spectra, and emission spectra. [Pt_2_Me_4_(μ-SMe_2_)_2_] was prepared according to the literature.^12^ Ligands **L3** and **L4** were synthesized by simple condensation reactions.^45,76^

#### [C_15_H_15_BrN_2_]: **L3**

2-Bromobenzaldehyde (67.9 mg, 367.1 μmol) was dissolved in DCM and combined with 2-amino-*N*,*N*-dimethylaniline (50 mg, 367.1 μmol), at which point the solution was stirred for 4 h. The solvent was then removed by rotary evaporation, producing an amber semisolid product (69 mg, 62%) that was characterized by ^1^H NMR spectroscopy. ^1^H NMR (400 MHz, CDCl_3_): δ = 2.88 (s, 6H, N–CH_3_), 6.98–7.61 (aromatic), 8.30 (d, 1H, H–C), 8.84 (s, 1H, CH = N) ppm.

**L4** was prepared similarly, rendering an amber semisolid product. Yield: 54%. Appearance: Amber, semisolid.

#### [C_15_H_15_ClN_2_]: **L4**

^1^H NMR (400 MHz, CDCl_3_): δ = 2.88 (s, 6H, N–CH_3_), 6.73–7.42 (aromatic), 8.34 (d, 1H, H–C), 8.92 (s, 1H, CH = N) ppm.

#### [C_17_H_21_BrN_2_Pt], **M3**

**L3** (0.042g, 138 μmol) and the platinum dimer [Pt_2_Me_4_(μ-SMe_2_)_2_] (0.039g, 69 μmol) were dissolved in dichloromethane, and the resulting solution was stirred for 20 h, at which point the solvent was removed with a rotary evaporator. The remaining product was triturated with pentane and recrystallized using DCM/pentane in a vial-in-a-vial diffusion. The orange crystals were then characterized by ^1^H NMR (NMR = ion ring) spectroscopy. Yield 76%. ^1^H NMR (400 MHz, CDCl_3_): δ = 0.79 (s, 3H, ^2^ *J*(PtH) = 70 Hz, MePt), 1.25 (s, 3H, ^2^ *J*(PtH) = 60 Hz, MePt), 2.96 (s, 3H, ^3^ *J*(PtH) = 14 Hz, N–CH_3_), 3.59 (s, 3H, ^3^ *J*(PtH) = 8 Hz, N–CH_3_), 7.11–7.73 (aromatic), 8.93 (s, 1H, ^3^ *J*(PtH) = 44 Hz, Pt–CH = N) ppm. ^13^C NMR (100.6 MHz, CDCl_3_) δ = −3.5 (Pt–CH_3_) ^1^ *J*(Pt–C) = 642 Hz; 3.1 (Pt–CH_3_) ^1^ *J*(Pt–C) = 679 Hz; 52.6 (N–CH_3_); 55.1 (N–CH_3_); 118.2 *J*(Pt–C) = 10 Hz; 122.3; 124.4; 128.9; 130.8; 130.9 *J*(Pt–C) = 42 Hz; 131.7 *J*(Pt–C) = 33 Hz; 133.4 *J*(Pt–C) = 57 Hz; 139.6; 141.9; 146.1; 154.4; 160.2 ^2^ *J*(Pt–C) = 52 Hz. Mass spectrometry: *m*/*z* calculated for [C_17_H_21_BrN_2_Pt]^+^: 527.05304; found: 527.05284. Elemental analysis, % calculated for C_17_H_21_N_2_PtBr: C, 38.65; H, 4.01; N, 5.30; found: C, 37.98; H, 3.59; N, 4.78. M.P. 191–193 °C (decomp).

#### [C_17_H_21_ClN_2_Pt], **M4** and [C_16_H_17_ClN_2_Pt], **M4A**

**L4** (26 mg, 100.5 μmol) and the platinum dimer [Pt_2_Me_4_(μ-SMe_2_)_2_] (28.8 mg, 50.2 μmol) were dissolved in dichloromethane, and the resulting solution was stirred for 25 h, at which point the solvent was removed with a rotary evaporator. The remaining product was triturated with cold pentane. This yielded a mixture of the Pt­(IV) expected product, **M4**, and a Pt­(II) product, **M4A**. (Total mixture 19.2 mg, 79%.) ^1^H NMR (400 MHz, CDCl_3_): δ = 0.71 (s, 3H, ^2^ *J*(PtH) = 73 Hz, Pt­(IV)-Me), 1.10 (s, 3H, ^2^ *J*(PtH) = 78 Hz, Pt­(II)–Me), 1.18 (s, 3H, ^2^ *J*(PtH) = 62 Hz, Pt­(IV)–Me), 2.96 (s, 3H, ^3^ *J*(PtH) = 15 Hz, Pt­(IV)–N–CH_3_), 3.25 (s, 6H, ^3^ *J*(PtH) = 21 Hz, Pt­(II)–N–CH_3_), 3.46 (s, 3H, ^3^ *J*(PtH) = 8 Hz, Pt­(IV)–N–CH_3_), 6.94–7.69 {aromatic}, 8.94 (s, 1H, ^3^ *J*(PtH) = 44 Hz, Pt­(IV)–CHN), 9.58 (s, 1H, ^3^ *J*(PtH) = 58 Hz, Pt­(II)–CHN) ppm. ^13^C NMR (100.6 MHz, CDCl_3_): δ = −10.1 (Pt–CH_3_) ^1^ *J*(Pt­(II)–C) = 808 Hz; −2.6 (Pt–CH_3_) ^1^ *J*(Pt­(IV)–C) = 652 Hz; −1.92 (Pt–CH_3_) ^1^ *J*(Pt­(IV)–C) = 688 Hz; 51.9 (N–CH_3_) ^2^ *J*(Pt­(IV)–C) = 16 Hz; 52.3 (N–CH_3_) ^2^ *J*(Pt­(IV)–C) = 18 Hz; 53.4. (N–CH_3_) ^2^ *J*(Pt­(II)–C) = 32 Hz; 158.1 ^2^ *J*(Pt­(IV)–C) = 92 Hz; 160.2 ^2^ *J*(Pt­(II)–C) = 160 Hz. Mass spectrometry: *m*/*z* calculated for [C_15_H_15_N_2_Pt]^+^: 418.0878, found: 418.0811; *m*/*z* calculated for [C_16_H_18_N_2_Pt]^+^: 433.1112, found: 433.1082; *m*/*z* calculated for [C_16_H_17_N_2_PtCl]^+^: 467.0723, found: 467.0717.

## Supplementary Material









## Notes

NMR and IR spectra, emission spectral data, orbital plots, calculated bond lengths, and coordinates for computational results (PDF)
CIF of **M3** (CSD no. 2495162) (CIF)
CIF of **M4** (CSD no. 2495165) (CIF)
CIF of **M4A** (CSD no. 2495164) (CIF)
