# Supplementary material for: Cyclometalated Platinum Compounds from Competing C–H/C–X Bond Activation Pathways
Source: ACS Omega. 2026 Mar 3;11(10):16603–11. doi: 10.1021/acsomega.5c12884 (PMC13000562; doi:10.1021/acsomega.5c12884)
Supplement: Supplementary file 1 [file ao5c12884_si_001.pdf]

Supporting Information

Cyclometalated Platinum Compounds from Competing C-H/C-X Bond Activation Pathways

Craig M. Anderson,*[a] Matthew W. Greenberg,[a] Christopher N. LaFratta,[a] Monika Dziubelski,[a] Zainab Aleem,[a] Benett B. Hathaway,[a] Joseph M. Tanski,[b]

[a] Department of Chemistry & Biochemistry, Bard College, 30 Campus Road, Annandale-on-Hudson, NY, 12504, USA.

Email: canderso@bard.edu Phone: 845-752-2356. FAX: 845-752-2339

[b] Department of Chemistry, Vassar College, Poughkeepsie, NY, 12604, USA.

KEYWORDS: oxidative addition, DFT, TDDFT; cyclometalation, C-H activation, phosphorescence

Table of Contents

Synthesis and Characterization of Compounds	S2
Kinetic Data, Eyring Plots, and Activation Parameters	S12-S26
Photophysical Data	S26
DFT Orbital Plots	S28
DFT Calculated Bonds and Angles	S29-S31
DFT NTO Orbital Plots	S32
DFT ESD Spectra	S33
DFT Reaction Pathway 3D Structures	S34-S35
DFT Energies and Coordinates for Computational Results	S35-S50
DFT Transition State 3D Structures and Selected Distances	S50-S52
X-ray Diffraction parameters	S53-S56
Bond lengths and Angles for **M3**, **M4**, and **M4A**	S56-S64

L3

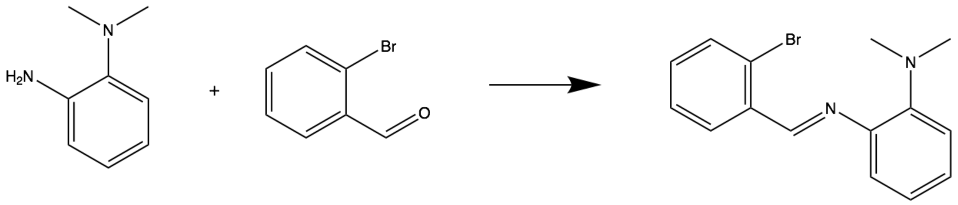

[$C_{15}H_{15}BrN_2$]: **L3**

2-bromobenzaldehyde (67.9 mg, 367.1μmol) was dissolved in DCM and combined with 2-amino-N, N-dimethylaniline (50 mg, 367.1μmol), at which point the solution was stirred for 4 hours. The solvent was then removed by rotary evaporation, and the product (69 mg, 62%) was characterized by ^{1}H-NMR spectroscopy. ^{1}H-NMR (400 MHz, $CDCl_3$) δ = 2.88 (s, 6H, N-CH_3), 6.98-7.61 (aromatic), 8.30 (d, 1H, H-C), 8.84 (s, 1H, CH=N) ppm.

^{1}H-NMR of L3.

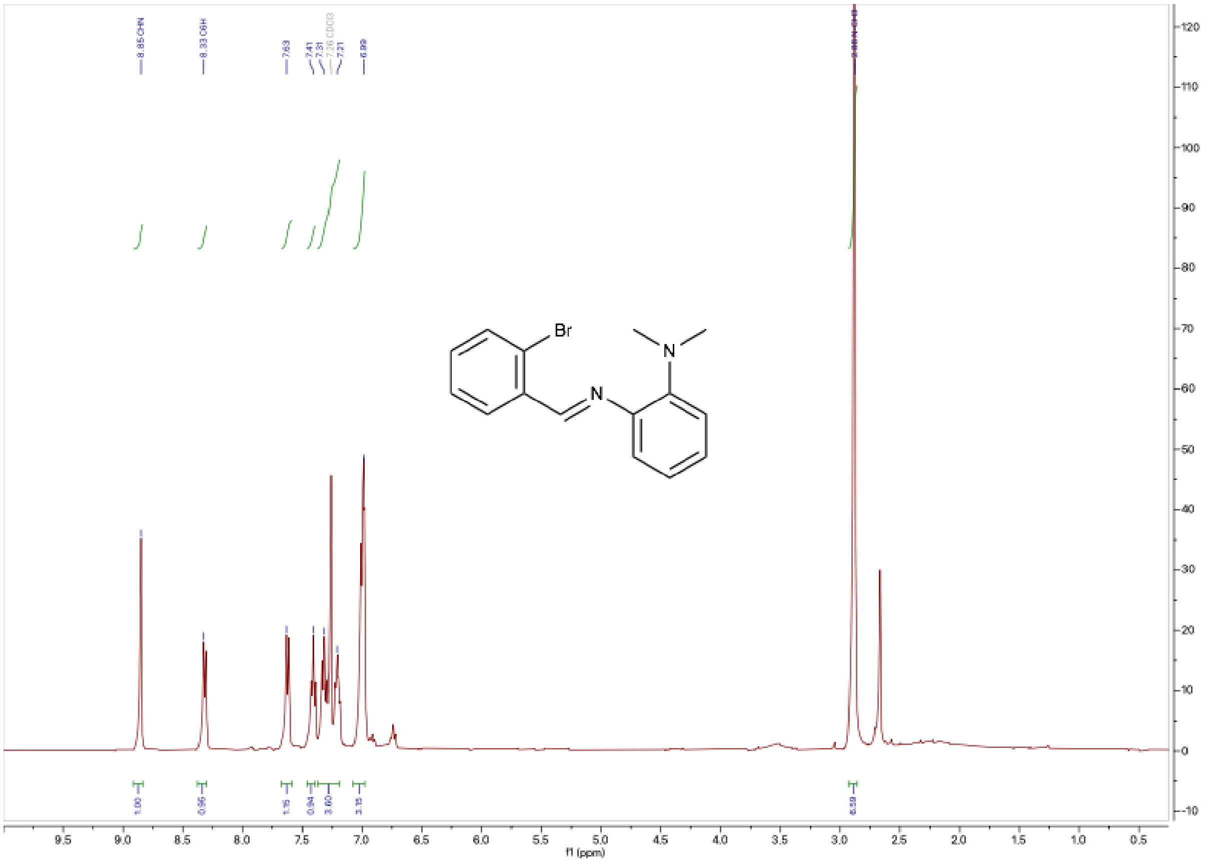

Figure S1: Proton NMR of **L3**

M3

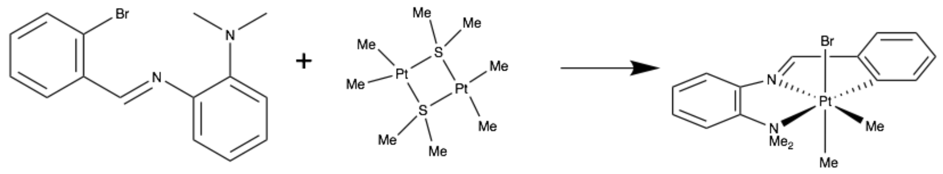

[$C_{17}H_{21}BrN_2Pt$], **M3**:

L3 (0.042g, 138 µmol) and the platinum dimer [$Pt_2Me_4(\mu\text{-}SMe_2)_2$] (0.039g, 69 µmol) were dissolved in dichloromethane, and the resulting solution was stirred for 20 hours, at which point the solvent was removed with a rotary evaporator, and the remaining product was triturated with pentane and recrystallized using DCM/pentane vial-in-a-vial diffusion. The orange crystals were then characterized by ^{1}H-NMR spectroscopy. ^{1}H-NMR (400 MHz, $CDCl_3$): 0.79 (s, 3H, ^2J(PtH) = 70, MePt), 1.25 (s, 3H, ^2J(PtH) = 60, MePt), 2.96 (s, 3H, ^3J(PtH) = 14, N-CH_3), 3.59 (s, 3H, ^3J(PtH) = 8, N-CH_3), 7.11-7.73 (aromatic), 8.93 (s, 1H, ^3J(PtH) = 44, Pt-CH=N) ppm. Mass spec: calculated for $C_{17}H_{21}BrN_2Pt$: 527.05304; found: 527.05284. ^{13}C NMR (100.6 MHz, $CDCl_3$) δ = -3.5 (CH_3) ^1J(Pt-C)= 642; 3.1 (CH_3) ^1J(Pt-C)= 679; 52.6 (N-CH_3); 55.1 (N-CH_3); 118.2 J(Pt-C)= 10; 122.3; 124.4; 128.9; 130.8; 130.9 J(Pt-C) = 42; 131.7 J(Pt-C)= 33; 133.4 J(Pt-C)= 57; 139.6; 141.9; 146.1; 154.4; 160.2 2J(Pt-C)= 52.

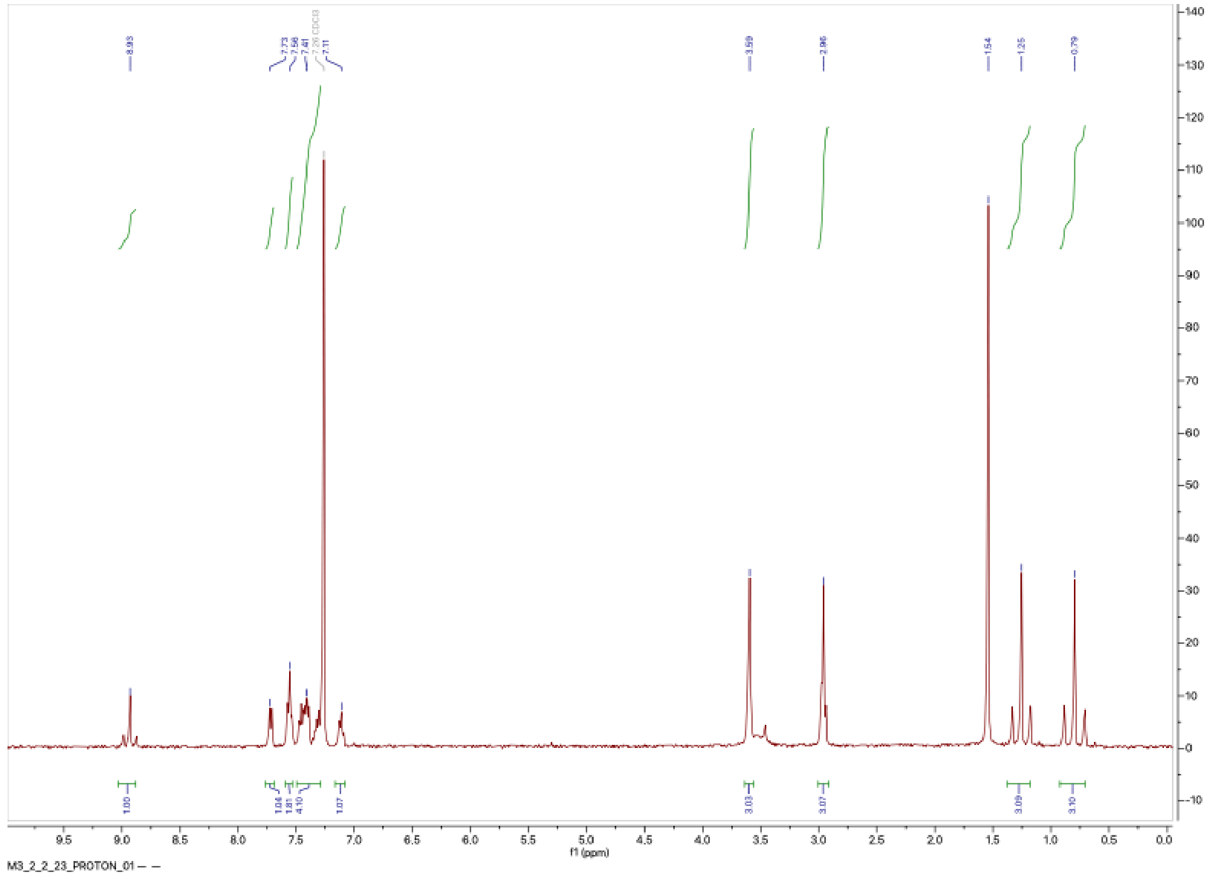

Figure S2: **^{1}H-NMR of M3.**

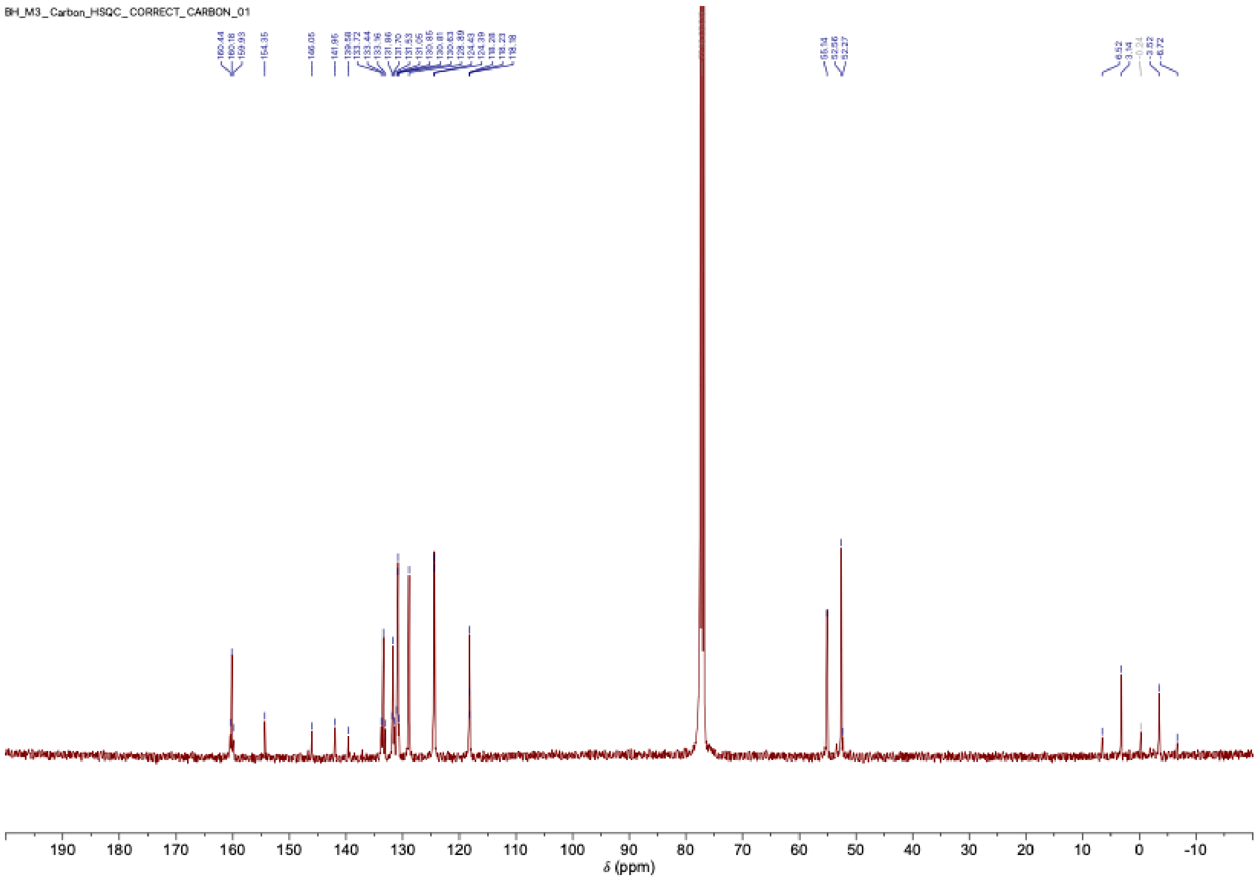

Figure S3: ^{13}C-NMR of M3.

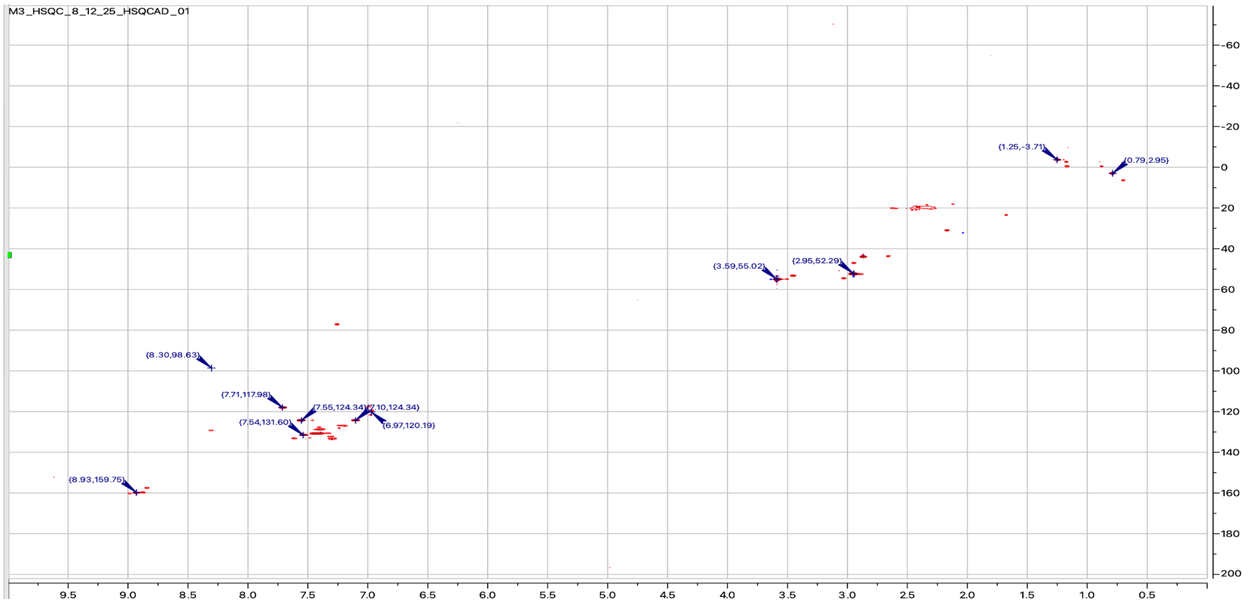

Figure S3A: HSQC with peak picking for **M3**.

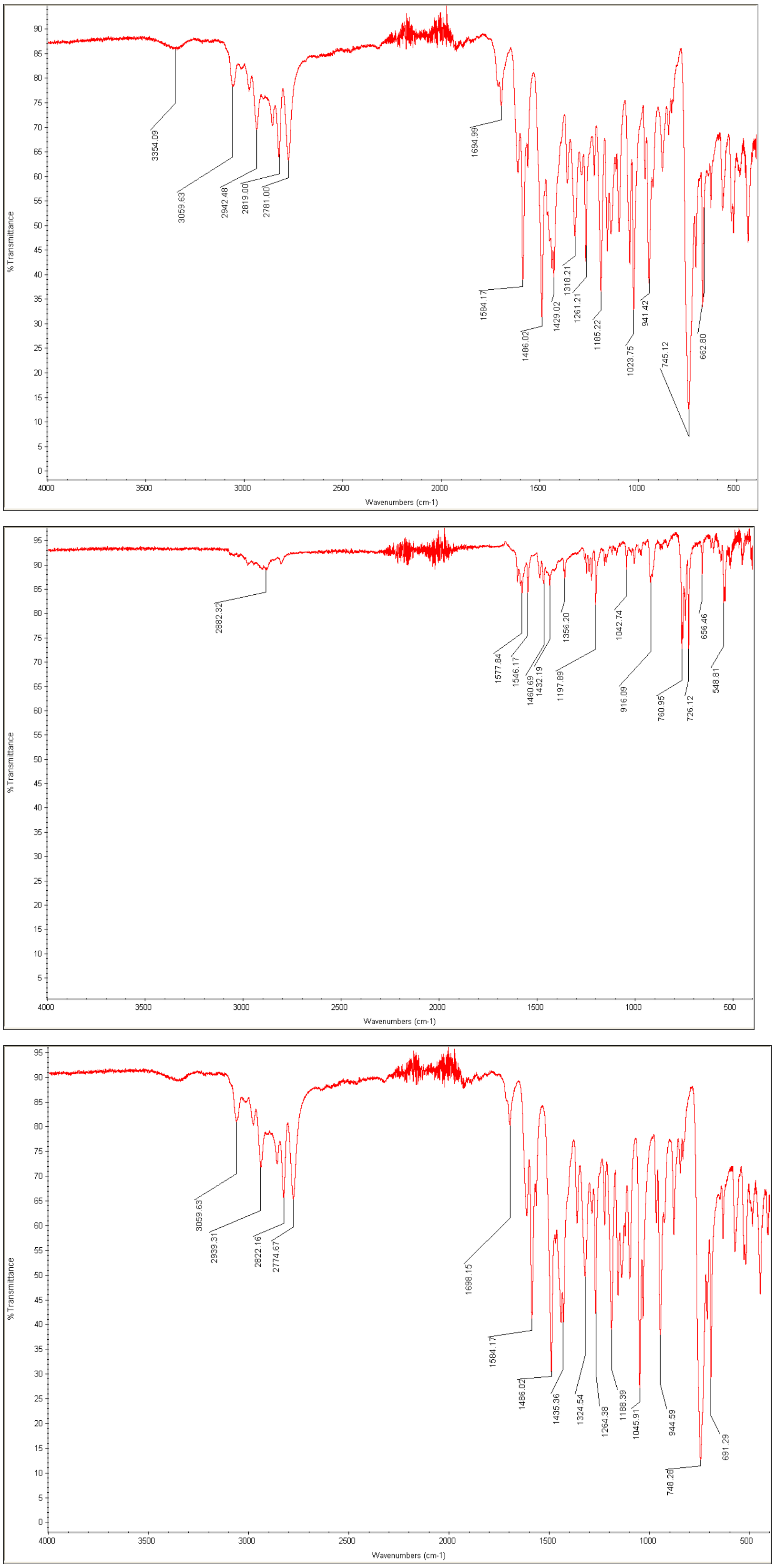

Figure S4: IR for **L3** (top), **M3 (**middle), and **L4** (bottom), respectively.

L4

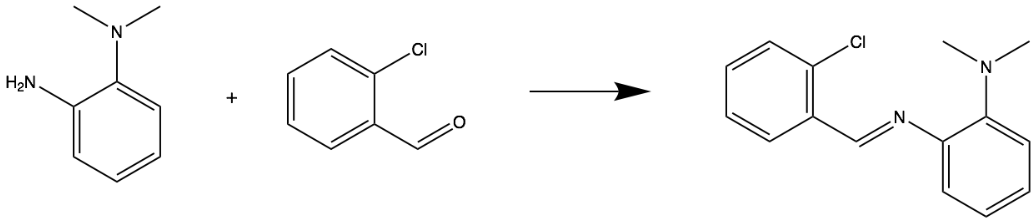

[$C_{15}H_{15}ClN_2$]

2-chlorobenzaldehyde (0.1579g, 1.12mmol) was dissolved in DCM and combined with 2-amino-N, N-dimethylaniline (0.1525g, 1.12mmol), at which point the solution was stirred for 10 hours. The solvent was then removed by rotary evaporation, and the product (0.204 g, 70.4%) was characterized by ^{1}H-NMR spectroscopy. ^{1}H-NMR (400 MHz, $CDCl_3$) δ = 2.88 (s, 6H, N-CH_3), 6.73-7.42 (aromatic), 8.34 (d, 1H, H-C), 8.92 (s, 1H, CH=N) ppm.

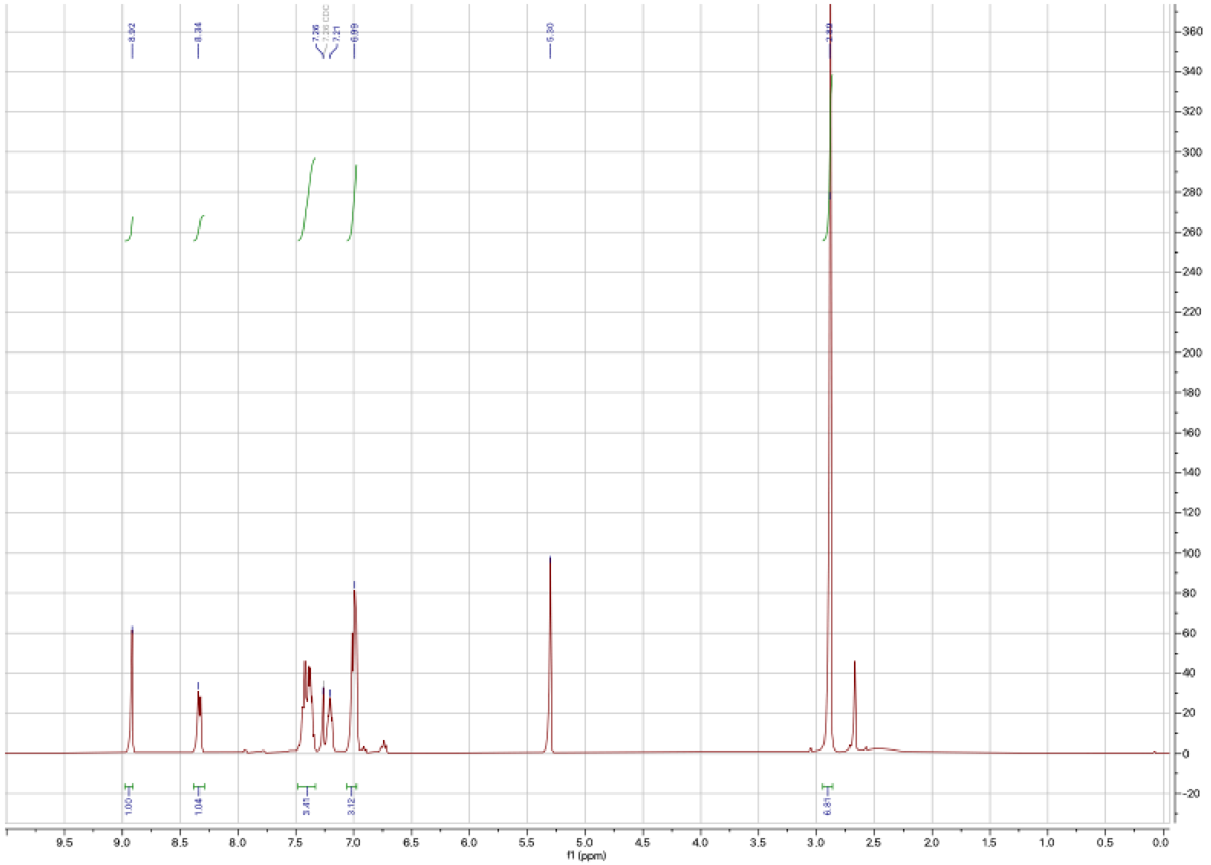

Figure **S5:** ^{1}H-NMR of **L4.**

Microwave Procedure for **L4**:

2-chlorobenzaldehyde (73.2mg, 0.52mmol) was dissolved in acetone and combined with 2-amino-N, N-dimethylaniline (71.5mg, 0.53mmol) and allowed to react in the microwave (5 minutes, 56°C, 250 PI, 200 W). The solvent was removed by rotary evaporation and the product (74.5mg, 54.11%) was characterized by ^{1}H-NMR spectroscopy. ^{1}H-NMR (400 MHz, $CDCl_3$) δ= 2.88 (s, 6H, N-CH_3), 6.74-7.44 (aromatic), 8.33 (d, 1H, H-C), 8.91(s, 1H, CH=N) ppm.

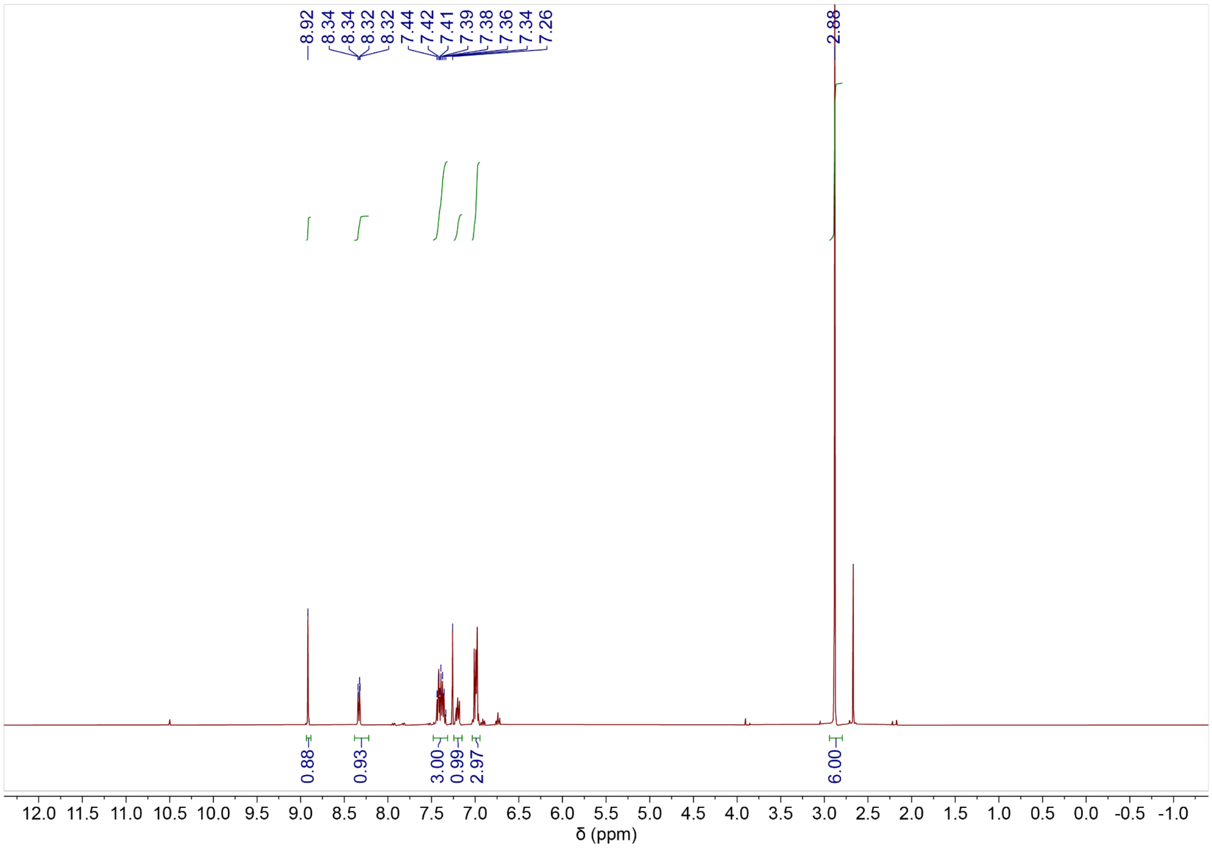

Figure S6: ^{1}H-NMR of **L4** for Microwave Reaction.

M4 and M4A

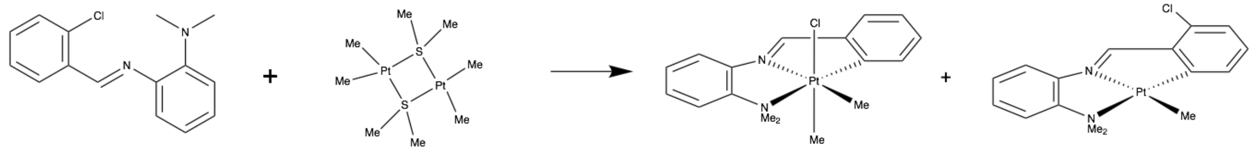

[$C_{17}H_{21}ClN_2Pt$], **M4** and [$C_{16}H_{17}ClN_2Pt$], **M4A**:
L4 (26 mg, 100.5 μmol) and the platinum dimer [$Pt_2Me_4(\mu\text{-}SMe_{2)2}$] (28.8 mg, 50.2 μmol) were dissolved in dichloromethane, and the resulting solution was stirred for 25 hours, at which point the solvent was removed with a rotary evaporator, and the remaining product was triturated with cold pentane. This yielded a mixture of the Pt(IV) expected product, **M4**, and a Pt(II) product **M4A**. (Total mixture 19.2 mg, 79%). 1H-NMR (400 MHz, $CDCl_3$): 0.71 (s, 3H, ^2J(PtH) = 73, Pt(IV)-Me), 1.10 (s, 3H, ^2J(PtH) = 78, Pt(II)-Me), 1.18 (s, 3H, ^2J(PtH) = 14, Pt(IV)-Me), 2.96 (s, 3H, ^3J(PtH) = 15, Pt(IV)-N-CH_3), 3.25 (s, 6H, ^3J(PtH) = 21, Pt(II)-N-CH_3), 3.46 (s, 3H, ^3J(PtH) = 8, Pt(IV)-N-CH_3), 6.94-7.69 {aromatic}, 8.94 (s, 1H, ^3J(PtH) = 44, Pt(IV)-CHN), 9.58 (s, 1H, ^3J(PtH) = 58, Pt(II)-CHN) ppm. ^{13}C NMR (100.6 MHz, $CDCl_3$) δ = -10.1 (CH_3) ^1J(Pt(II)-C)= 808; -2.6 (CH_3) ^1J(Pt(IV)-C)= 652; -1.92 (CH_3) ^1J(Pt(IV)-C)= 688; 51.9 (N-CH_3) ^2J(Pt(IV)-C)= 16; 52.3 (N-CH_3) ^{2J}(Pt(IV)-C)= 18; 53.4. (N-CH_3) 2J(Pt(II)-C) = 32; 158.1 ^2J(Pt(IV)-C)= 92; 160.2 ^2J(Pt(II)-C)= 160.

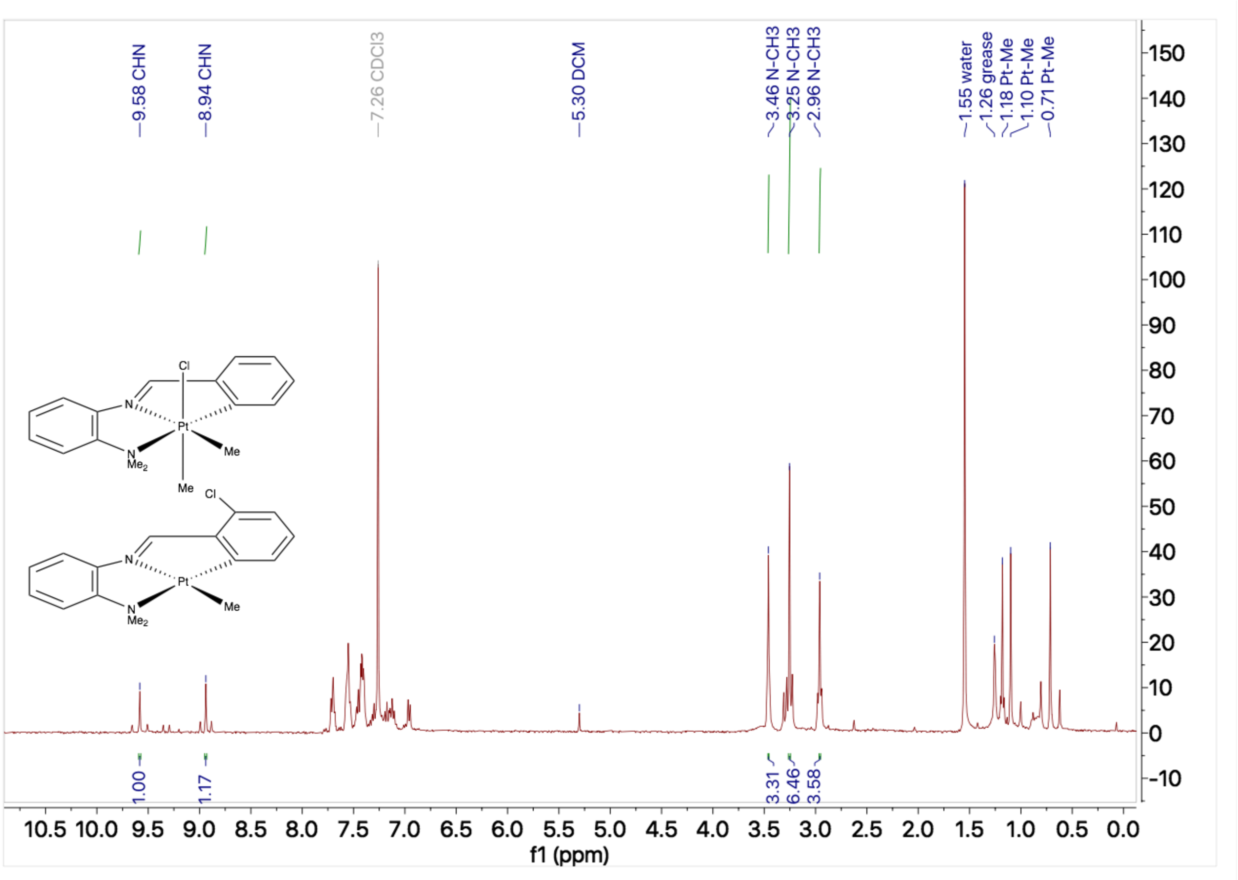

Figure S7: ^{1}H-NMR of M4 and M4A mixture.

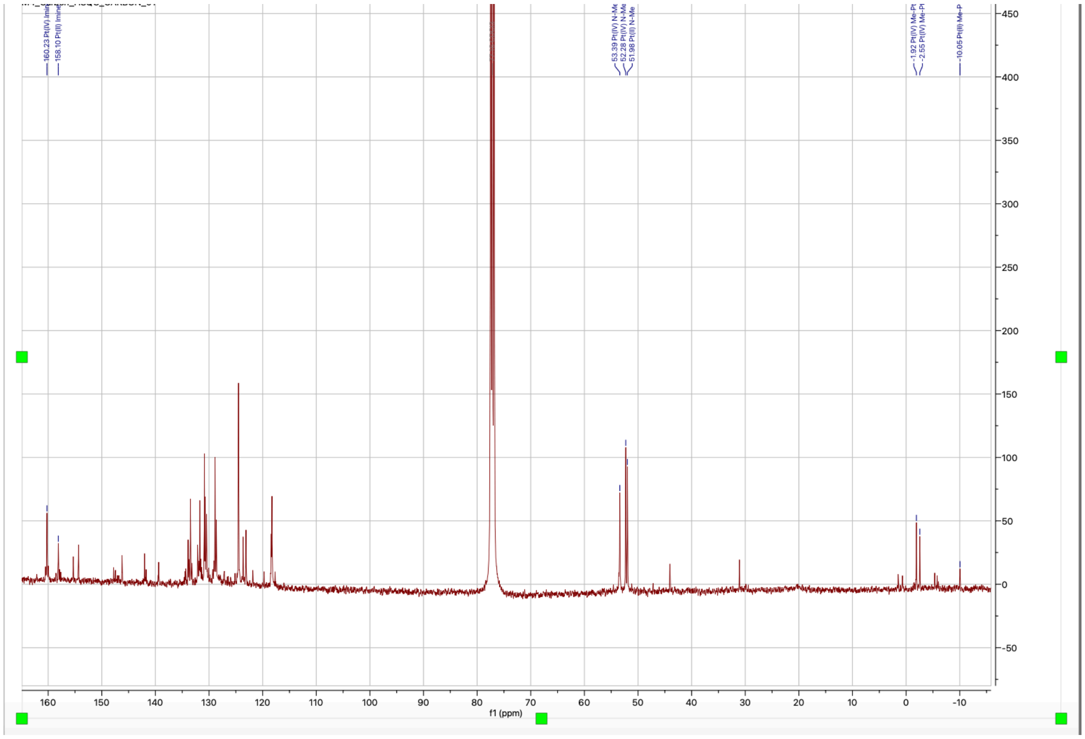

Figure S8: ^{13}C-NMR of **M4** and **M4A** mixture.

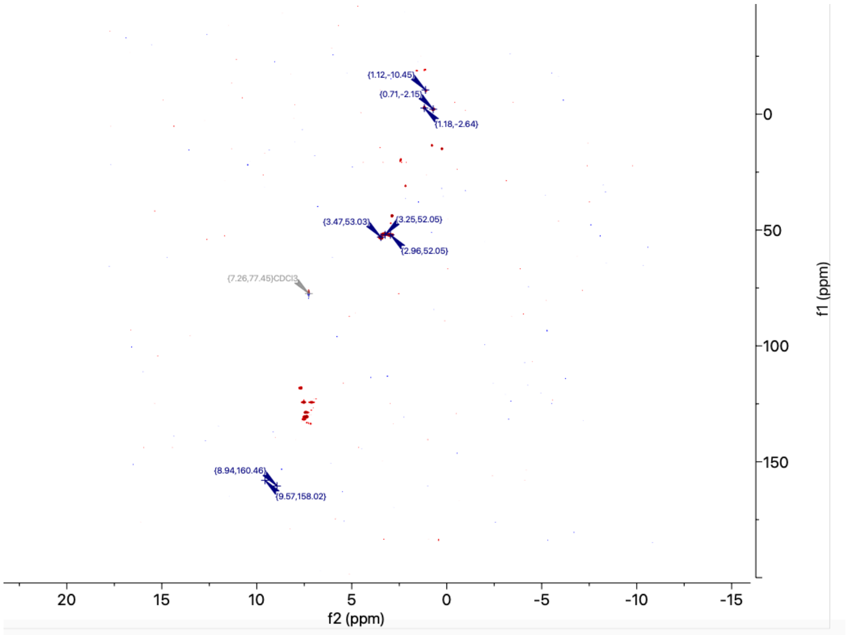

Figure S9: HSQC of **M4** and **M4A** mixture.

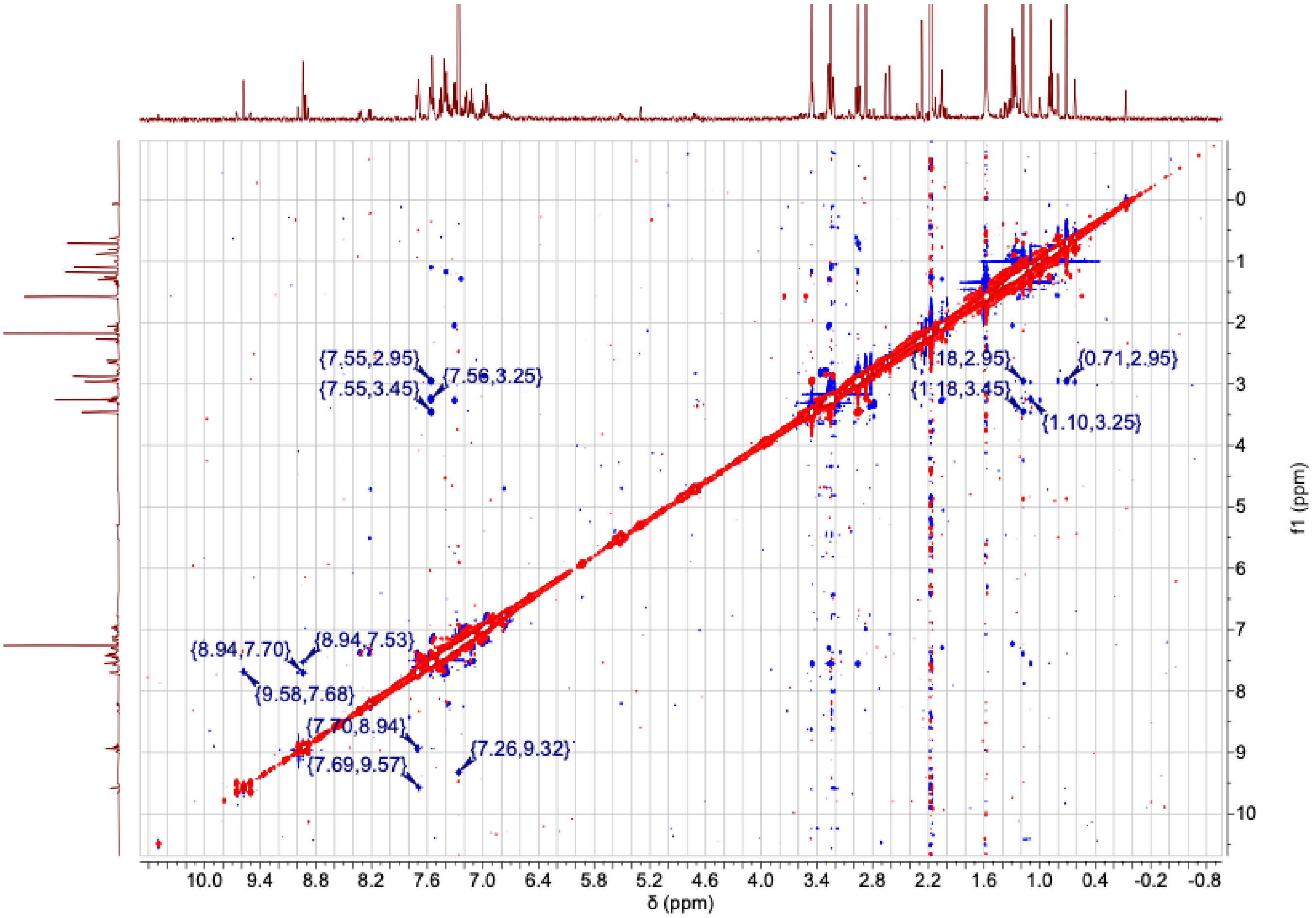

Figure S9A: NOESY of **M4** and **M4A** mixture.

M4 and M4A Kinetics Tables

Table S1: Measured rate constants as a function of temperature, where *k*=*k1*+*k2* (see text).

Temp/K	Pt(IV) NMe	Pt(IV) Imine	Pt(II) NMe	Pt(II) Imine	Pt(II) MePt	Pt(IV) MePt	STDEV
298	7.43E-05	7.10E-05	7.47E-05	7.65E-05	7.72E-05	7.54E-05	2.9%
306	0.0001918	0.0001011	0.0002017	0.0001992	0.0002026	0.0002021	22.1%
314	0.0003084	0.0002667	0.0003057	0.0003298	0.0003295	0.0003211	7.6%
322	0.000716	0.000445	0.0007731	0.0008011	0.0007459	0.0010502	25.6%

Table S2: Temperature and Rate Constants for Pt(IV) NMe at 3.45 *k2* with Eyring Plot

Temperature/K	Rate Constant/ s-1
298	3.54E-05
306	0.00008718181818
314	0.0001340869565
322	0.0002983333333

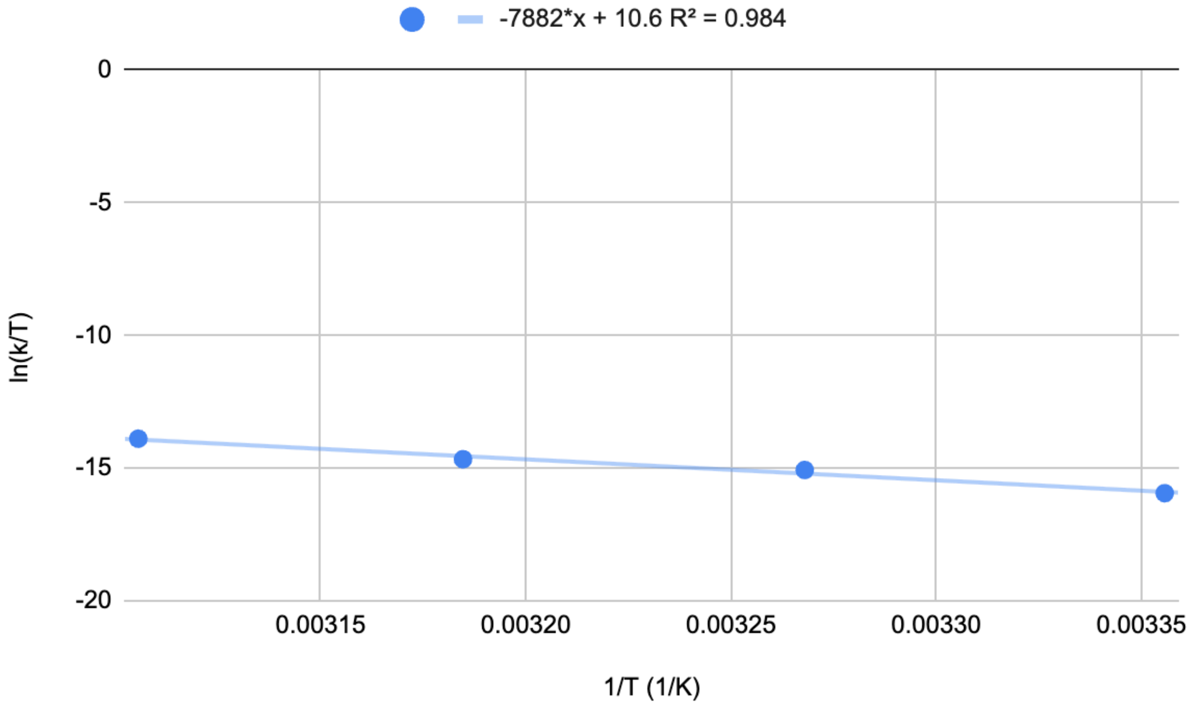

Table S3: Temperature and Rate Constant *k1* of Pt(II) N-Me at 3.24 ppm Eyring Plot.

Temperature/K	Rate Constant/s^{-1}
298	3.89E-05
306	0.0001046181818
314	0.0001743130435
322	0.0004176666667

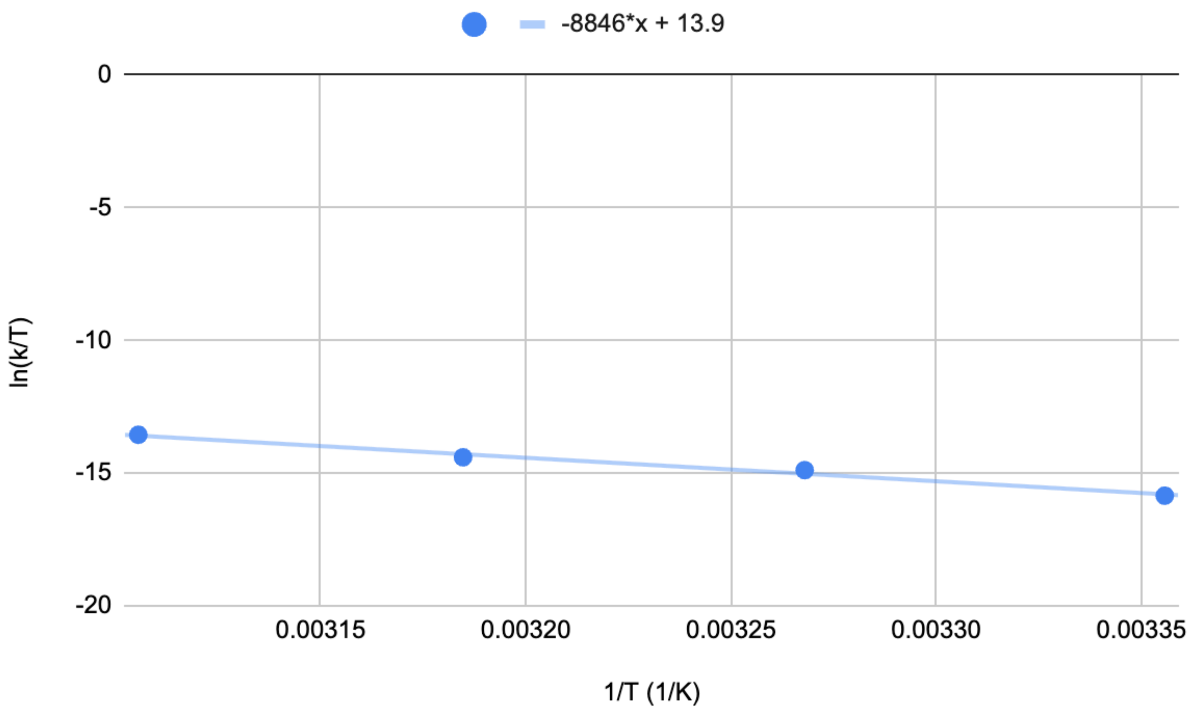
-8846*x + 13.9
0
-5
-10
-15
-20
ln(k/T)
0.00315
0.00320
0.00325
0.00330
0.00335
1/T (1/K)

Table S4: M4 (PtIV) and M4A (PtII) Activation Parameters data

		**PtIV NMe**
**ΔH‡ J/mol**		65.5E3 ± 9.0%
**ΔS‡ J/mol·K**		-109 ± 17%
**ΔG(298 K)‡ J/mol**		98.1E3 ± 8.4%
		**PtII NMe**
**ΔH‡ J/mol**		73.5E3 ± 8.1%
**ΔS‡ J/mol·K**		-81.9 ± 23%
**ΔG(298 K)‡ J/mol**		98.0E3 ± 8.4%

Figure S10: Example of Exponential Fit for **M4** and **M4A** at 298 K:

Pt(II) Imine Peak

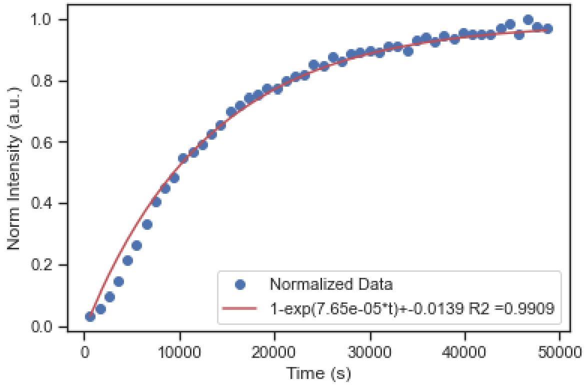

Pt(IV) Imine Peak

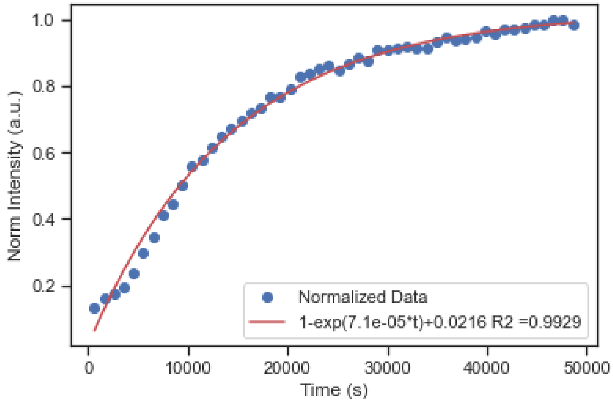

Pt(IV) N-Me Peak

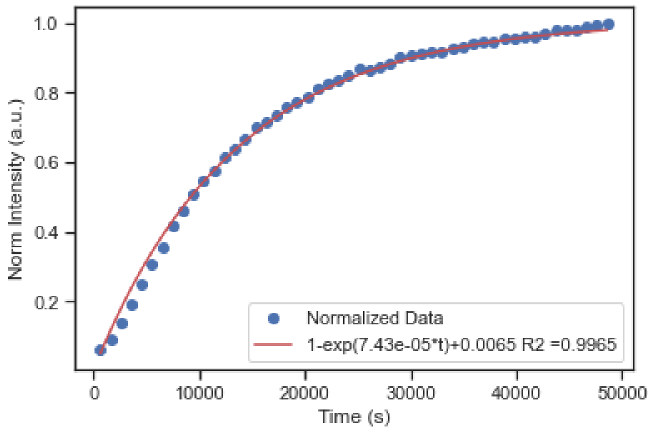

Pt(II) N-Me Peak

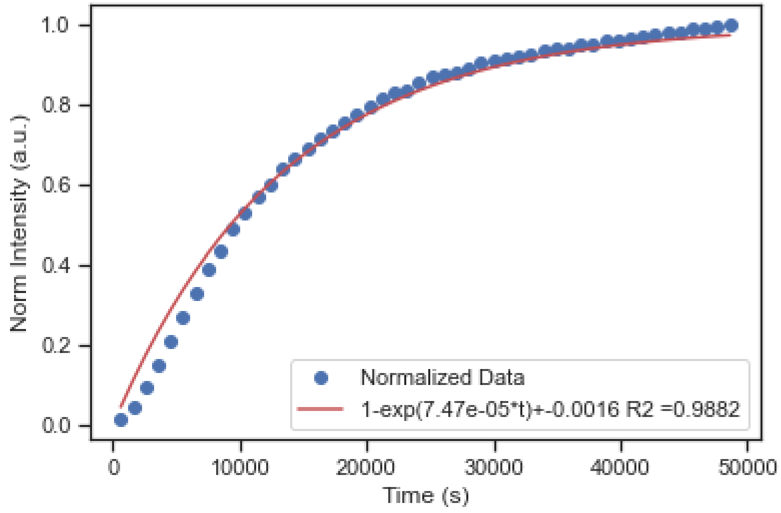

Second Pt(IV) N-Me Peak:

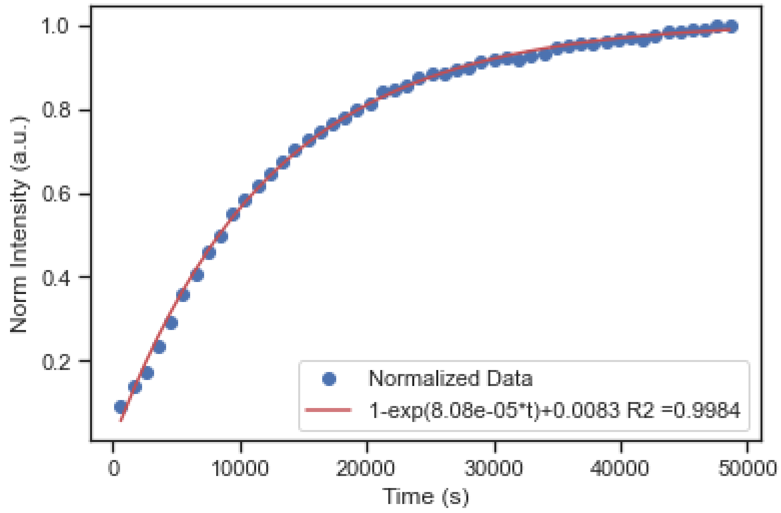

Pt(IV) Me-Pt Peak:

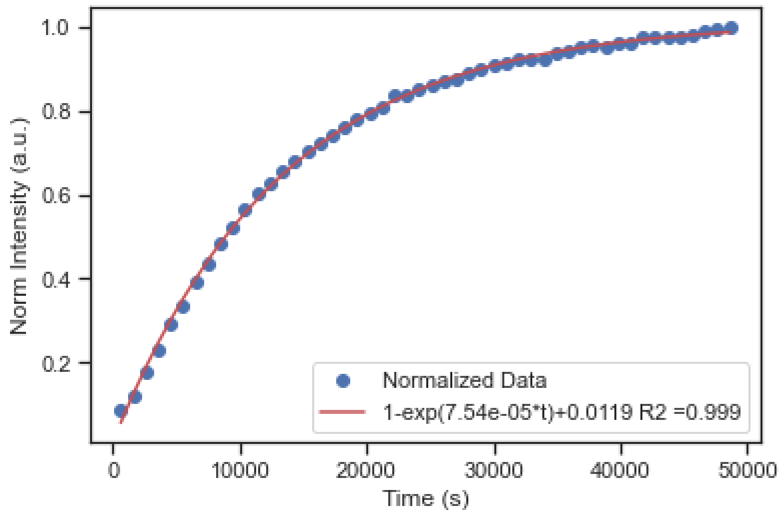

Pt(II) Me-Pt Peak

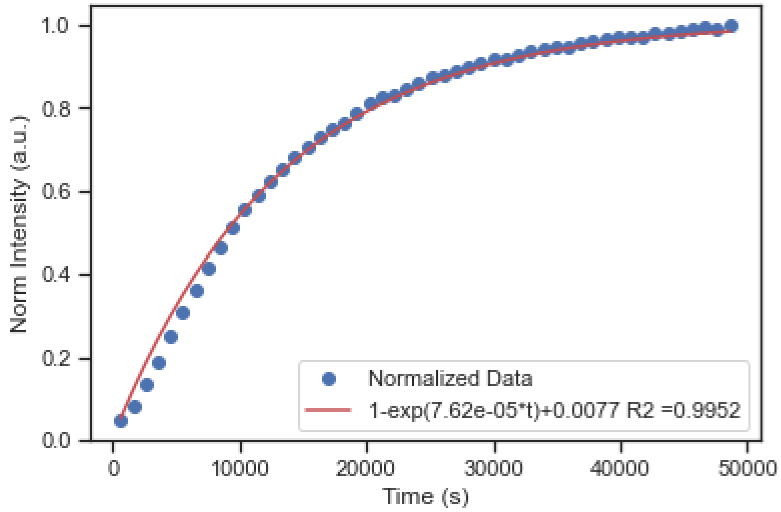

Figure S10A: Set of all four Temperatures for Pt(II) NMe and Pt(IV) NMe peaks shown in Figure 5.

M4 Pt(IV) 3.45 at 298 K

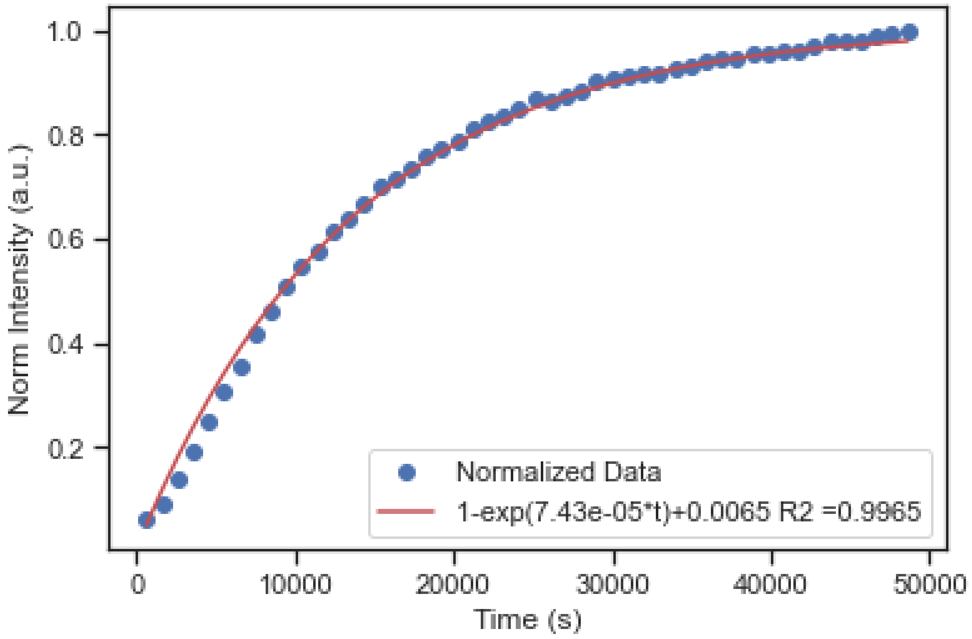

M4 Pt(IV) 3.46 at 306 K

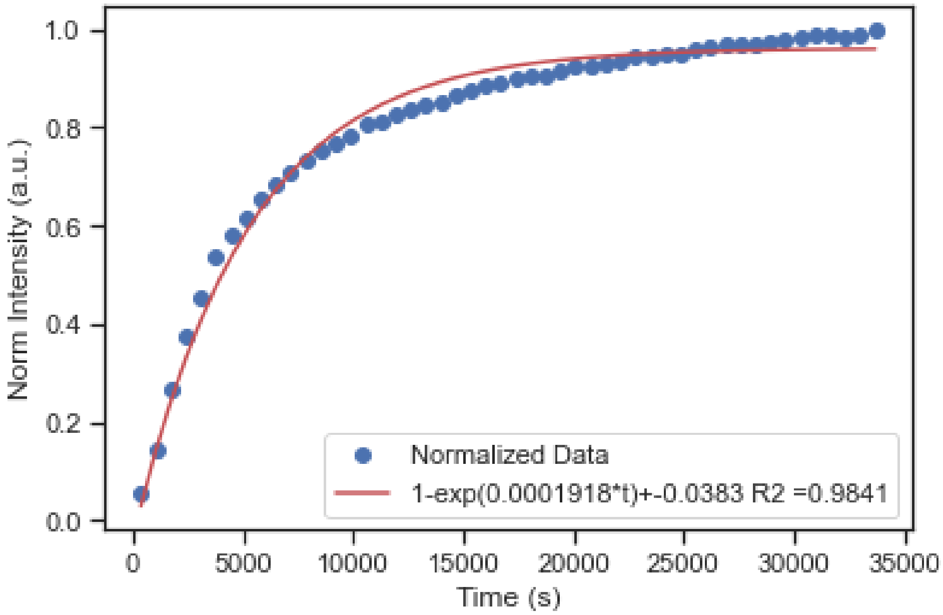

M4 Pt(IV) 3.45 at 314 K

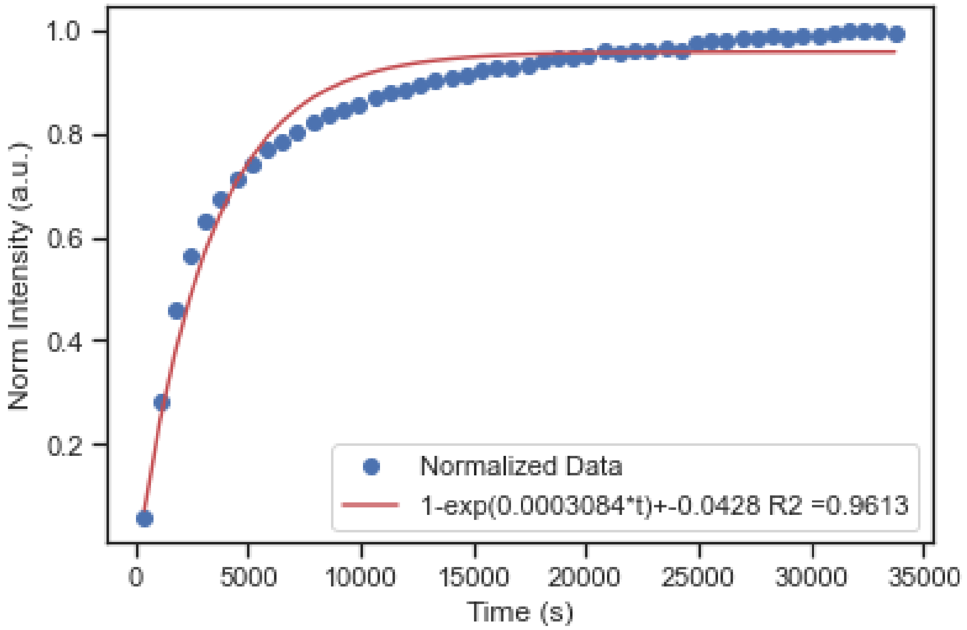

Pt (IV) NMe peak at 3.46 at 322 K

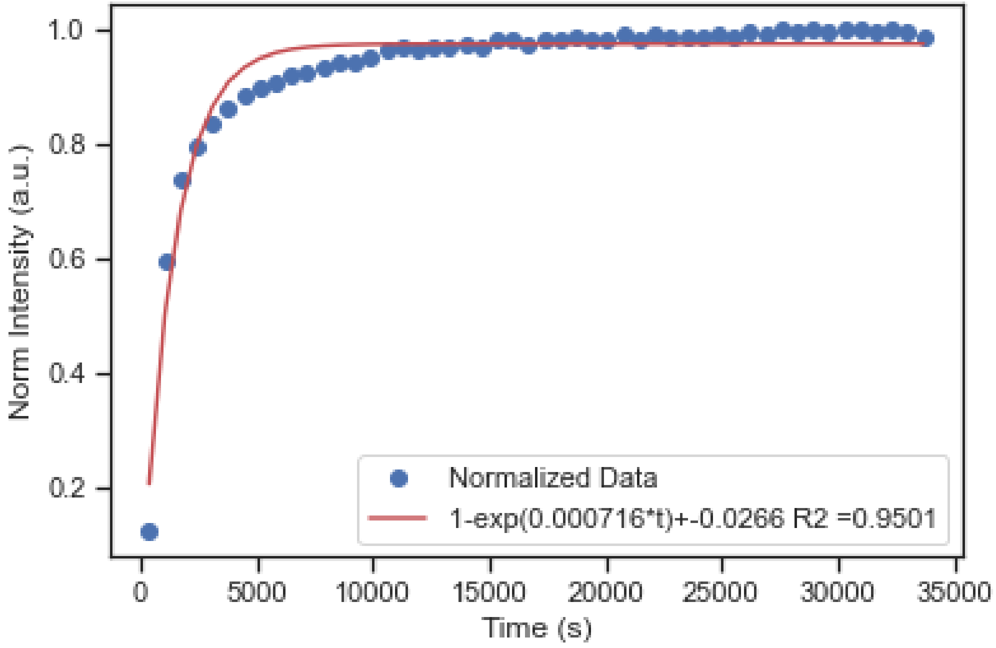

M4A Pt(II) 3.26 NMe Peak at 298 K

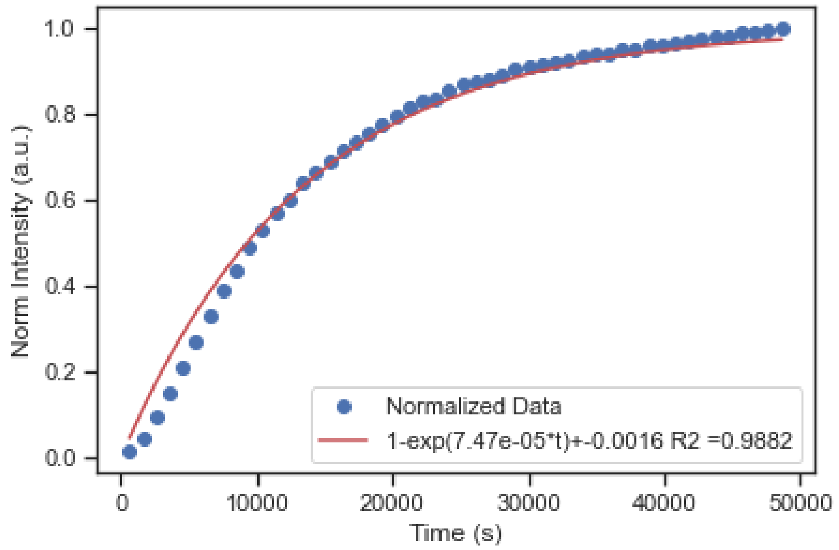

M4A Pt(II) 3.25 NMe peak at 306 K

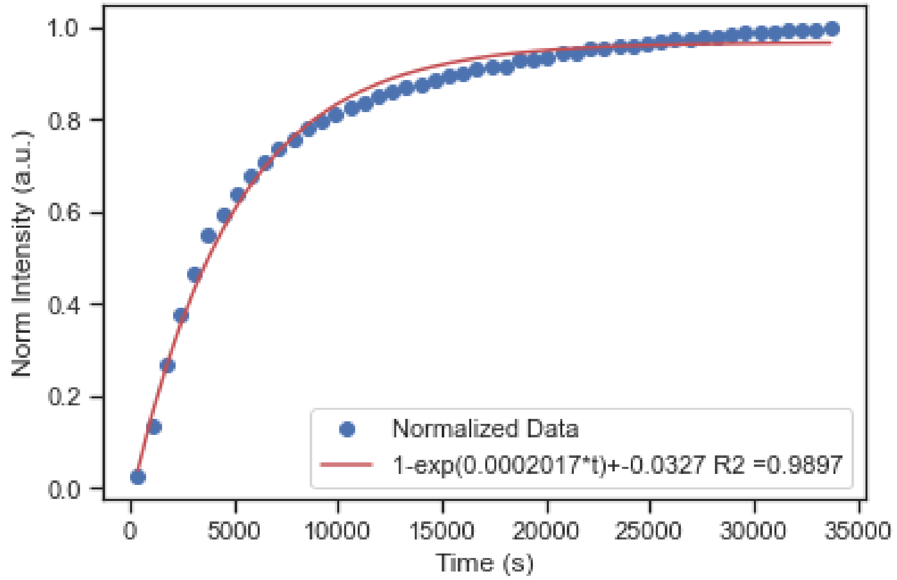

M4A Pt(II) 3.25 NMe Peak at 314 K

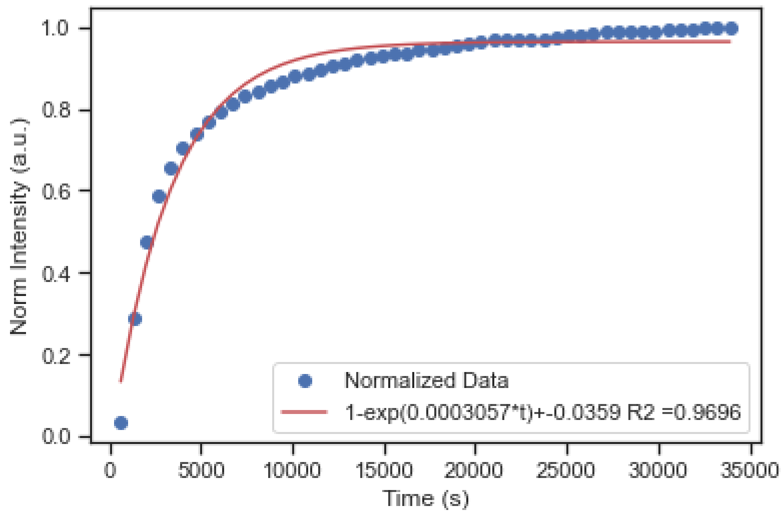

M4A Pt(II) 3.25 NMe Peak at 322 K

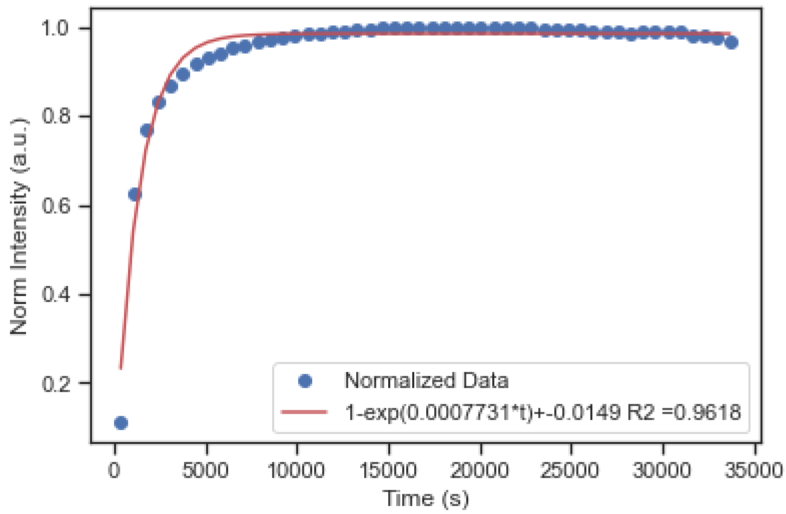

Table S5: M3 Kinetics Data: Kinetics Chart

Appearance Peaks	^{1}H Chemical Shift	Rate constant @ 298 K
Imine	8.95 ppm	0.0001954
N-CH_3	3.59 ppm	0.0001978
N-CH_3	2.95 ppm	0.0002025
Me-Pt	0.79 ppm	0.0002042
STDEV		2.0%

N.B. There is a lack of agreement with the first order model at temperatures higher than 298 K.

Figure S11: Example Plots for M3 Kinetic Runs Set of plots @ 298 K:
MePt Peak (0.822,0.745 ppm)

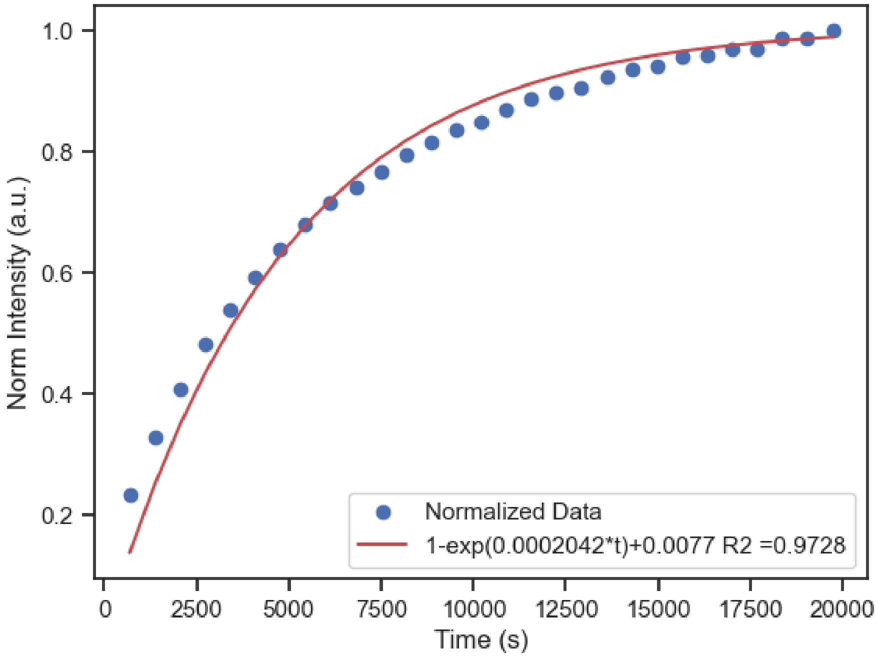

N-CH_3 Peak (2.992, 2.909 ppm)

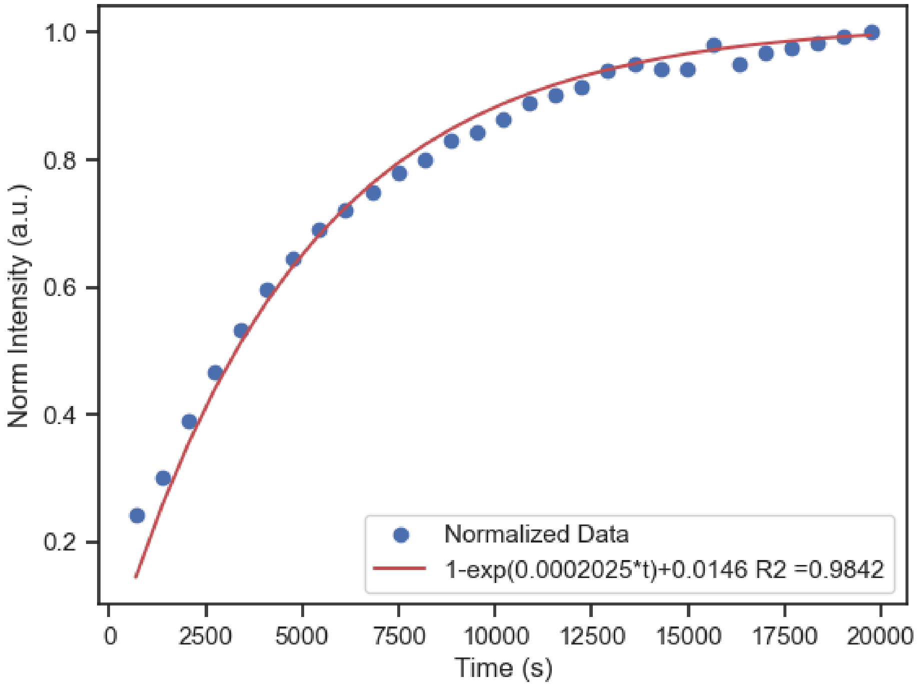

N-CH_3 Peak (3.599,3.583 ppm)

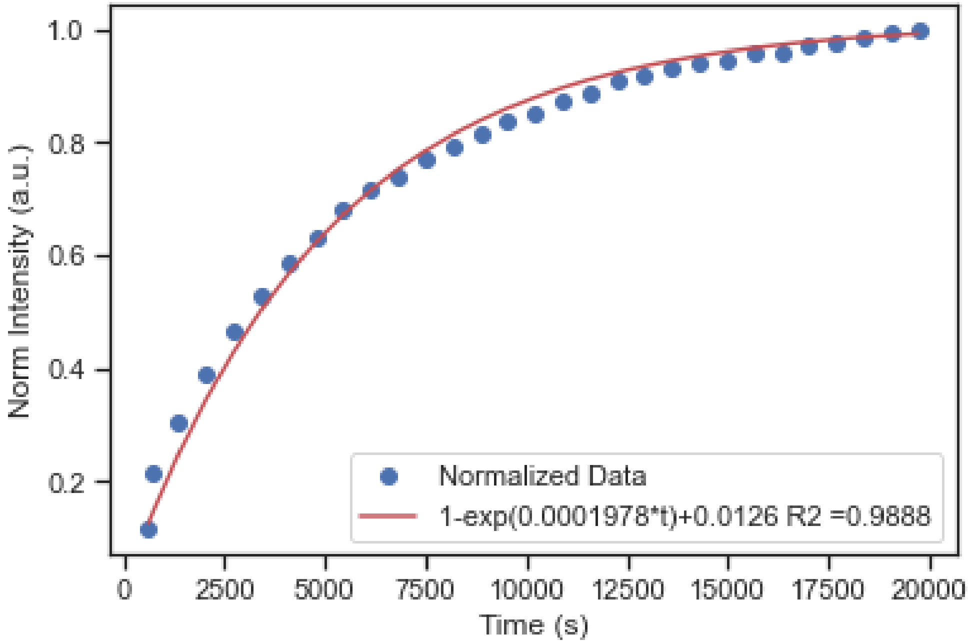

Imine Peak Appearance (8.940,8.912 ppm)

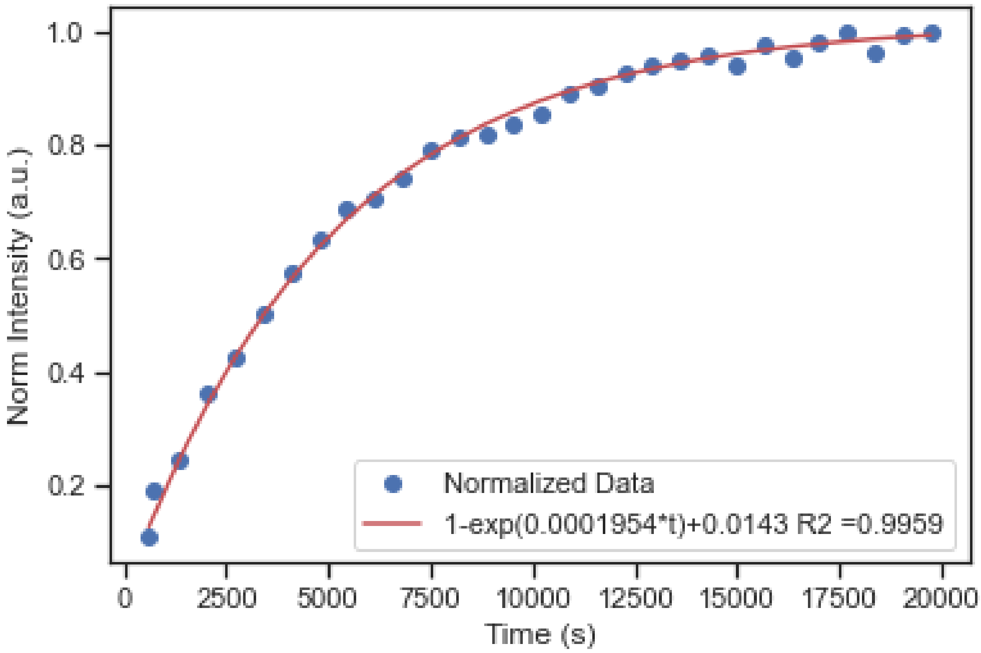

Absorption Wavelength (nm)	Extinction Coefficient: ε ($M^{-1}cm^{-1}$)	Excitation Wavelength (nm)	Emission in DCM (nm)	Stokes Shift ($\Delta\lambda_{em\ max}$-λ_{ex}) (nm)
400	5700	400	585, 635	180, 235
320	12300	320	585, 635	180, 235

Table S6. Steady State Photophysical Data for **M3**

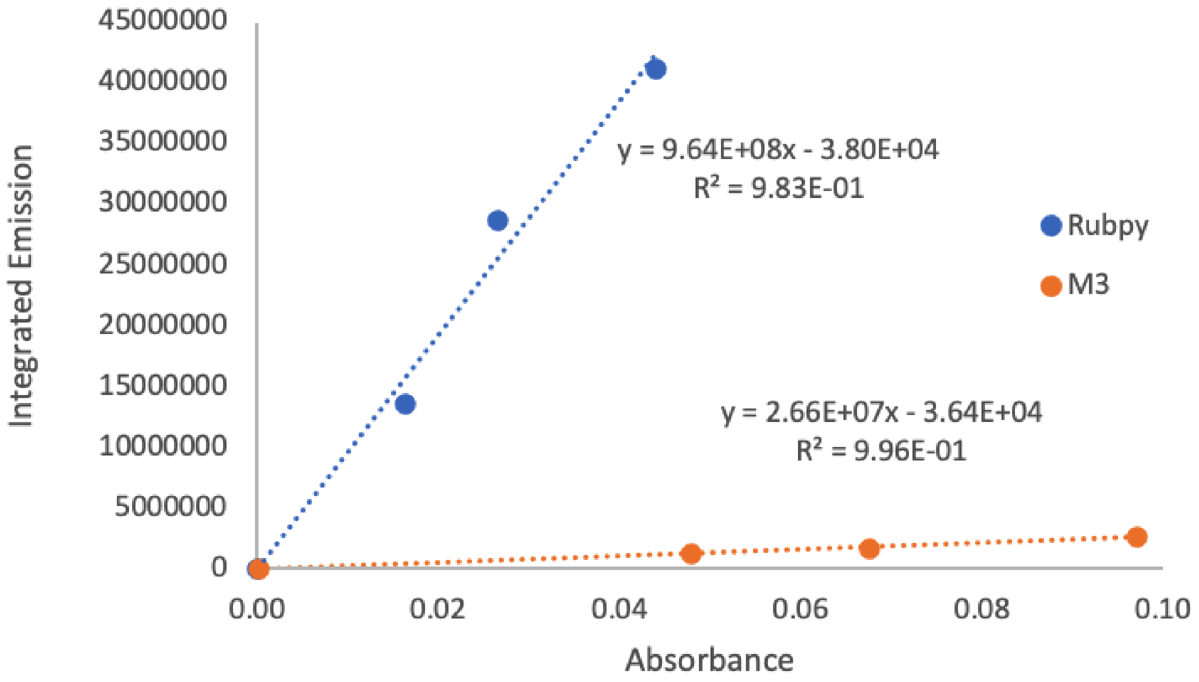

Figure S12. Relative quantum yield plot comparing the integrated emission of **M3** in DCM excited at 400 nm, at different concentrations to the integrated emission of $Ru(bpy)_3^{2+}$ in H_2O at different concentrations. The ratio of the slopes along with the ratio of the refractive indices of DCM (1.424) and H_2O (1.333) squared can be used to calculate the quantum yield of **M3**. Using the standard value of $Ru(bpy)_3^{2+}$ of 4.2%, the quantum yield of **M3** is found to be 0.13%.

Emission Wavelength (nm)	Measured Time Range (ns)	Excitation LED (nm)	Chi^2	Lifetime (ns)	Range Fitted (ns)
585	3500	405	1.112	871	378 - 3111
635	3500	405	0.997	876	372 - 3134
700	3500	405	1.021	819	370 - 3138

Table S7: Time-correlated single photon counting emission lifetimes for **M3** measured at different emission wavelengths.

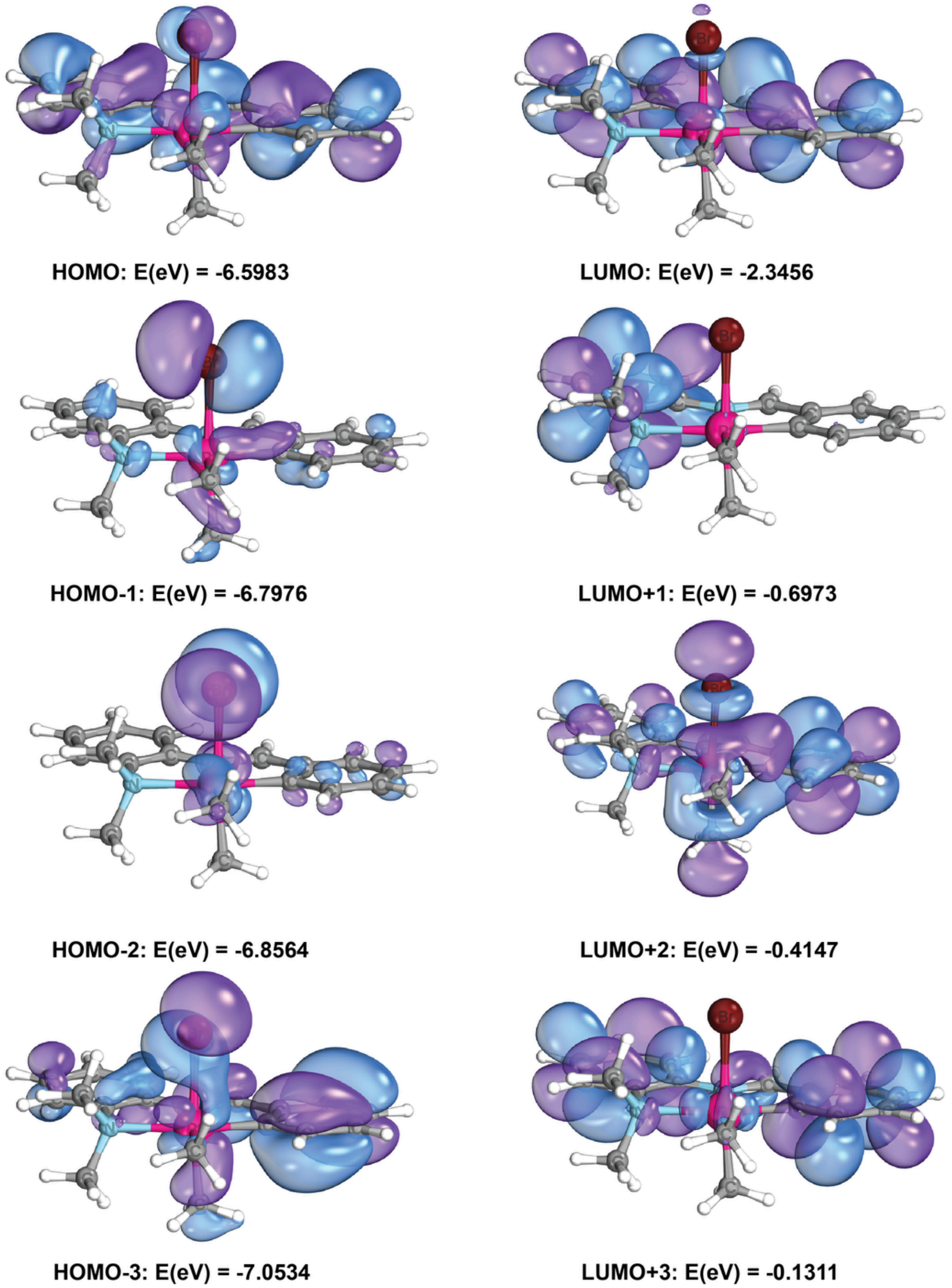

Figure S13. **M3** Frontier and near frontier molecular orbitals for **M3.**

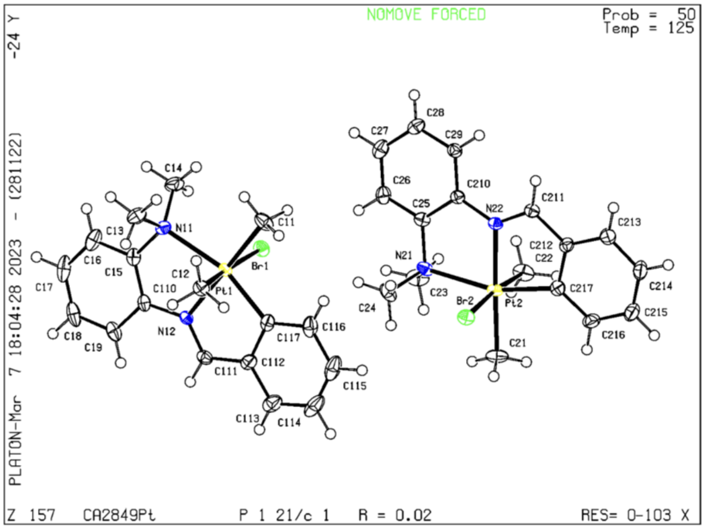

Bond Angle (º)	XRD(Pt1)	XRD(Pt2)	DFT(PBE0-D3BJ)
C17-Pt-C1	98.95(13)	97.22(13)	97.40
C17-Pt1-N2	81.18(11)	81.50(11)	81.80
C1-Pt1-N2	179.70(12)	177.75(12)	179.03
C1-Pt1-C2	86.88(12)	88.46(13)	86.56
C17-Pt1-C2	86.28(13)	87.31(13)	86.98
N2-Pt1-C2	92.86(11)	90.80(11)	92.79
C17-Pt1-N1	161.55(11)	161.67(10)	161.90
C1-Pt1-N1	99.43(12)	101.07(12)	100.65
N2-Pt1-N1	80.43(9)	80.18(9)	80.10
C2 Pt1 N1	93.13(11)	91.28(12)	92.80
C17-Pt1-Br	90.35(8)	87.48(8)	88.30
C1-Pt1-Br	91.19(10)	89.54(9)	92.56
N2-Pt1-Br	89.08(6)	92.25(6)	93.14
C2-Pt1-Br	175.81(9)	174.51(10)	176.63
N1-Pt1-Br	90.86(6)	93.73(6)	90.22

Bond Length (Å)	XRD(Pt1)	XRD(Pt2)	DFT(PBE0-D3BJ)
Pt-C17	2.002(3)	1.999(3)	1.977
Pt-C1	2.055(3)	2.050(3)	2.048
Pt-N2	2.055(2)	2.052(2)	2.058
Pt-C2	2.063(3)	2.068(3)	2.053
Pt-N1	2.253(2)	2.262(2)	2.271
Pt-Br	2.5927(4)	2.6037(4)	2.609

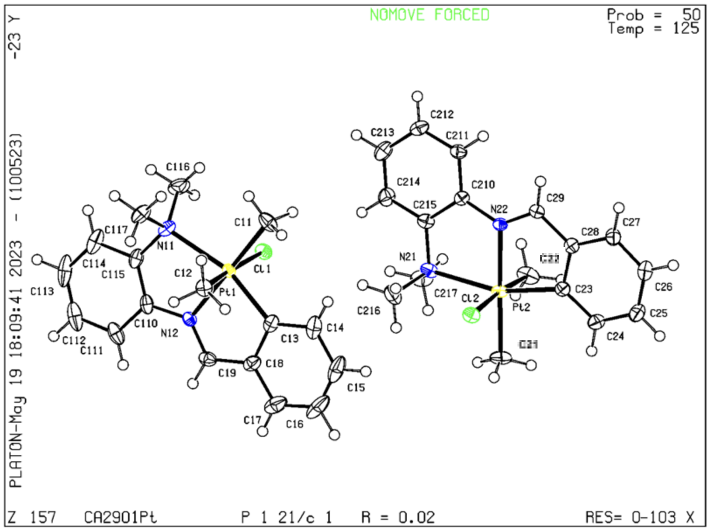

Bond Angle (º)	XRD(Pt1)	XRD(Pt2)	DFT(PBE0-D3BJ)
C3-Pt-N2	81.15(10)	81.28(9)	81.88
C3-Pt-C1	98.96(12)	97.98(11)	97.35
N2-Pt-C1	179.62(10)	178.43(10)	179.20
C3-Pt-C2	86.72(10)	88.55(11)	86.56
N2-Pt-C2	92.18(9)	91.50(10)	87.11
C1-Pt-C2	87.47(11)	87.10(11)	86.84
C3-Pt-N1	161.67(10)	161.57(9)	161.99
N2-Pt-N1	80.58(8)	80.29(8)	80.14
C1-Pt-N1	99.30(11)	100.43(10)	100.65
C2-Pt-N1	92.42(9)	91.49(11)	92.95
C3-Pt-Cl	90.33(7)	87.88(7)	89.36
N2-Pt-Cl	89.39(6)	91.39(6)	87.91
C1-Pt-Cl	90.97(8)	89.96(8)	92.26
C2-Pt-Cl	176.41(8)	175.03(9)	176.21
N1-Pt-Cl	91.02(6)	92.99(6)	90.84

Bond Length (Å)	XRD(Pt1)	XRD(Pt2)	DFT(PBE0-D3BJ)
Pt-C1	2.056(3)	2.050(3)	2.046
Pt-C2	2.061(2)	2.062(3)	2.049
Pt-N2	2.0515(19)	2.0563(19)	2.059
Pt-C3	1.998(3)	2.000(3)	1.976
Pt-N1	2.249(2)	2.265(2)	2.269
Pt-Cl	2.4751(6)	2.4754(6)	2.465

*For the purpose of simplifying the presentation of the columns for the two molecules, the labels for atoms C21 and C22 bonded to Pt2 have been switched as compared to the manuscript’s Figure 3 and its caption.

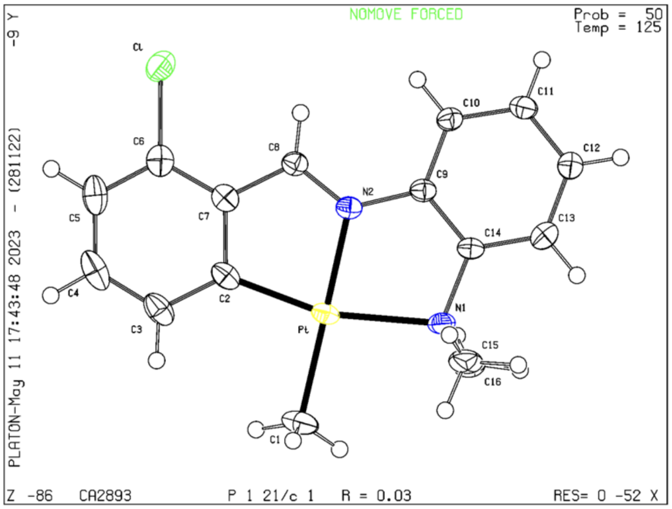

Bond Angle (º)	XRD(Pt1)	DFT(PBE0-D3BJ)
C2-Pt-N2	81.92(14)	81.76
C2-Pt-C1	97.61(17)	96.75
N2-Pt-C1	179.12(16)	178.50
C2-Pt-N1	164.00(13)	163.66
N2-Pt-N1	82.09(12)	81.90
C1-Pt-N1	98.38(16)	99.59

Bond Length (Å)	XRD(Pt1)	DFT(PBE0-D3BJ)
Pt-C2	1.983(4)	1.969
Pt-N2	2.014(3)	2.017
Pt-C1	2.075(4)	2.045
Pt-N1	2.169(3)	2.181

Table S8: Bond Lengths and Angles Comparison of Experimental Values with calculated values for **M3, M4, and M4A.**

Loewdin Atom Pop.	HOMO-3	HOMO-2	HOMO-1	HOMO	LUMO	LUMO+1	LUMO+2	LUMO+3
Pt	9	15.8	8.5	12.5	2.5	0.8	20.7	2.5
Br	23.3	69.4	73.6	4.9	1.4	0.3	10	0.2

Table S9

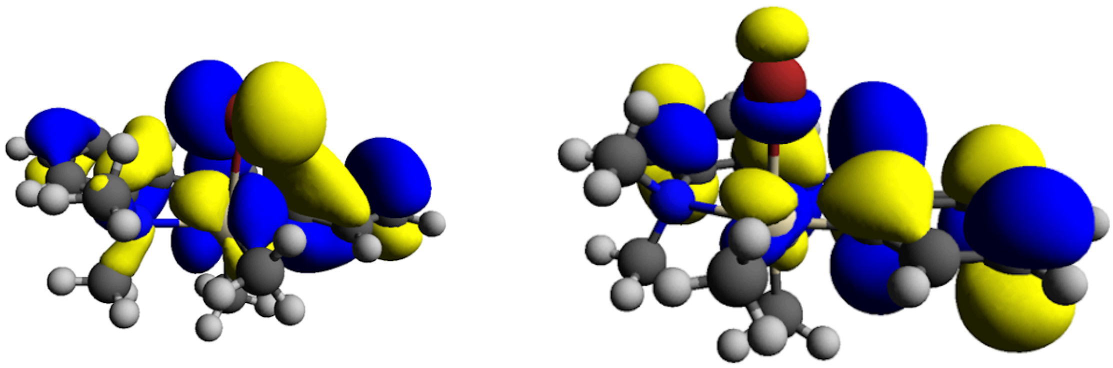

Figure S14. **M3** State 1 (3.359 eV) Major Contributing Hole (left) and Electron (right) Natural Transition Orbitals (n=0.99). Largest MO excitation weight: HOMO→ LUMO (n=0.89).

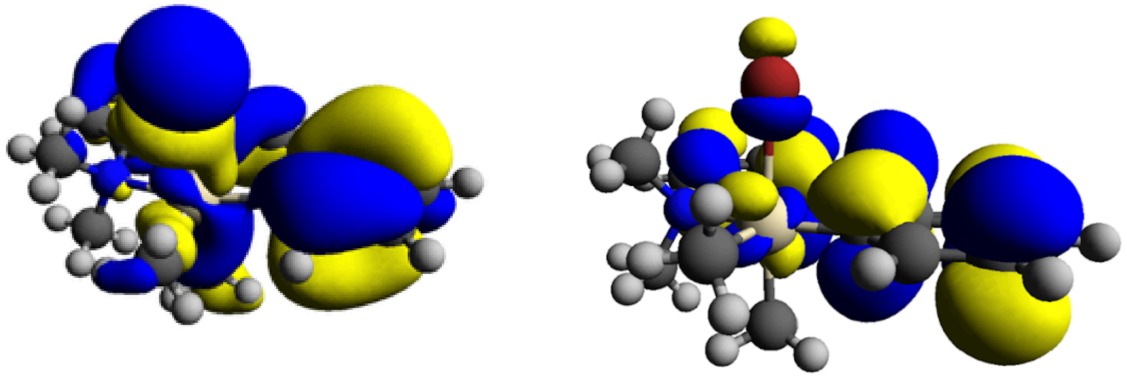

Figure S15. **M3** State 4 (3.883 eV) Major Contributing Hole (left) and Electron (right) Natural Transition Orbitals (n=0.98). Largest MO excitation weight: HOMO-3 → LUMO (n=0.88)

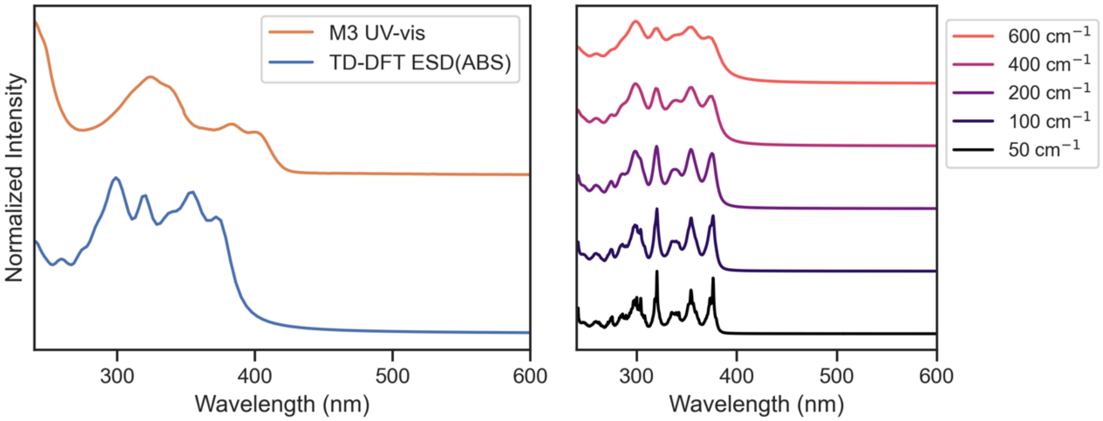

Figure S16. **M3** TD-DFT ORCA_ESD absorbance calculation. Spectra are shown with varied LINEW input flag for broadening as indicated in the legend.

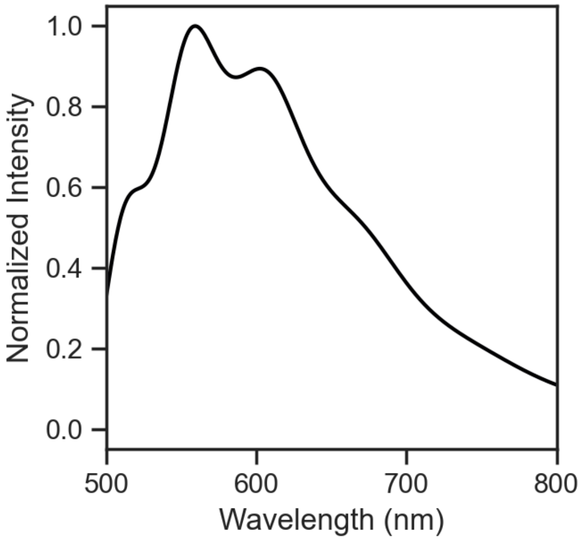

Figure S17. **M3** TD-DFT ORCA_ESD phosphorescence spectrum calculation. INLINEW and LINEW broadening parameters of 200 cm-1 and 75 cm-1 are used as input flags.

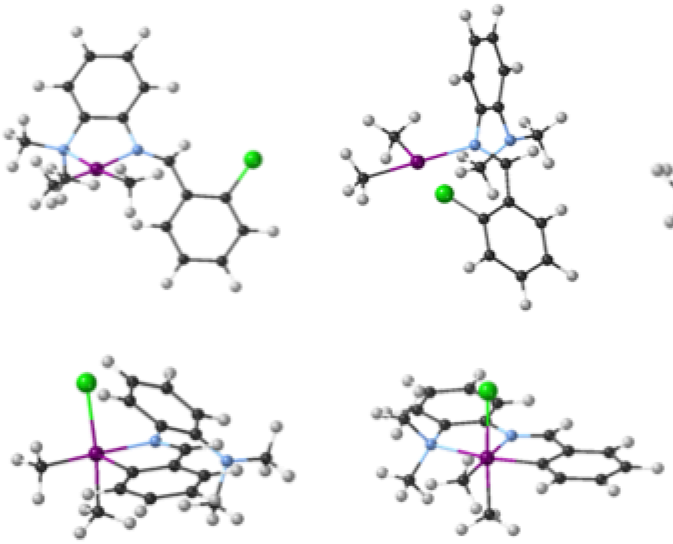

Figure S18: 3D structures for reaction pathway for C-Cl oxidative addition. Reaction pathway for C-Cl oxidative addition Species from left to right starting at top correspond to “ICl, 2Cl, 3Cl, 4Cl, and M4,” labeled in reaction energy diagram Figure 6.

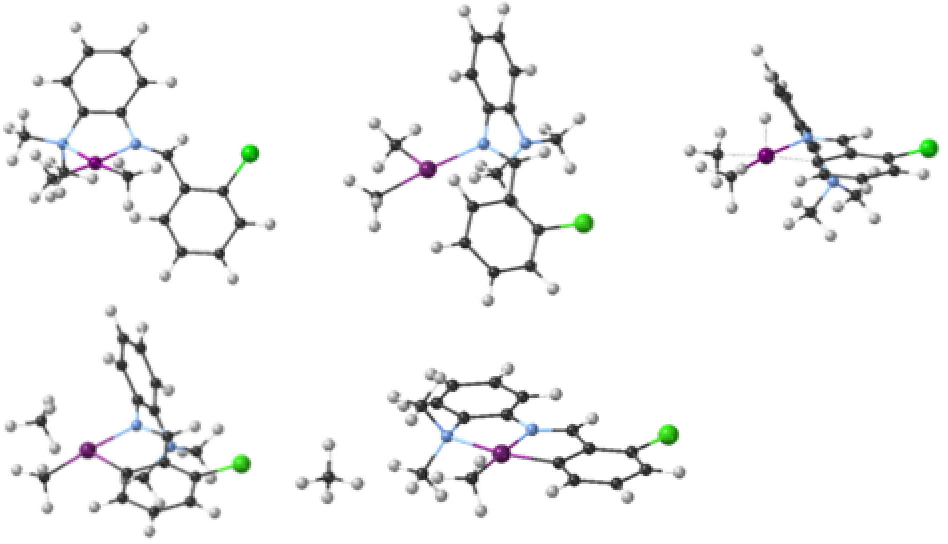

Figure S19: 3D structures for reaction pathway for C-H oxidative addition. Reaction pathway for C-Cl oxidative addition Species from left to right starting at top correspond to “IH, 2Cl, 3Cl, 4Cl, and M4A + CH4,” labeled in reaction energy diagram Figure 6.

Table S10: Energies and Coordinations for Computational Results:

	Enthalpy (hartrees)	298.15K Entropy Correction (hartrees)	298.15 K Gibbs (hartrees)
1Cl	-20270.51406	-0.07114517	-20270.58521
2Cl	-20270.49479	-0.07286712	-20270.56766
3Cl (TS)	-20270.48121	-0.07159718	-20270.5528
4Cl	-20270.53643	-0.07291038	-20270.60934
M4	-20270.56336	-0.06886965	-20270.63223

	Enthalpy (hartrees)	298.15K Entropy Correction (hartrees)	298.15 K Gibbs (hartrees)
1Br	-22432.28225	-0.0722152	-22432.35447
2Br	-22432.26448	-0.07369767	-22432.33818
3Br (TS)	-22432.25368	-0.07274092	-22432.32642
4Br	-22432.31163	-0.07331161	-22432.38495
M3	-22432.33866	-0.06981723	-22432.40848

	Enthalpy (hartrees)	298.15K Entropy Correction (hartrees)	298.15 K Gibbs (hartrees)
1H	-20270.51406	-0.07114517	-20270.58521
2H	-20270.49592	-0.07337674	-20270.56929
3H	-20270.48024	-0.07206964	-20270.55231
4H	-20270.51376	-0.0734085	-20270.58716
M4A+CH4	-20270.53628	-0.07670068	-20270.61298

1Cl/1H xyz coordinates (identical starting geometry for both pathways)

Pt	1.23612765	0.07399409	-0.8745549
C	3.13349942	0.33628531	-1.5394578
H	3.30344277	1.37984678	-1.8382715
H	3.85251839	0.11331386	-0.737595
H	3.39868557	-0.2928569	-2.3955996
N	-0.6949422	-0.0332229	-0.0538014
C	-0.9984613	1.26039254	0.44030634
C	0.0767308	2.00437373	0.94101667
N	1.37663451	1.37223202	0.9357039
C	1.53711988	0.51976619	2.12960933
H	2.48518519	-0.0112142	2.05143949
H	1.53292997	1.13243318	3.0388196
H	0.72784615	-0.2060117	2.17829734
C	2.4821621	2.33066793	0.88086014
H	3.40626788	1.77355276	0.7424377
H	2.34325532	3.00057383	0.0342064
H	2.55931439	2.91132171	1.80590341
C	-0.145834	3.28594332	1.41485126
C	-1.4214411	3.8361917	1.37444665
C	-2.4798315	3.10487981	0.86212253
C	-2.2689831	1.81587753	0.39562205
H	-3.0844979	1.25654486	-0.0473947
H	-3.4701182	3.54066226	0.80540643
H	-1.5786985	4.84599014	1.73437325
H	0.6742312	3.87141957	1.80852376
C	-1.5615776	-0.9635929	0.11249536
C	-1.3652251	-2.3605084	-0.2376826
C	-2.4573106	-3.171486	-0.5640976
C	-2.3032792	-4.5192696	-0.8359672
C	-1.040145	-5.0858718	-0.765619
C	0.0580132	-4.3069428	-0.4254881
C	-0.1057637	-2.9605468	-0.1652532
H	0.7423372	-2.3350125	0.09173457
H	1.04374958	-4.7516566	-0.3621099
H	-0.9190227	-6.1421831	-0.975304
H	-3.1670044	-5.1143527	-1.1038044
H	-2.5085401	-0.718839	0.59119975
C	0.91893466	-1.0342872	-2.5371724
H	1.21851358	-0.4890139	-3.4396518
H	1.49291267	-1.9685869	-2.5200053
H	-0.1406235	-1.2977471	-2.6369483
Cl	-4.0452235	-2.4885742	-0.6786751

2Cl xyz coordinates

Pt	-0.1231672	0.02504992	-2.318798
C	0.9624806	1.72252291	-2.2431398
H	0.78094209	2.28065678	-1.3207216
H	2.02941263	1.47850756	-2.2973524
H	0.72688316	2.37197308	-3.0913911
N	-0.7069115	0.05515644	-0.2790697
C	-0.8991343	1.32770821	0.33812286
C	-0.109595	1.73587858	1.43061751
N	0.89738296	0.90090935	1.93700368
C	1.3797452	1.20285052	3.26383437
H	1.9918516	0.36621295	3.60591343
H	2.00096667	2.1111554	3.30511335
H	0.54274972	1.32359238	3.95299066
C	1.98604075	0.59421932	1.02524392
H	1.60936654	0.29796221	0.04754337
H	2.6522589	1.45847188	0.88626491
H	2.57150584	-0.2311785	1.43520158
C	-0.3996807	2.97941639	1.99735942
C	-1.4142878	3.78504634	1.5031792
C	-2.1590495	3.37941111	0.40780674
C	-1.8866481	2.15180827	-0.1742198
H	-2.4478333	1.8034694	-1.0334418
H	-2.9454209	4.00766288	0.00746233
H	-1.608105	4.74303442	1.97215834
H	0.1888496	3.32908163	2.83536835
C	-0.9005531	-0.9483691	0.49134128
C	-0.713043	-2.3578298	0.17137236
C	-0.8617723	-2.9643793	-1.0766355
C	-0.6508759	-4.316832	-1.2622163
C	-0.2717234	-5.1024691	-0.1835523
C	-0.1306449	-4.5354502	1.07382129
C	-0.3653363	-3.1834859	1.24483206
H	-0.2686851	-2.7333084	2.22684767
H	0.15386407	-5.1463989	1.92154003
H	-0.0970542	-6.1613095	-0.3321508
H	-0.7829759	-4.7492453	-2.2459356
H	-1.1823094	-0.7570087	1.52591417
C	0.38748819	-0.0420199	-4.2813864
H	-0.1383279	0.73413706	-4.8512173
H	1.46031051	0.12400212	-4.4311541
H	0.1378149	-1.0106034	-4.731623
Cl	-1.3967802	-2.0500103	-2.4528453

3Cl .xyz coordinates (TS)

Pt	-0.446910233	-0.321552113	-2.100333814
C	1.230197675	0.796264177	-2.216858869
H	1.148219865	1.646692039	-1.5328209
H	2.098284926	0.196492079	-1.926383853
H	1.397383455	1.168442227	-3.230084357
N	-0.789094991	0.131023579	-0.025969564
C	-0.916162433	1.444558417	0.483858209
C	-0.196742321	1.90729	1.607020239
N	0.724094279	1.079277674	2.265602741
C	1.124009428	1.479041321	3.593893739
H	1.667529938	0.651063163	4.052931441
H	1.785233927	2.359615306	3.603275505
H	0.246882732	1.696971965	4.204604042
C	1.860722543	0.641167355	1.473533184
H	1.546704758	0.297005932	0.491331281
H	2.590978933	1.45217712	1.334594882
H	2.357125688	-0.185942983	1.98531459
C	-0.459909675	3.207904077	2.042896507
C	-1.371420263	4.02639191	1.39308348
C	-2.030751917	3.575968865	0.261302092
C	-1.785135495	2.290163565	-0.190649398
H	-2.285508111	1.903415923	-1.071570863
H	-2.730065708	4.213610868	-0.265657933
H	-1.545017736	5.029420331	1.765978923
H	0.072563524	3.59454708	2.901758528
C	-0.857451391	-0.881878356	0.759494571
C	-0.669431746	-2.1944081	0.194456446
C	-0.955423725	-2.406007181	-1.173909639
C	-0.53195256	-3.576246962	-1.802013506
C	0.184075583	-4.512792217	-1.078160363
C	0.435740349	-4.339470691	0.28054615
C	0.004862297	-3.183050617	0.906217651
H	0.230526259	-3.002504237	1.952100857
H	0.971561587	-5.096334741	0.83916386
H	0.523290901	-5.411813828	-1.580294846
H	-0.780514897	-3.747470316	-2.841142444
H	-0.973255191	-0.757062254	1.833276678
C	-0.139070136	-0.806105355	-4.047516388
H	-0.25971746	0.069901972	-4.694644528
H	0.870792522	-1.195938007	-4.213075335
H	-0.855468481	-1.56860262	-4.372970746
Cl	-2.4917767	-1.671226368	-1.862178249

4Cl .xyz coordinates

Pt	-0.6940484	-0.3844826	-2.0807874
C	1.295909	0.00410967	-2.2571923
H	1.36017745	1.07547595	-2.4802876
H	1.83135097	-0.1994393	-1.3289401
H	1.75757827	-0.5627351	-3.0651951
N	-0.6028052	0.17457769	0.01596284
C	-0.8031224	1.47456655	0.52144516
C	-0.2021151	1.99013605	1.69761601
N	0.72824424	1.24332888	2.43275884
C	0.97536277	1.65271631	3.79513811
H	1.54371186	0.86367236	4.29146331
H	1.55879604	2.58321085	3.87062318
H	0.03314816	1.78664975	4.32754596
C	1.96439125	0.92015743	1.74139835
H	1.77453547	0.65384758	0.70492953
H	2.65870625	1.77281256	1.75013182
H	2.44994162	0.07406727	2.23294385
C	-0.5856291	3.26697782	2.10961021
C	-1.4890368	4.03346654	1.38962976
C	-2.0244918	3.54694889	0.20980865
C	-1.670528	2.27809875	-0.2131739
H	-2.1219676	1.85764697	-1.1042424
H	-2.7216729	4.1373252	-0.3718742
H	-1.7552291	5.02084745	1.74929898
H	-0.1486509	3.68061994	3.00866552
C	-0.3541921	-0.8512964	0.75054537
C	-0.1456333	-2.1067725	0.08543856
C	-0.2091192	-2.1089435	-1.3187125
C	0.02035403	-3.2757237	-2.0226604
C	0.2950658	-4.4456902	-1.3198263
C	0.35120767	-4.458899	0.06875721
C	0.13302007	-3.2889019	0.77226716
H	0.17648619	-3.2735976	1.85612675
H	0.56740165	-5.3802944	0.5954885
H	0.46843672	-5.361112	-1.8747001
H	-0.0175508	-3.2931723	-3.1029276
H	-0.2792697	-0.7719195	1.83102246
C	-0.7357856	-0.9139619	-4.0358528
H	-0.9327765	0.00977304	-4.5949445
H	0.20568269	-1.3402276	-4.3841332
H	-1.5532167	-1.610969	-4.224045
Cl	-3.1026679	-0.472897	-2.0291216

M4 .xyz coordinates

Pt	1.81913496	0.04241013	-0.6404705
C	3.86223137	-0.0697284	-0.6531786
H	4.27399275	0.52365978	0.17119793
H	4.21716274	-1.0962858	-0.5359618
H	4.24788154	0.3329244	-1.5924275
N	-0.2377473	0.13065264	-0.6422853
C	-0.817282	1.10617133	0.16254894
C	0.05223967	1.88967159	0.93665992
N	1.4975452	1.64310637	0.93497579
C	1.91598994	1.19764767	2.27695957
H	2.97206481	0.93163071	2.24143202
H	1.77537234	1.99860557	3.00811324
H	1.33377778	0.3307347	2.57773527
C	2.24339421	2.87400696	0.60811421
H	3.30137873	2.62173902	0.54508146
H	1.9125287	3.25085654	-0.3550224
H	2.10661996	3.63098886	1.38475761
C	-0.4770569	2.89420264	1.73492569
C	-1.8410672	3.12630369	1.77489049
C	-2.6999656	2.34685998	1.0105692
C	-2.1906697	1.34518269	0.20952392
H	-2.8599347	0.74427465	-0.393518
H	-3.7684206	2.52441631	1.0361892
H	-2.2328533	3.91702372	2.4032625
H	0.18224991	3.50655481	2.33710906
C	-0.7976532	-0.693828	-1.4563773
C	0.08986606	-1.5379706	-2.2133173
C	1.48089747	-1.3526945	-1.9988392
C	2.36418209	-2.1308396	-2.7263583
C	1.88798995	-3.0645932	-3.6426944
C	0.52416202	-3.2427705	-3.8495723
C	-0.3778952	-2.4765928	-3.1345895
H	-1.4461111	-2.5951926	-3.2843672
H	0.17265531	-3.9746185	-4.5668412
H	2.59627194	-3.6636957	-4.2052411
H	3.43250782	-2.0154236	-2.5892847
H	-1.8762862	-0.7484627	-1.5895621
C	1.85836202	-1.4650549	0.74744085
H	2.08873217	-2.415008	0.264861
H	2.62510092	-1.2615154	1.49489165
H	0.88334436	-1.5444282	1.23468903
Cl	1.80408887	1.74401916	-2.4243016

1Br .xyz coordinates

Pt	1.23040165	0.06758631	-0.8719909
C	3.12650354	0.32239877	-1.5437152
H	3.2972189	1.3634906	-1.8505993
H	3.84720725	0.10428882	-0.7419966
H	3.3887076	-0.3133927	-2.3958513
N	-0.6970099	-0.0336685	-0.044157
C	-0.9989831	1.25979351	0.45145113
C	0.07843291	2.0063187	0.94306282
N	1.37943946	1.37653271	0.93007161
C	1.55061181	0.53011316	2.12679038
H	2.49912893	0.00063053	2.04404828
H	1.55196997	1.14704796	3.03313043
H	0.74310738	-0.1969823	2.18503095
C	2.48253566	2.33707746	0.8624091
H	3.40670468	1.78137763	0.71895383
H	2.33540184	3.00316348	0.014128
H	2.5657915	2.92175247	1.78438391
C	-0.1432468	3.28820879	1.41662069
C	-1.4201541	3.83587944	1.38460829
C	-2.4809261	3.10175314	0.88120743
C	-2.2710013	1.81249618	0.41513233
H	-3.0885417	1.25082012	-0.0212475
H	-3.4724283	3.53556584	0.83115227
H	-1.5767542	4.84592592	1.74413142
H	0.67850848	3.87585782	1.80347457
C	-1.5616976	-0.9647777	0.12453659
C	-1.3628365	-2.3605536	-0.2330796
C	-2.4405336	-3.1699645	-0.602611
C	-2.2780376	-4.5144788	-0.8829929
C	-1.0165332	-5.0808975	-0.7755747
C	0.0685742	-4.3040151	-0.3930178
C	-0.1051548	-2.960048	-0.1259823
H	0.7336409	-2.3359961	0.16271211
H	1.05250097	-4.7480439	-0.3021257
H	-0.8877326	-6.1352603	-0.9906665
H	-3.128387	-5.1114602	-1.1865247
H	-2.506717	-0.7241053	0.60964697
C	0.90682292	-1.052701	-2.5251146
H	1.21090788	-0.5181631	-3.4324627
H	1.47419647	-1.9908113	-2.4982231
H	-0.1544499	-1.3098206	-2.6232795
Br	-4.1733005	-2.4284525	-0.7905484

2Br .xyz coordinates

Pt	-0.147402	0.05335708	-2.3452338
C	1.01985946	1.69790112	-2.2096883
H	0.83911421	2.25005564	-1.2837319
H	2.07474684	1.40214035	-2.2379849
H	0.84112538	2.37025816	-3.0537818
N	-0.7115253	0.04693426	-0.2943801
C	-0.9025966	1.32007951	0.32347632
C	-0.1174777	1.72586034	1.42038367
N	0.8807241	0.88679756	1.93761248
C	1.35405598	1.18988029	3.26735186
H	1.95685861	0.34982895	3.6175267
H	1.98186228	2.09354336	3.31075649
H	0.51221767	1.31947511	3.94894198
C	1.97421857	0.5654015	1.03709016
H	1.6035327	0.26717569	0.05803622
H	2.64950621	1.42264527	0.89883337
H	2.54857261	-0.2629106	1.45685949
C	-0.4038216	2.97257735	1.98222956
C	-1.4111148	3.78284369	1.48088201
C	-2.152703	3.37853984	0.38288957
C	-1.8833855	2.14844563	-0.1951198
H	-2.4412362	1.80138502	-1.0569944
H	-2.9336155	4.01000706	-0.0230442
H	-1.6016566	4.74316131	1.94641935
H	0.18237202	3.32115426	2.82230242
C	-0.8975241	-0.9542635	0.48033958
C	-0.7057158	-2.3676026	0.17211512
C	-0.9092934	-3.0043103	-1.0516756
C	-0.6939665	-4.3584454	-1.2181057
C	-0.2519922	-5.1156776	-0.1421767
C	-0.0544132	-4.5183514	1.09312526
C	-0.2966768	-3.165462	1.24576924
H	-0.1584079	-2.6924492	2.21210075
H	0.27882017	-5.1066155	1.93919363
H	-0.073491	-6.1759731	-0.2757549
H	-0.8692982	-4.8194458	-2.1818548
H	-1.1714579	-0.757714	1.51601164
C	0.34463354	0.05093366	-4.3146834
H	-0.2035939	0.83546634	-4.8516352
H	1.41169074	0.24512342	-4.4707673
H	0.11004272	-0.9057972	-4.7964078
Br	-1.571589	-2.0459544	-2.5472243

3Br .xyz coordinates (TS)

Pt	-0.4188226	-0.2844968	-2.1192799
C	1.22200541	0.89083033	-2.2030429
H	1.13746355	1.69440421	-1.4651559
H	2.1174684	0.30192302	-1.9799447
H	1.34477729	1.33138721	-3.1953489
N	-0.8053748	0.12467068	-0.0489036
C	-0.9249806	1.43714628	0.47054768
C	-0.1956749	1.88516748	1.59274307
N	0.72028066	1.0442931	2.24185391
C	1.1292997	1.43284195	3.57078201
H	1.66604281	0.59692201	4.02333591
H	1.79943546	2.30661406	3.58237181
H	0.25671608	1.65645241	4.1859783
C	1.84896893	0.59779083	1.44313139
H	1.52718087	0.25390338	0.46316689
H	2.58391899	1.4034827	1.29845192
H	2.34261387	-0.2316969	1.95367335
C	-0.4456573	3.18521836	2.03822713
C	-1.3558255	4.01411147	1.39985858
C	-2.0270793	3.57579742	0.27034588
C	-1.7933265	2.29159549	-0.1924966
H	-2.3006859	1.91386867	-1.0731033
H	-2.7256835	4.22184464	-0.247173
H	-1.5194338	5.01598727	1.78027458
H	0.09544542	3.56264483	2.8957674
C	-0.9026464	-0.8825891	0.73728249
C	-0.7029036	-2.2080371	0.19233975
C	-1.0080917	-2.4833479	-1.153529
C	-0.571246	-3.6568094	-1.7529706
C	0.17939623	-4.5559922	-1.0107734
C	0.44770799	-4.3272752	0.33352814
C	0.00359196	-3.1569362	0.92634671
H	0.24410847	-2.9356347	1.96096499
H	1.00881876	-5.0512769	0.9108493
H	0.52963287	-5.4642855	-1.4877376
H	-0.830973	-3.8702177	-2.7815788
H	-1.0433985	-0.7509135	1.80736147
C	-0.0563662	-0.7397359	-4.0635948
H	-0.2906935	0.11051977	-4.7153569
H	0.99438369	-0.9975226	-4.235124
H	-0.6692509	-1.589062	-4.3866307
Br	-2.6111429	-1.6635881	-1.967438

4Br .xyz coordinates

Pt	-0.732005	-0.3916367	-2.0738435
C	1.23805557	0.05141196	-2.3405567
H	1.25165947	1.0865418	-2.7005971
H	1.7875278	-0.0096644	-1.4004852
H	1.71095079	-0.5953345	-3.0787801
N	-0.5646883	0.17174581	0.01493727
C	-0.747642	1.47636866	0.50971819
C	-0.1947526	1.98470043	1.71335543
N	0.68242448	1.22350126	2.49760341
C	0.86278741	1.62938071	3.87185395
H	1.3878197	0.82980237	4.39804426
H	1.45896332	2.54834723	3.97930579
H	-0.1045722	1.7815462	4.3514479
C	1.9494033	0.8848802	1.87175574
H	1.81121631	0.60886279	0.82903697
H	2.64822675	1.73281901	1.90714104
H	2.40306066	0.04043254	2.39542192
C	-0.5679792	3.27212144	2.1014129
C	-1.4117454	4.05904585	1.3334522
C	-1.8954703	3.58204665	0.12749979
C	-1.5536734	2.30260755	-0.2701141
H	-1.9700662	1.89492646	-1.18422
H	-2.5441055	4.18769626	-0.4931823
H	-1.6706935	5.05440349	1.67588406
H	-0.1681877	3.67779304	3.02110717
C	-0.2852714	-0.8534912	0.74134134
C	-0.0937879	-2.1077442	0.06990081
C	-0.2031818	-2.1117001	-1.3311408
C	0.01610956	-3.2750921	-2.0436383
C	0.32630832	-4.4420142	-1.3506159
C	0.42648509	-4.454792	0.03558188
C	0.2192504	-3.2873394	0.74654053
H	0.2982469	-3.2718098	1.82837399
H	0.6688172	-5.3740572	0.55460947
H	0.49173215	-5.3554097	-1.9112137
H	-0.0574549	-3.2925213	-3.1220658
H	-0.1741682	-0.7736306	1.81830565
C	-0.8324355	-0.922392	-4.0287659
H	-1.0895944	-0.0076018	-4.5779008
H	0.1138064	-1.3067506	-4.4119771
H	-1.6240501	-1.6543872	-4.1917092
Br	-3.2673272	-0.5936145	-1.8828263

M3 .xyz coordinates

Pt	1.82077161	0.04585462	-0.644014
C	3.86547501	-0.0744877	-0.6485725
H	4.27555258	0.53426995	0.16534203
H	4.21300336	-1.1005222	-0.5065915
H	4.26164003	0.30431012	-1.5930319
N	-0.2357155	0.13227109	-0.6426082
C	-0.8158214	1.10760869	0.16246962
C	0.0527703	1.89096883	0.9376368
N	1.49803257	1.6434427	0.93791342
C	1.91105317	1.18862359	2.27894411
H	2.96732852	0.92298528	2.24639996
H	1.76716225	1.98527917	3.01410501
H	1.32722269	0.3203492	2.57226343
C	2.2465076	2.87671221	0.62620207
H	3.30341575	2.62258139	0.55404522
H	1.9120211	3.27113653	-0.328954
H	2.11604896	3.62264611	1.41454522
C	-0.4773632	2.89452381	1.73652714
C	-1.8413957	3.12683837	1.77476258
C	-2.6992567	2.34869012	1.00802709
C	-2.1890603	1.34744055	0.20697489
H	-2.8573558	0.7484187	-0.3989431
H	-3.7676085	2.52712829	1.03145009
H	-2.2338961	3.91706575	2.40330233
H	0.18104434	3.50584501	2.34071971
C	-0.797575	-0.7020577	-1.4459582
C	0.08882675	-1.5443729	-2.2051174
C	1.48034481	-1.351376	-2.0000046
C	2.36376069	-2.1260014	-2.7313739
C	1.8869629	-3.0620675	-3.6445679
C	0.52264987	-3.2469831	-3.8438161
C	-0.3792034	-2.4858889	-3.1235453
H	-1.4476158	-2.6098053	-3.2673457
H	0.1709115	-3.9801358	-4.5596037
H	2.59498031	-3.6574732	-4.2113246
H	3.43216549	-2.0041286	-2.6007577
H	-1.8768896	-0.7641682	-1.5690315
C	1.85820074	-1.4646081	0.74530192
H	2.0909279	-2.4113154	0.25831667
H	2.62335589	-1.2589682	1.49317706
H	0.88064514	-1.5427889	1.22686969
Br	1.80475798	1.80709999	-2.568416

2H .xyz coordinates

Pt	0.4908591	0.08772528	0.09942337
C	1.37559673	1.85908169	-0.2841736
H	0.77411571	2.41500864	-1.0108057
H	1.45303228	2.45154662	0.6323015
H	2.37332862	1.72209696	-0.7103665
N	-0.0738727	0.47801577	2.09729383
C	-0.2671789	1.7727058	2.64232434
C	0.49707245	2.22920619	3.73470056
N	1.49223414	1.41499323	4.29321035
C	1.94185392	1.75916334	5.62101969
H	2.54402295	0.93354781	6.00505806
H	2.56242356	2.66848177	5.64866964
H	1.0875991	1.90123966	6.28434203
C	2.60294966	1.08786067	3.41545056
H	2.25020328	0.77782491	2.43280591
H	3.27773103	1.94652566	3.28205258
H	3.17273371	0.2643832	3.8507993
C	0.19818167	3.49674295	4.23800487
C	-0.7954842	4.28506683	3.67667548
C	-1.5037924	3.83862334	2.5730485
C	-1.223911	2.58415054	2.05497805
H	-1.7610669	2.20178096	1.19455626
H	-2.2689769	4.45567656	2.11802257
H	-0.998627	5.2633087	4.09739391
H	0.76340304	3.8793001	5.07781973
C	-0.2151728	-0.5362211	2.87078351
C	0.01053921	-1.8540748	2.32066918
C	-0.0399481	-2.0107841	0.9223329
C	0.26935628	-3.2256636	0.33204201
C	0.65563457	-4.2939024	1.127728
C	0.70741764	-4.1645122	2.50810371
C	0.3837633	-2.9515533	3.09435778
Cl	0.50455522	-2.8049179	4.81281677
H	1.0063122	-4.9970335	3.13205242
H	0.90857173	-5.2451951	0.67479383
H	0.20081895	-3.3343592	-0.7429945
H	-0.4725753	-0.4092552	3.91759416
C	1.00196408	-0.4532267	-1.7912775
H	1.06319181	0.38919532	-2.4870621
H	1.98489602	-0.9417785	-1.7842604
H	0.28200772	-1.1739468	-2.1986432
H	-0.6987435	-1.3050882	0.31051819

3H .xyz coordinates (TS)

Pt	-0.0553933	-0.4167711	-1.9928604
C	0.28449096	1.54866186	-2.5990721
H	0.21697244	1.71793832	-3.676552
H	-0.3641545	2.26699715	-2.0896579
H	1.31849683	1.78845622	-2.3000948
N	-0.6601047	0.05776013	0.01336212
C	-0.8782652	1.3416612	0.56725299
C	-0.0635636	1.83273944	1.60629712
N	1.00005815	1.05761698	2.09502652
C	1.50889754	1.41113822	3.3998061
H	2.17094061	0.6125011	3.73958185
H	2.0848565	2.34958053	3.40351355
H	0.68728709	1.50520234	4.11099667
C	2.0735329	0.80936497	1.15040041
H	1.68156244	0.44558922	0.20200685
H	2.66043778	1.71809822	0.95031226
H	2.74011229	0.04694871	1.55737449
C	-0.3841998	3.08241042	2.13860113
C	-1.4534629	3.82329312	1.65674477
C	-2.2229642	3.3415117	0.6108414
C	-1.9226274	2.10241346	0.06689415
H	-2.5140413	1.69290205	-0.7437714
H	-3.0532501	3.91815945	0.22154131
H	-1.6725722	4.78934173	2.09708619
H	0.21959582	3.48761029	2.94016003
C	-0.7885087	-0.9708083	0.77313721
C	-0.5570944	-2.2767608	0.21044428
C	-0.2261593	-2.3094891	-1.1655502
C	0.0024561	-3.5555178	-1.7365925
C	-0.0845408	-4.7221291	-0.9850728
C	-0.4055753	-4.6843342	0.36514183
C	-0.6400167	-3.4564415	0.95455979
Cl	-1.0322081	-3.4176434	2.64560965
H	-0.4727635	-5.5913872	0.95171645
H	0.09757265	-5.6837124	-1.453754
H	0.25541069	-3.633162	-2.7884121
H	-1.0730684	-0.8466728	1.81464292
C	0.61733629	-1.0224003	-3.8083524
H	0.44624323	-0.2889925	-4.5975966
H	1.70184112	-1.1576267	-3.6964045
H	0.1884169	-1.978149	-4.1159916
H	-1.4319839	-0.7358987	-2.4933166

4H .xyz coordinates

Pt	0.62104057	-0.0418454	0.24552405
C	0.83337795	2.30864614	-0.5536324
H	1.14676269	2.56552886	-1.56324
H	0.56711722	3.17993881	0.03983277
H	1.68354111	1.80502291	-0.0572691
N	-0.0148169	0.43688864	2.19974631
C	-0.2256546	1.72804462	2.73693736
C	0.59150378	2.23369222	3.76830979
N	1.64754727	1.46455953	4.27510068
C	2.1726606	1.85592413	5.56184456
H	2.82976196	1.06192249	5.92128739
H	2.75912578	2.78746472	5.52782274
H	1.36028714	1.98301259	6.27863345
C	2.70901466	1.15561405	3.33260117
H	2.30316284	0.78953973	2.38979871
H	3.33128938	2.03852736	3.12191723
H	3.34662784	0.37635074	3.75446357
C	0.27780694	3.49746454	4.27266955
C	-0.7831458	4.23731328	3.77113602
C	-1.5529555	3.74148811	2.73196313
C	-1.2611655	2.48809436	2.21665685
H	-1.8547961	2.06477167	1.41421589
H	-2.3776346	4.31643159	2.32846942
H	-0.9951765	5.21406444	4.1909643
H	0.88217979	3.91548523	5.06709565
C	-0.152527	-0.5928241	2.956294
C	0.09959284	-1.8747876	2.35786914
C	0.48870813	-1.8435752	0.99192495
C	0.73367998	-3.0609698	0.36277673
C	0.60240017	-4.258336	1.05327321
C	0.22766612	-4.2895007	2.39037687
C	-0.0208658	-3.0914729	3.03104549
Cl	-0.4875833	-3.127056	4.70158266
H	0.12942954	-5.2251479	2.92502658
H	0.79732901	-5.1948112	0.54163791
H	1.03108665	-3.0850051	-0.6773137
H	-0.4491152	-0.4864885	3.99562667
C	1.25141588	-0.6711588	-1.5843802
H	1.44708705	0.17366545	-2.2548179
H	2.17554745	-1.2545855	-1.5081794
H	0.50092967	-1.3086863	-2.0652788
H	-0.0840801	1.68648499	-0.650456

M4A + CH_4 .xyz coordinates

H	0.53752468	-4.0615012	-4.0648936
H	-1.8726081	-4.1157518	-3.5082019
C	0.13652918	-3.335841	-3.3648521
C	-1.2195911	-3.3776847	-3.0610601
H	2.02921818	-2.3918742	-3.0559397
C	0.97778771	-2.3905853	-2.7929196
H	3.30065157	-0.2572953	-2.759019
C	-1.7239136	-2.4514531	-2.1696171
C	0.49793642	-1.4445408	-1.8907881
Cl	-3.4193682	-2.5079599	-1.7929438
C	3.28152348	-0.3457572	-1.6649065
C	-0.9002295	-1.4891483	-1.5799386
H	3.62423906	-1.3618645	-1.4300746
H	4.04128721	0.34556176	-1.2751671
H	1.64860491	3.11862237	-1.1611703
Pt	1.4104751	0.00451866	-0.9193089
H	3.29166569	2.49025749	-0.9152795
C	-1.3628838	-0.496645	-0.6460476
H	-2.4007166	-0.4324447	-0.3337283
C	2.38460302	2.82490628	-0.4147036
N	-0.4527589	0.30862675	-0.2088546
H	2.6234738	3.68211631	0.22307641
H	6.00713601	1.74694551	-0.3639534
N	1.84899483	1.70217336	0.377324
H	5.68767144	3.42997624	0.11732564
C	-0.5944628	1.36094843	0.6950796
H	7.35727307	2.81233287	0.09718506
C	6.3163496	2.55765228	0.29587749
H	-2.703606	1.17314552	1.06240564
H	3.73316592	0.97310255	0.9176994
C	0.57343189	2.07633449	0.99632121
C	-1.8003609	1.72449684	1.29294659
C	2.82527018	1.31357246	1.4123662
C	0.51045849	3.1385195	1.88605544
H	2.41186237	0.4998942	2.00583611
H	6.21091079	2.24090699	1.33352836
C	-1.8476811	2.78462816	2.17695171
H	3.06887857	2.15787078	2.06534759
H	1.40746898	3.69665858	2.1248863
C	-0.6913838	3.49477847	2.47502825
H	-2.7898803	3.0594234	2.6360027
H	-0.7245458	4.32701119	3.16782053

C-Cl OA NEB-TS Imaginary Mode = -160.96 cm-1

NEB-TS_converged: Selected coordination sphere distances

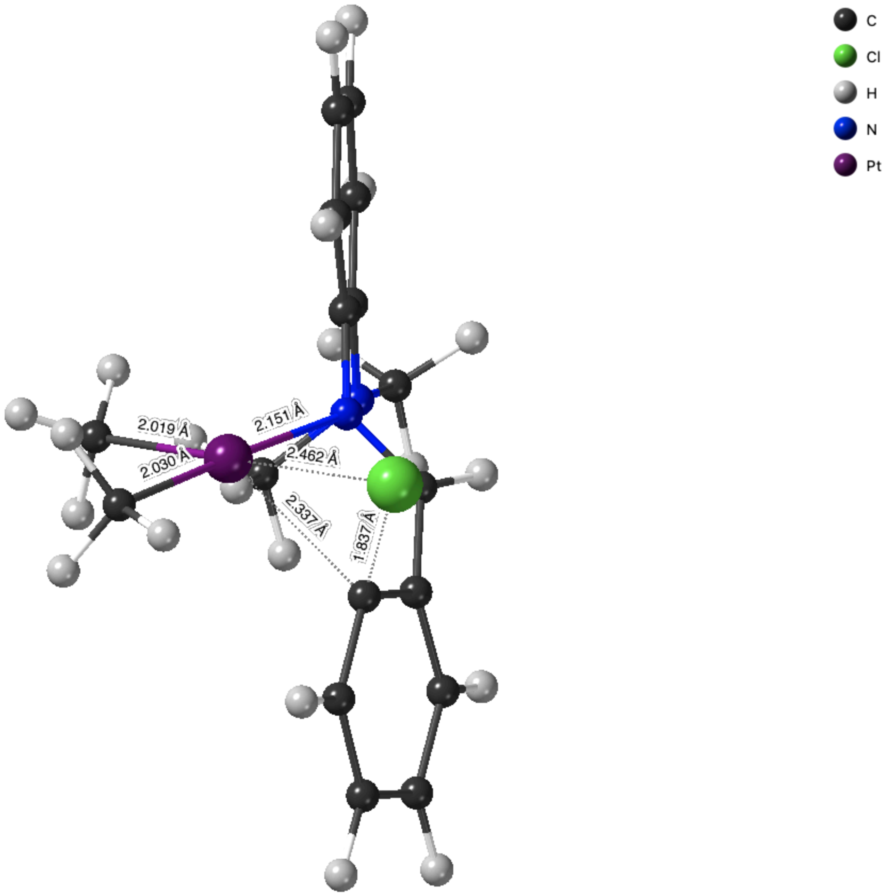

C-Br OA NEB-TS

Imaginary mode = -161.13 cm-1

NEB-TS_converged: Selected coordination sphere distances

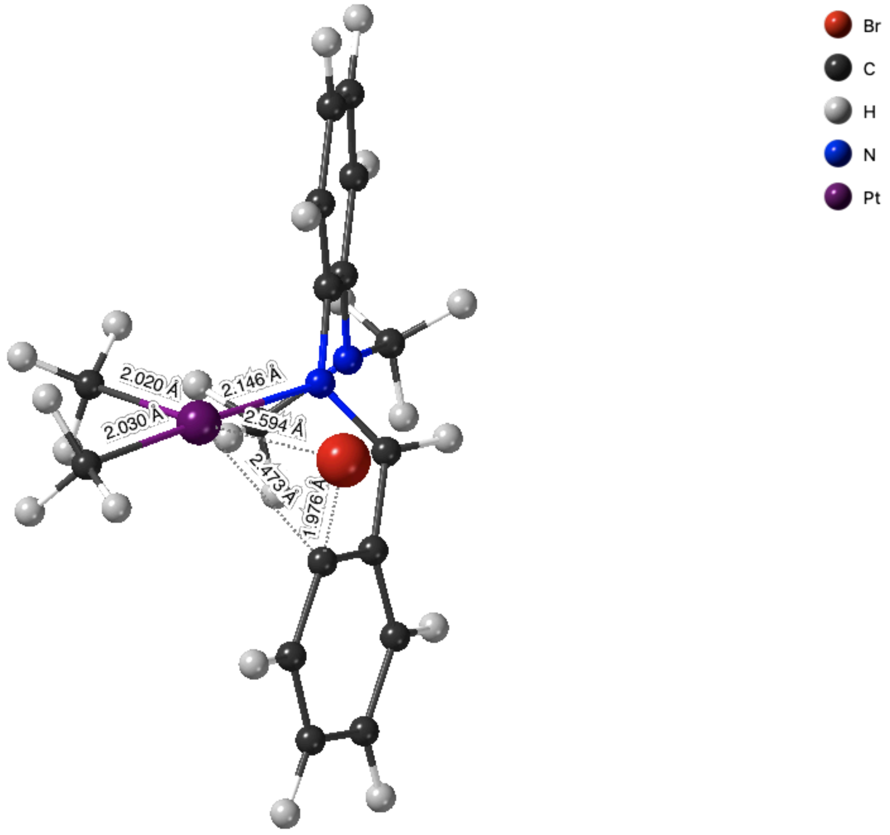

C-H OA NEB-TS

Imaginary mode = -58.84 cm-1

NEB-TS_converged: Selected coordination sphere distances

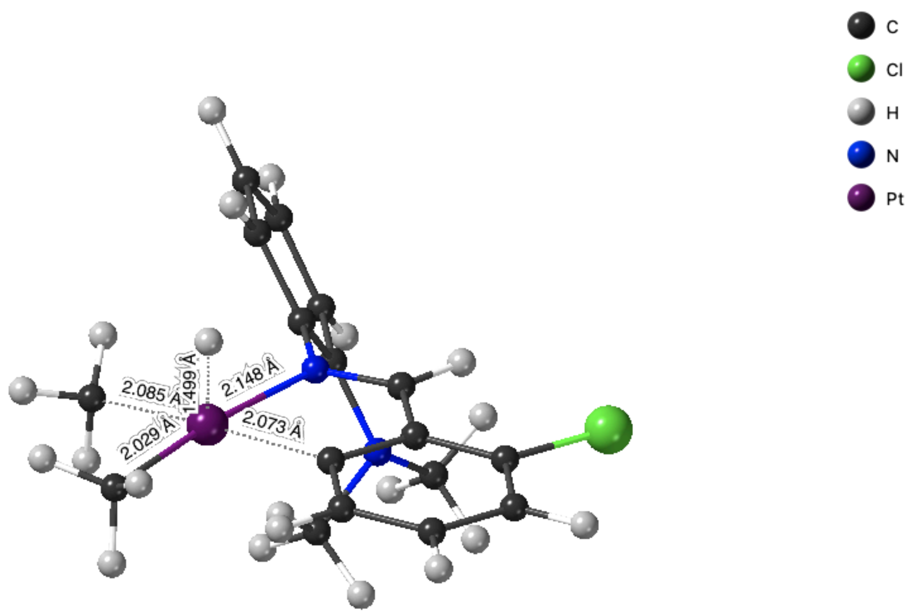

Table S11 M3 Parameters

```
Bond precision:   C-C = 0.0048 A          Wavelength=0.71073

Cell:             a=10.7675(11)     b=15.4962(16)       c=20.390(2)
                  alpha=90          beta=101.792(2)     gamma=90
Temperature:      125 K

                 Calculated                   Reported
Volume           3330.4(6)                    3330.4(6)
Space group      P 21/c                       P 1 21/c 1
Hall group       -P 2ybc                      -P 2ybc
Moiety formula   C17 H21 Br N2 Pt             C17 H21 Br N2 Pt
Sum formula      C17 H21 Br N2 Pt             C17 H21 Br N2 Pt
Mr               528.34                       528.36
Dx,g cm-3        2.108                        2.108
Z                8                            8
Mu (mm-1)        10.821                       10.821
F000             2000.0                       2000.0
F000'            1985.89
h,k,lmax         15,22,29                     15,22,29
Nref             10198                        10176
Tmin,Tmax        0.229,0.339                  0.320,0.410
Tmin'            0.146

Correction method= # Reported T Limits: Tmin=0.320 Tmax=0.410
AbsCorr = MULTI-SCAN

Data completeness= 0.998           Theta(max)= 30.540

                                                     wR2(reflections)=
R(reflections)= 0.0211( 8977)                        0.0459( 10176)
S = 1.028                 Npar= 387
```

Table S12 M4 Parameters

Bond precision:	C-C = 0.0041 A		Wavelength=0.71073
Cell:	a=10.7262(9)	b=15.4664(13)	c=20.2185(17)
	alpha=90	beta=102.293(1)	gamma=90
Temperature:	125 K		

	Calculated	Reported
Volume	3277.3(5)	3277.3(5)
Space group	P 21/c	P 1 21/c 1
Hall group	-P 2ybc	-P 2ybc
Moiety formula	C17 H21 Cl N2 Pt	C17 H21 Cl N2 Pt
Sum formula	C17 H21 Cl N2 Pt	C17 H21 Cl N2 Pt
Mr	483.89	483.90
Dx,g cm-3	1.961	1.961
Z	8	8
Mu (mm-1)	8.721	8.721
F000	1856.0	1856.0
F000’	1844.83	
h,k,lmax	15,22,29	15,22,29
Nref	10215	10169
Tmin,Tmax	0.365,0.543	0.340,0.580
Tmin’	0.109	

Correction method= # Reported T Limits: Tmin=0.340 Tmax=0.580
AbsCorr = MULTI-SCAN

Data completeness= 0.995   Theta(max)= 30.750

R(reflections)= 0.0200(9033)   wR2(reflections)= 0.0445(10169)

S = 1.031   Npar= 387

Table S13 M4A Parameters

Bond precision:	C-C = 0.0058 A		Wavelength=0.71073
Cell:	a=8.6805(10)	b=15.3378(18)	c=11.1980(13)
	alpha=90	beta=94.482(2)	gamma=90
Temperature:	125 K		

	Calculated	Reported
Volume	1486.3(3)	1486.3(3)
Space group	P 21/c	P 1 21/c 1
Hall group	-P 2ybc	-P 2ybc
Moiety formula	C16 H17 Cl N2 Pt	C16 H17 Cl N2 Pt
Sum formula	C16 H17 Cl N2 Pt	C16 H17 Cl N2 Pt
Mr	467.85	467.85
Dx,g cm-3	2.091	2.091
Z	4	4
Mu (mm-1)	9.611	9.611
F000	888.0	888.0
F000’	882.43	
h,k,lmax	12,21,16	12,21,15
Nref	4538	4521
Tmin,Tmax	0.451,0.750	0.400,0.760
Tmin’	0.120	

Correction method= # Reported T Limits: Tmin=0.400 Tmax=0.760
AbsCorr = MULTI-SCAN

Data completeness= 0.996 Theta(max)= 30.520

R(reflections)= 0.0282(3356) wR2(reflections)= 0.0578(4521)

S = 1.002 Npar= 184

M3 Bond lengths and angles

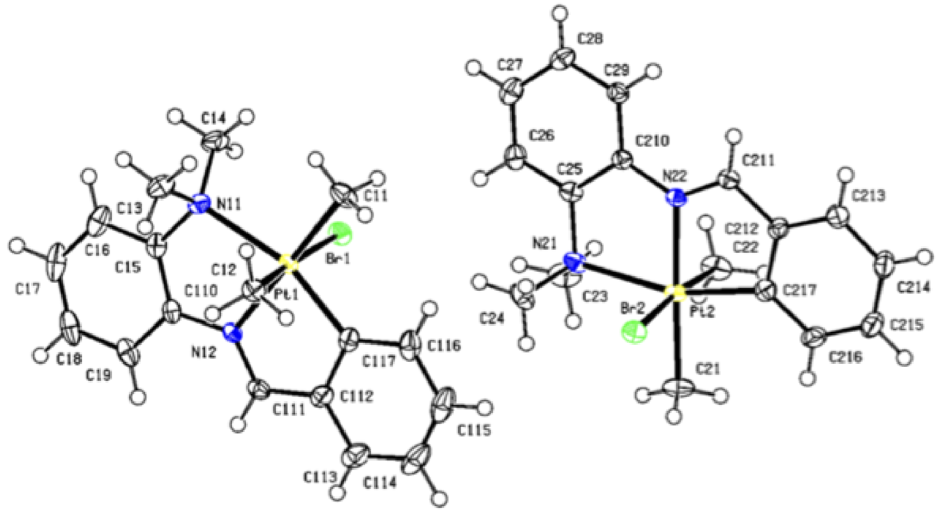

Pt1 C117 . 2.002(3)
Pt1 C11 . 2.055(3)
Pt1 N12 . 2.055(2)
Pt1 C12 . 2.063(3)
Pt1 N11 . 2.253(2)
Pt1 Br1 . 2.5927(4)
Pt2 C217 . 1.999(3)
Pt2 C21 . 2.050(3)
Pt2 N22 . 2.052(2)
Pt2 C22 . 2.068(3)
Pt2 N21 . 2.262(2)
Pt2 Br2 . 2.6037(4)
N11 C15 . 1.481(4)
N11 C14 . 1.493(4)
N11 C13 . 1.500(4)
N12 C111 . 1.290(4)
N12 C110 . 1.411(4)
N21 C25 . 1.482(4)
N21 C24 . 1.494(4)
N21 C23 . 1.498(4)
N22 C211 . 1.293(4)
N22 C210 . 1.408(4)
C15 C16 . 1.395(4)
C15 C110 . 1.403(4)
C16 C17 . 1.388(5)
C17 C18 . 1.379(5)
C18 C19 . 1.379(5)
C19 C110 . 1.402(4)
C25 C26 . 1.391(4)
C25 C210 . 1.397(4)

C26 C27 . 1.385(4)
C27 C28 . 1.390(5)
C28 C29 . 1.385(4)
C29 C210 . 1.399(4)
C111 C112 . 1.438(4)
C112 C113 . 1.405(4)
C112 C117 . 1.418(4)
C113 C114 . 1.378(5)
C114 C115 . 1.377(6)
C115 C116 . 1.397(5)
C116 C117 . 1.393(4)
C211 C212 . 1.445(4)
C212 C213 . 1.404(4)
C212 C217 . 1.422(4)
C213 C214 . 1.379(4)
C214 C215 . 1.388(5)
C215 C216 . 1.390(5)
C216 C217 . 1.388(4)

C117 Pt1 C11 . . 98.95(13)
C117 Pt1 N12 . . 81.18(11)
C11 Pt1 N12 . . 179.70(12)
C117 Pt1 C12 . . 86.28(12)
C11 Pt1 C12 . . 86.88(13)
N12 Pt1 C12 . . 92.86(11)
C117 Pt1 N11 . . 161.55(11)
C11 Pt1 N11 . . 99.43(12)
N12 Pt1 N11 . . 80.43(9)
C12 Pt1 N11 . . 93.13(11)
C117 Pt1 Br1 . . 90.35(8)
C11 Pt1 Br1 . . 91.19(10)
N12 Pt1 Br1 . . 89.08(6)
C12 Pt1 Br1 . . 175.81(9)
N11 Pt1 Br1 . . 90.86(6)
C217 Pt2 C21 . . 97.22(13)
C217 Pt2 N22 . . 81.50(11)
C21 Pt2 N22 . . 177.75(12)
C217 Pt2 C22 . . 88.46(13)
C21 Pt2 C22 . . 87.31(13)
N22 Pt2 C22 . . 90.80(11)
C217 Pt2 N21 . . 161.67(10)
C21 Pt2 N21 . . 101.07(12)
N22 Pt2 N21 . . 80.18(9)
C22 Pt2 N21 . . 91.28(12)
C217 Pt2 Br2 . . 87.48(8)
C21 Pt2 Br2 . . 89.54(9)

N22 Pt2 Br2 . . 92.25(6)
C22 Pt2 Br2 . . 174.51(10)
N21 Pt2 Br2 . . 93.73(6)
C15 N11 C14 . . 111.5(2)
C15 N11 C13 . . 106.8(2)
C14 N11 C13 . . 107.6(2)
C15 N11 Pt1 . . 105.65(17)
C14 N11 Pt1 . . 113.07(19)
C13 N11 Pt1 . . 112.09(18)
C111 N12 C110 . . 128.4(3)
C111 N12 Pt1 . . 115.1(2)
C110 N12 Pt1 . . 116.43(18)
C25 N21 C24 . . 109.1(2)
C25 N21 C23 . . 110.0(2)
C24 N21 C23 . . 107.2(2)
C25 N21 Pt2 . . 105.62(17)
C24 N21 Pt2 . . 112.09(18)
C23 N21 Pt2 . . 112.8(2)
C211 N22 C210 . . 128.2(2)
C211 N22 Pt2 . . 115.04(19)
C210 N22 Pt2 . . 116.68(18)
C16 C15 C110 . . 119.0(3)
C16 C15 N11 . . 120.6(3)
C110 C15 N11 . . 120.3(2)
C17 C16 C15 . . 120.1(3)
C18 C17 C16 . . 120.6(3)
C19 C18 C17 . . 120.4(3)
C18 C19 C110 . . 119.7(3)
C26 C25 C210 . . 119.3(3)
C26 C25 N21 . . 120.3(3)
C210 C25 N21 . . 120.4(2)
C27 C26 C25 . . 120.2(3)
C26 C27 C28 . . 120.7(3)
C29 C28 C27 . . 119.6(3)
C28 C29 C210 . . 120.0(3)
C19 C110 C15 . . 120.1(3)
C19 C110 N12 . . 123.3(3)
C15 C110 N12 . . 116.6(2)
N12 C111 C112 . . 115.6(3)
C113 C112 C117 . . 121.1(3)
C113 C112 C111 . . 122.0(3)
C117 C112 C111 . . 116.9(3)
C114 C113 C112 . . 119.4(3)
C115 C114 C113 . . 120.3(3)
C114 C115 C116 . . 121.0(3)
C117 C116 C115 . . 120.5(3)

C116 C117 C112 . . 117.7(3)
C116 C117 Pt1 . . 131.1(3)
C112 C117 Pt1 . . 111.2(2)
C25 C210 C29 . . 120.2(3)
C25 C210 N22 . . 116.8(2)
C29 C210 N22 . . 123.0(3)
N22 C211 C212 . . 115.5(2)
C213 C212 C217 . . 121.0(3)
C213 C212 C211 . . 122.2(3)
C217 C212 C211 . . 116.8(3)
C214 C213 C212 . . 119.5(3)
C213 C214 C215 . . 119.7(3)
C214 C215 C216 . . 121.4(3)
C217 C216 C215 . . 120.4(3)
C216 C217 C212 . . 117.9(3)
C216 C217 Pt2 . . 131.0(2)
C212 C217 Pt2 . . 111.0(2)

M4 Bond lengths and angles

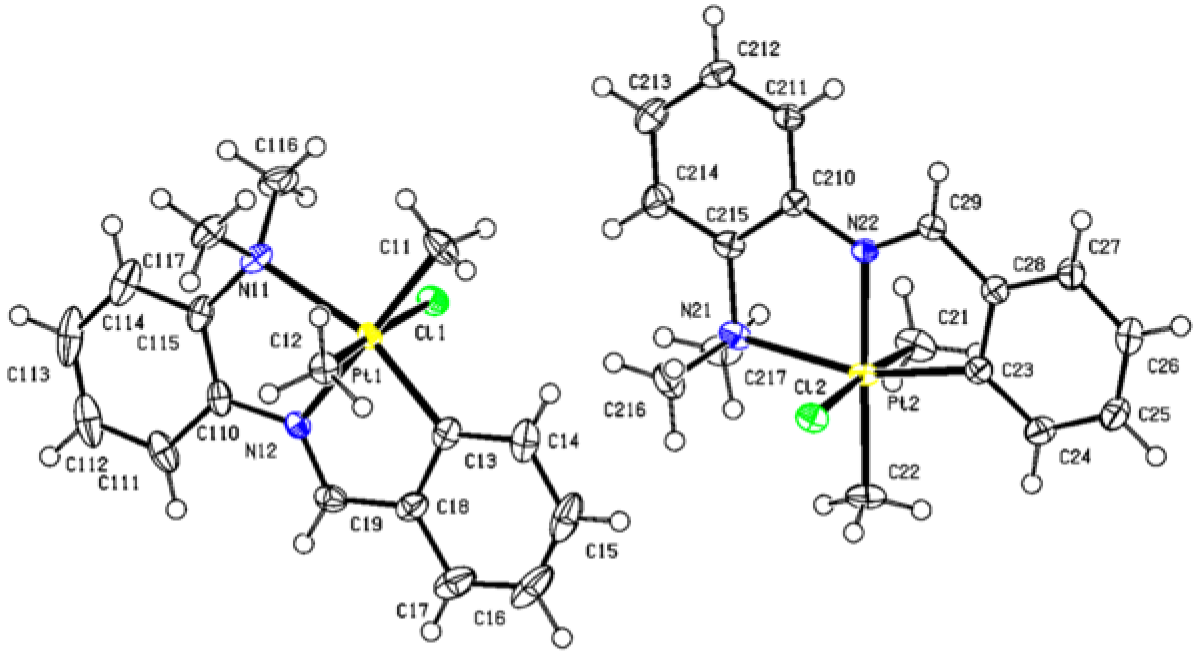

Pt1 C13 . 1.998(3)
Pt1 N12 . 2.0515(19)
Pt1 C11 . 2.056(3)
Pt1 C12 . 2.061(2)
Pt1 N11 . 2.249(2)

Pt1 Cl1 . 2.4751(6)
Pt2 C23 . 2.000(3)
Pt2 C22 . 2.050(3)
Pt2 N22 . 2.0563(19)
Pt2 C21 . 2.062(3)
Pt2 N21 . 2.265(2)
Pt2 Cl2 . 2.4754(6)
N11 C115 . 1.483(3)
N11 C116 . 1.493(4)
N11 C117 . 1.504(3)
N12 C19 . 1.293(3)
N12 C110 . 1.415(3)
N21 C215 . 1.484(3)
N21 C217 . 1.491(3)
N21 C216 . 1.491(3)
N22 C29 . 1.296(3)
N22 C210 . 1.409(3)
C13 C14 . 1.399(4)
C13 C18 . 1.421(4)
C14 C15 . 1.398(4)
C15 C16 . 1.369(5)
C16 C17 . 1.373(5)
C17 C18 . 1.407(4)
C18 C19 . 1.444(4)
C23 C24 . 1.391(4)
C23 C28 . 1.416(3)
C24 C25 . 1.394(4)
C25 C26 . 1.385(4)
C26 C27 . 1.377(4)
C27 C28 . 1.402(3)
C28 C29 . 1.446(3)
C110 C115 . 1.394(4)
C110 C111 . 1.403(4)
C111 C112 . 1.385(4)
C112 C113 . 1.375(5)
C113 C114 . 1.387(5)
C114 C115 . 1.400(4)
C210 C211 . 1.399(3)
C210 C215 . 1.401(3)
C211 C212 . 1.389(4)
C212 C213 . 1.386(4)
C213 C214 . 1.383(4)
C214 C215 . 1.390(4)

C13 Pt1 N12 . . 81.15(10)
C13 Pt1 C11 . . 98.96(12)

N12 Pt1 C11 . . 179.62(10)
C13 Pt1 C12 . . 86.72(10)
N12 Pt1 C12 . . 92.18(9)
C11 Pt1 C12 . . 87.47(11)
C13 Pt1 N11 . . 161.67(10)
N12 Pt1 N11 . . 80.58(8)
C11 Pt1 N11 . . 99.30(11)
C12 Pt1 N11 . . 92.42(9)
C13 Pt1 Cl1 . . 90.33(7)
N12 Pt1 Cl1 . . 89.39(6)
C11 Pt1 Cl1 . . 90.97(8)
C12 Pt1 Cl1 . . 176.41(8)
N11 Pt1 Cl1 . . 91.02(6)
C23 Pt2 C22 . . 97.98(11)
C23 Pt2 N22 . . 81.28(9)
C22 Pt2 N22 . . 178.43(10)
C23 Pt2 C21 . . 88.55(11)
C22 Pt2 C21 . . 87.10(11)
N22 Pt2 C21 . . 91.50(10)
C23 Pt2 N21 . . 161.57(9)
C22 Pt2 N21 . . 100.43(10)
N22 Pt2 N21 . . 80.29(8)
C21 Pt2 N21 . . 91.49(11)
C23 Pt2 Cl2 . . 87.88(7)
C22 Pt2 Cl2 . . 89.96(8)
N22 Pt2 Cl2 . . 91.39(6)
C21 Pt2 Cl2 . . 175.03(9)
N21 Pt2 Cl2 . . 92.99(6)
C115 N11 C116 . . 111.3(2)
C115 N11 C117 . . 107.2(2)
C116 N11 C117 . . 108.1(2)
C115 N11 Pt1 . . 105.60(15)
C116 N11 Pt1 . . 111.95(18)
C117 N11 Pt1 . . 112.66(16)
C19 N12 C110 . . 128.2(2)
C19 N12 Pt1 . . 115.54(17)
C110 N12 Pt1 . . 116.24(16)
C215 N21 C217 . . 109.60(19)
C215 N21 C216 . . 109.3(2)
C217 N21 C216 . . 107.8(2)
C215 N21 Pt2 . . 105.57(14)
C217 N21 Pt2 . . 113.32(18)
C216 N21 Pt2 . . 111.21(16)
C29 N22 C210 . . 128.3(2)
C29 N22 Pt2 . . 115.04(16)
C210 N22 Pt2 . . 116.64(15)

C14 C13 C18 . . 117.3(3)
C14 C13 Pt1 . . 131.3(2)
C18 C13 Pt1 . . 111.38(18)
C15 C14 C13 . . 120.4(3)
C16 C15 C14 . . 121.3(3)
C15 C16 C17 . . 120.4(3)
C16 C17 C18 . . 119.4(3)
C17 C18 C13 . . 121.2(3)
C17 C18 C19 . . 122.0(3)
C13 C18 C19 . . 116.8(2)
N12 C19 C18 . . 115.1(2)
C24 C23 C28 . . 118.1(2)
C24 C23 Pt2 . . 130.6(2)
C28 C23 Pt2 . . 111.28(18)
C23 C24 C25 . . 120.2(3)
C26 C25 C24 . . 121.0(3)
C27 C26 C25 . . 120.2(3)
C26 C27 C28 . . 119.3(2)
C27 C28 C23 . . 121.1(2)
C27 C28 C29 . . 121.9(2)
C23 C28 C29 . . 117.0(2)
N22 C29 C28 . . 115.3(2)
C115 C110 C111 . . 120.5(3)
C115 C110 N12 . . 116.8(2)
C111 C110 N12 . . 122.7(3)
C112 C111 C110 . . 119.1(3)
C113 C112 C111 . . 120.6(3)
C112 C113 C114 . . 120.9(3)
C113 C114 C115 . . 119.5(3)
C110 C115 C114 . . 119.3(3)
C110 C115 N11 . . 120.4(2)
C114 C115 N11 . . 120.2(3)
C211 C210 C215 . . 120.2(2)
C211 C210 N22 . . 123.0(2)
C215 C210 N22 . . 116.8(2)
C212 C211 C210 . . 119.7(2)
C213 C212 C211 . . 120.0(2)
C214 C213 C212 . . 120.4(3)
C213 C214 C215 . . 120.6(2)
C214 C215 C210 . . 119.1(2)
C214 C215 N21 . . 120.5(2)
C210 C215 N21 . . 120.5(2)

M4A Bond lengths and angles

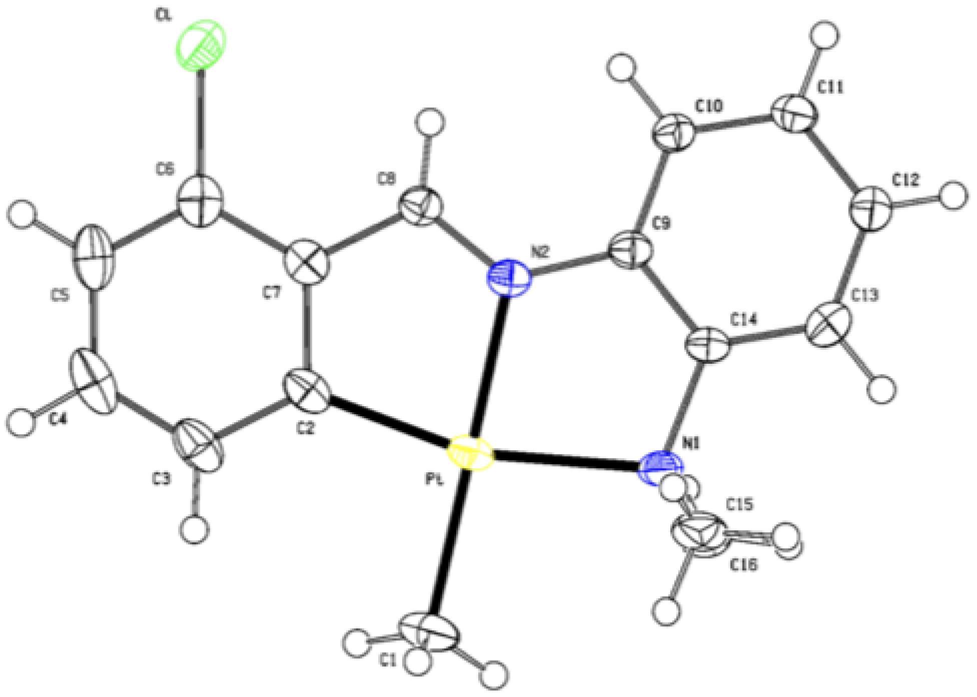

Pt C2 . 1.983(4)
Pt N2 . 2.014(3)
Pt C1 . 2.075(4)
Pt N1 . 2.169(3)
Cl C6 . 1.756(4)
N1 C16 . 1.489(5)
N1 C14 . 1.490(4)
N1 C15 . 1.495(5)
N2 C8 . 1.298(5)
N2 C9 . 1.416(5)
C2 C3 . 1.390(5)
C2 C7 . 1.446(5)
C3 C4 . 1.388(6)
C4 C5 . 1.394(7)
C5 C6 . 1.370(6)
C6 C7 . 1.395(6)
C7 C8 . 1.444(5)
C9 C14 . 1.396(5)
C9 C10 . 1.410(5)
C10 C11 . 1.392(5)
C11 C12 . 1.385(5)

C12 C13 . 1.388(6)
C13 C14 . 1.390(5)

C2 Pt N2 . . 81.92(14)
C2 Pt C1 . . 97.61(17)
N2 Pt C1 . . 179.12(16)
C2 Pt N1 . . 164.00(13)
N2 Pt N1 . . 82.09(12)
C1 Pt N1 . . 98.38(16)
C16 N1 C14 . . 109.5(3)
C16 N1 C15 . . 108.9(3)
C14 N1 C15 . . 110.0(3)
C16 N1 Pt . . 111.9(3)
C14 N1 Pt . . 106.8(2)
C15 N1 Pt . . 109.8(3)
C8 N2 C9 . . 127.8(3)
C8 N2 Pt . . 116.4(3)
C9 N2 Pt . . 115.7(2)
C3 C2 C7 . . 116.1(4)
C3 C2 Pt . . 132.9(3)
C7 C2 Pt . . 111.0(3)
C4 C3 C2 . . 122.1(4)
C3 C4 C5 . . 121.6(4)
C6 C5 C4 . . 117.6(4)
C5 C6 C7 . . 122.5(4)
C5 C6 Cl . . 118.0(3)
C7 C6 Cl . . 119.4(3)
C6 C7 C8 . . 124.2(4)
C6 C7 C2 . . 120.1(4)
C8 C7 C2 . . 115.7(3)
N2 C8 C7 . . 114.9(3)
C14 C9 C10 . . 119.8(4)
C14 C9 N2 . . 116.4(3)
C10 C9 N2 . . 123.8(3)
C11 C10 C9 . . 119.5(4)
C12 C11 C10 . . 120.0(4)
C11 C12 C13 . . 120.7(4)
C12 C13 C14 . . 120.1(4)
C13 C14 C9 . . 119.8(3)
C13 C14 N1 . . 121.2(3)
C9 C14 N1 . . 119.0(3)
